# Mono- and Diamination of 4,6-Dichloropyrimidine, 2,6-Dichloropyrazine and 1,3-Dichloroisoquinoline with Adamantane-Containing Amines †

**DOI:** 10.3390/molecules26071910

**Published:** 2021-03-29

**Authors:** Alisa D. Kharlamova, Anton S. Abel, Alexei D. Averin, Olga A. Maloshitskaya, Vitaly A. Roznyatovskiy, Evgenii N. Savelyev, Boris S. Orlinson, Ivan A. Novakov, Irina P. Beletskaya

**Affiliations:** 1 Department of Chemistry, Lomonosov Moscow State University, Leninskie Gory, 1-3, 119991 Moscow, Russia; alisa-harlamova@mail.ru (A.D.K.); antonabel@list.ru (A.S.A.); alexaveron@yandex.ru (A.D.A.); maloshitskaya@mail.ru (O.A.M.); Vit.Rozn@nmr.chem.msu.su (V.A.R.); 2 Frumkin Institute of Physical Chemistry and Electrochemistry, Russian Academy of Sciences, Leninsky Pr. 31, 119071 Moscow, Russia; 3 Volgograd State Technical University, 28 Lenina prosp., 400131 Volgograd, Russia; savelyev_en@vstu.ru (E.N.S.); orlinson@vstu.ru (B.S.O.); president@vstu.ru (I.A.N.)

**Keywords:** amines, adamantane, Pd catalysis, amination, pyrimidine, isoquinoline, pyrazine

## Abstract

*N*-heteroaryl substituted adamantane-containing amines are of substantial interest for their perspective antiviral and psychotherapeutic activities. Chlorine atom at alpha-position of *N*-heterocycles has been substituted by the amino group using convenient nucleophilic substitution reactions with a series of adamantylalkylamines. The prototropic equilibrium in these compounds was studied using NMR spectroscopy. The introduction of the second amino substituent in 4-amino-6-chloropyrimidine, 2-amino-chloropyrazine, and 1-amino-3-chloroisoquinoline was achieved using Pd(0) catalysis.

## 1. Introduction

Amino substituted pyrimidines, pyrazines, and isoquinolines attract the researchers’ interest due to their versatile biological activities stemming from the ability to form hydrogen bonds with various biomolecules, like nucleobases. Some derivatives of aminopyrimidine were found to act as inhibitors of various ferments: 1-phosphatidylinositol-3-phosphate 5-kinase (FAB1B) (e.g., bacimethrin) [1,2,3], 1-acyl-*sn*-glycerol-3-phosphate acyltransferase (plsC) [4], [Pyruvate dehydrogenase (acetyl-transferring)] kinase isozyme 1 (PDHA1) (e.g., 6′-((6-aminopyrimidin-4-yl)amino)-8′-methyl-2′*H*-spiro[cyclohexane-1,3′-imidazo[1,5-a]pyridine]-1′,5′-dione) [5], and perform as multitarget RET (rearranged during transfection) inhibitors [6]. In particular, substituted 4,6-diaminopyrimidines are capable of blocking epidermal growth factor receptor (EGFR), what make them perspective for the lung cancer treatment [7]. The last five years have demonstrated that aminopyrazine derivatives constitute an important platform for the creation of the inhibitors of mutant isocitrate dehydrogenase 1 (IDH1) [8], mitogen-activated protein kinase-interacting kinases 1 and 2 (MNK1/2) [9], and serine/threonine kinase (BRAF) (e.g., 1-{4-[6-(3,4,5-Trimethoxyphenylamino)-pyrazin-2-yl]phenyl}-3-phenyl urea) [10]. 2,6-Diaminopyrazine may serve as a backbone for the creating Nav1.7 antagonists [11], while substituted aminoisoquinolines (*N*-Methyl-3-(4-methylpiperazin-1-yl)isoquinolin-1-amine) bind to the receptor 5-HT3 [12].

Adamantane group helps to increase the lipophilicity of the molecules assuring their penetration through the biological membranes. Due to this ability adamantane derivatives demonstrate a wide range of biological activities [13]. The derivatives of adamantane-containing amines are known as the remedies against the Parkinson’s disease (e.g., 1-adamantylamine) [14,15], they exhibit antitumor [16] and anti-HIV activities [16,17], and are widely renowned as influenza viral inhibitors [18]. An imposing array of the peptide derivatives containing adamantaneamines demonstrate a wide scope of biological activities [19]. The derivatives of the heterocyclic compounds bearing adamantaneamine moieties are of special interest as psychotropic agents (e.g., 2-(1-adamantyl)-*N*-1*H*-indazol-5-ylacetamide) [16,20,21], they act as 11β-HSD1 (11β-Hydroxysteroid dehydrogenase type 1) inhibitors [22,23,24], are able to elicit anti-nicotine antibodies [25], and show antiviral and antimicrobial activities [26].

In order to search for novel perspective biologically active compounds on the basis of amino substituted heterocycles a convenient method for the synthesis of ample series of such molecule bearing versatile groups is needed. Known approaches are often limited by harsh reaction conditions, thus diminishing the scope of available substrates, e.g., unsymmetrical 4,6-diaminopyrimidines were obtained using Pd-catalyzed reactions in low yields (5–27%) [11]. Another study revealed the formation of many by-products in these processes and a strong demand for a fine adjustment of the reaction conditions [27]. The methods for 1,3-diaminoisoquinolines preparation are also limited. One of the best methods envisages α-cyano-o-tolunitrile cyclization by the action of organolithium compounds [28]. They can be also synthesized by a two-step non-catalytic amination of 1,3-dichloroisoquinoline under harsh conditions, which provides quite moderate yields (up to 31%) [12]. Fluorescent 1-morpholinoisoquinoline-3-amines were synthesized from 1-bromoisoquinoline-3-amine by heating with excess morpholine [29]. Recently, an original approach has been proposed for diamino derivatives of isoquinoline and pyrimidine, consisting of the catalytic cyclization of cyanamides and ynamides in the presence of gold complexes [30].

**Scheme 1:**
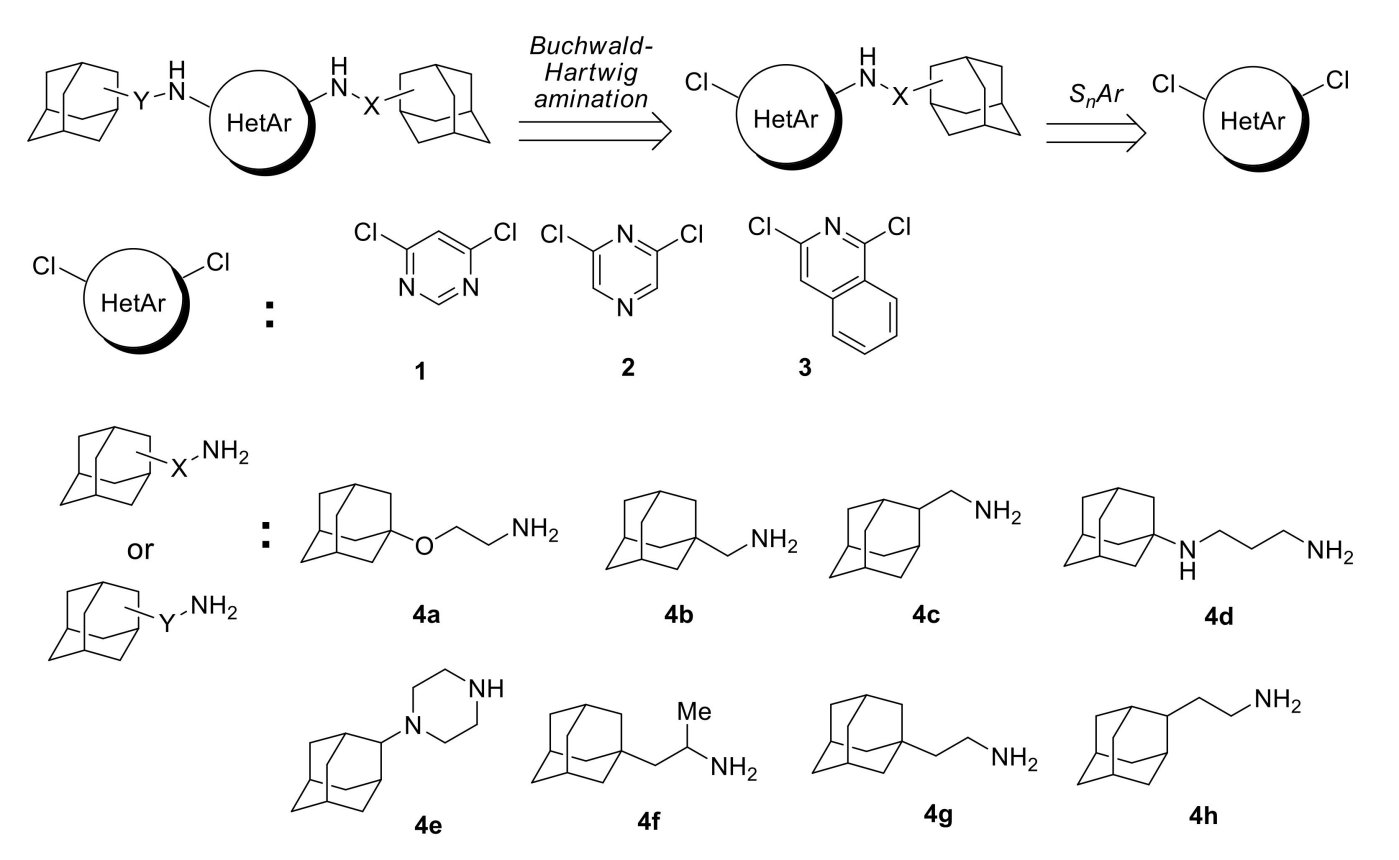
A two-step approach to adamantane-containing diamino heteroarenes studied in this work.

For the aforementioned reasons, the aim of this work is the elaboration of the approach to adamantane-containing 4,6-diaminopyrimidines, 2,6-diaminopyrazines, and 1,3-diaminoisoquinolines. Selective monoamination of the corresponding dichloroheteroarenes can be accomplished in high yields under catalyst-free conditions [31]. Non-catalytic introduction of the second amino group is seriously hindered by the electron donor effect of the first amino group. It is rational to use the Pd-catalyzed amination at the second step as it is depicted on Scheme 1. Earlier we demonstrated Pd-catalyzed diamination to be useful in one-step syntheses of symmetrical adamantane-containing diamino derivatives of pyridines [32], quinolines [33], and 1,10-phenanthrolines [34].

## 2. Results and Discussion

**Scheme 2:**
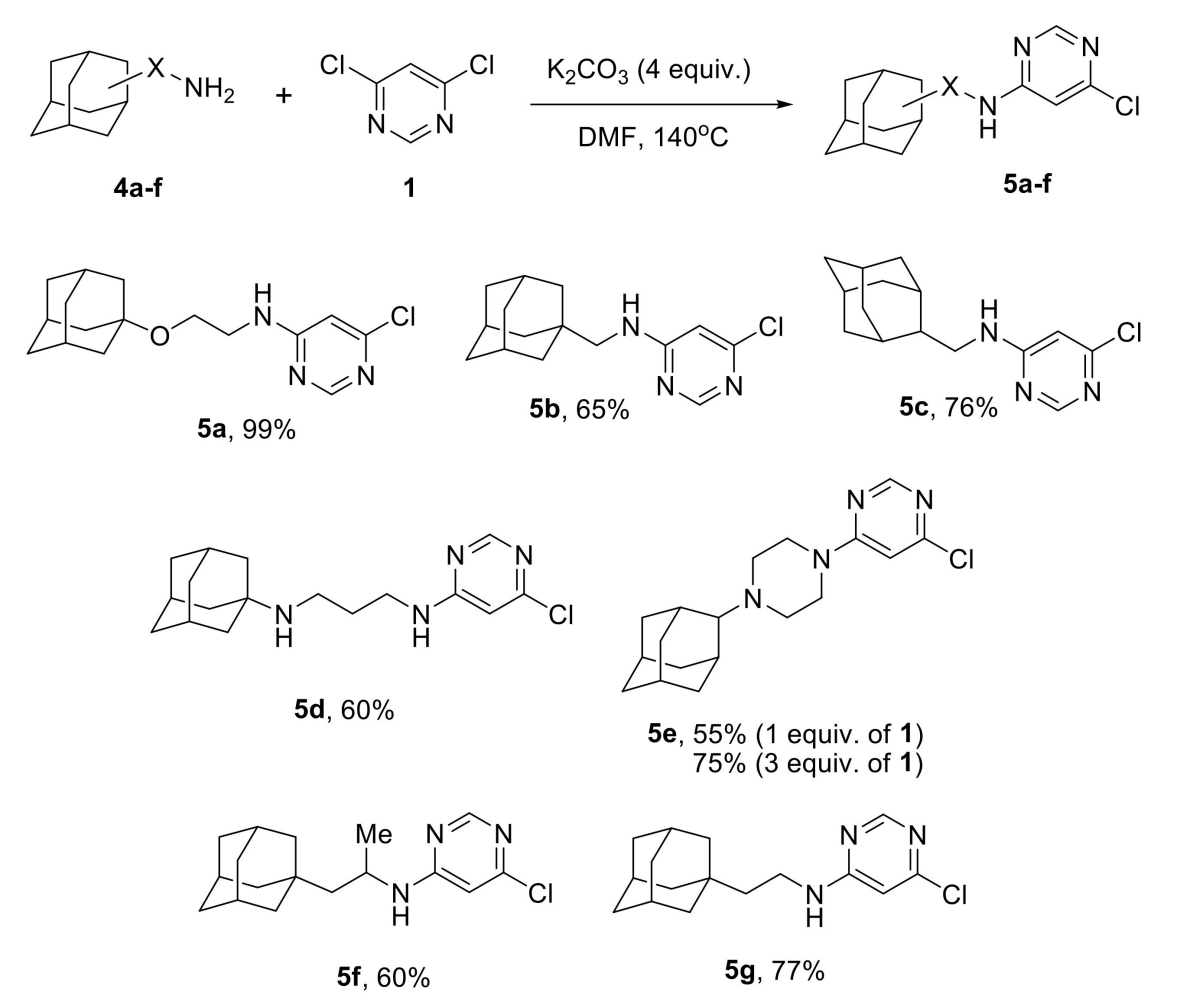
Monoamination of 4,6-dichloropyrimidine with adamantane-containing amines.

In the present study, we employed commercially available 3,6-dichloropyrimidine **1**, 2,6-dichloropyrazine **2**, and 1,3-dichloroisoquinoline **3** and a set of adamantane-containing amines differing by the substituents at the amino group (Scheme 1). At first, the catalyst-free monoamination of 4,6-dichloropyrimidine **1** was undertaken, and the reactions were conducted under earlier optimized conditions (4 equiv. K_2_CO_3_, DMF, 140 °C) (Scheme 2).

The heteroarylation of a sterically unhindered amine **4a** proceeded smoothly and resulted in almost quantitative yield of the product **5a**. Amines **4c,g** with a closer position of the adamantane core to the amino group produced the target compounds in 76 and 77% yields, respectively. An increase in the steric hindrances in **4b,f** resulted in lower yields of the amination products **5b,f** (65 and 60%). The diamine **4d** reacted selectively and not surprisingly only the primary amino group participated in the heteroarylation affording compound **5d** in 60% yield. Initially, 55% yield was obtained in the reaction with the secondary amine **5e**, but the result was improved (75%) by taking 3 equiv. 4,6-dichloropyrimidine.

**Scheme 3:**
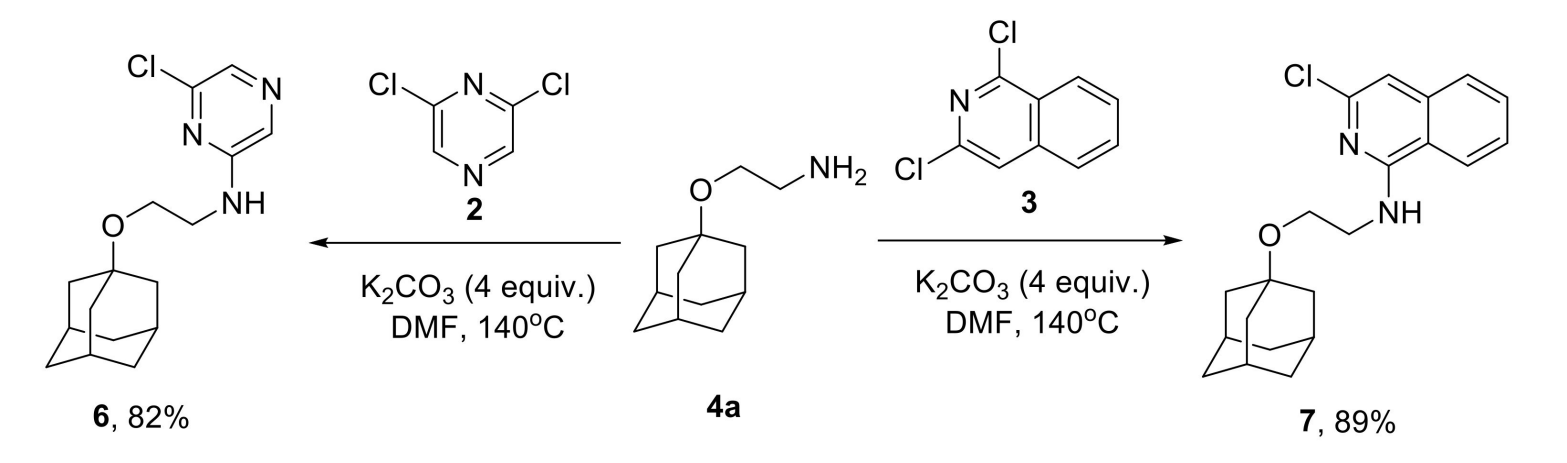
Monoamination of 2,6-dichloropyrazine and 1,3-dichloroisoquinoline.

Catalyst-free monoamination of 2,6-dichloropyrazine **2** and 1,3-dichloroisoquinoline **3** with a wide scope of adamantane-containing amines was studied earlier [31]. The known protocol [31] was employed for the synthesis of *N*-(2-(1-adamantyloxy)ethyl)-6-chloropyrazin-2-amine **6** and *N*-[2-(1-adamantyloxy)ethyl]-3-chloroisoquinolin-1-amine **7** in 82 and 89% yields (Scheme 3) for further studies of the substitution of the chlorine in these compounds.

**Scheme 4:**
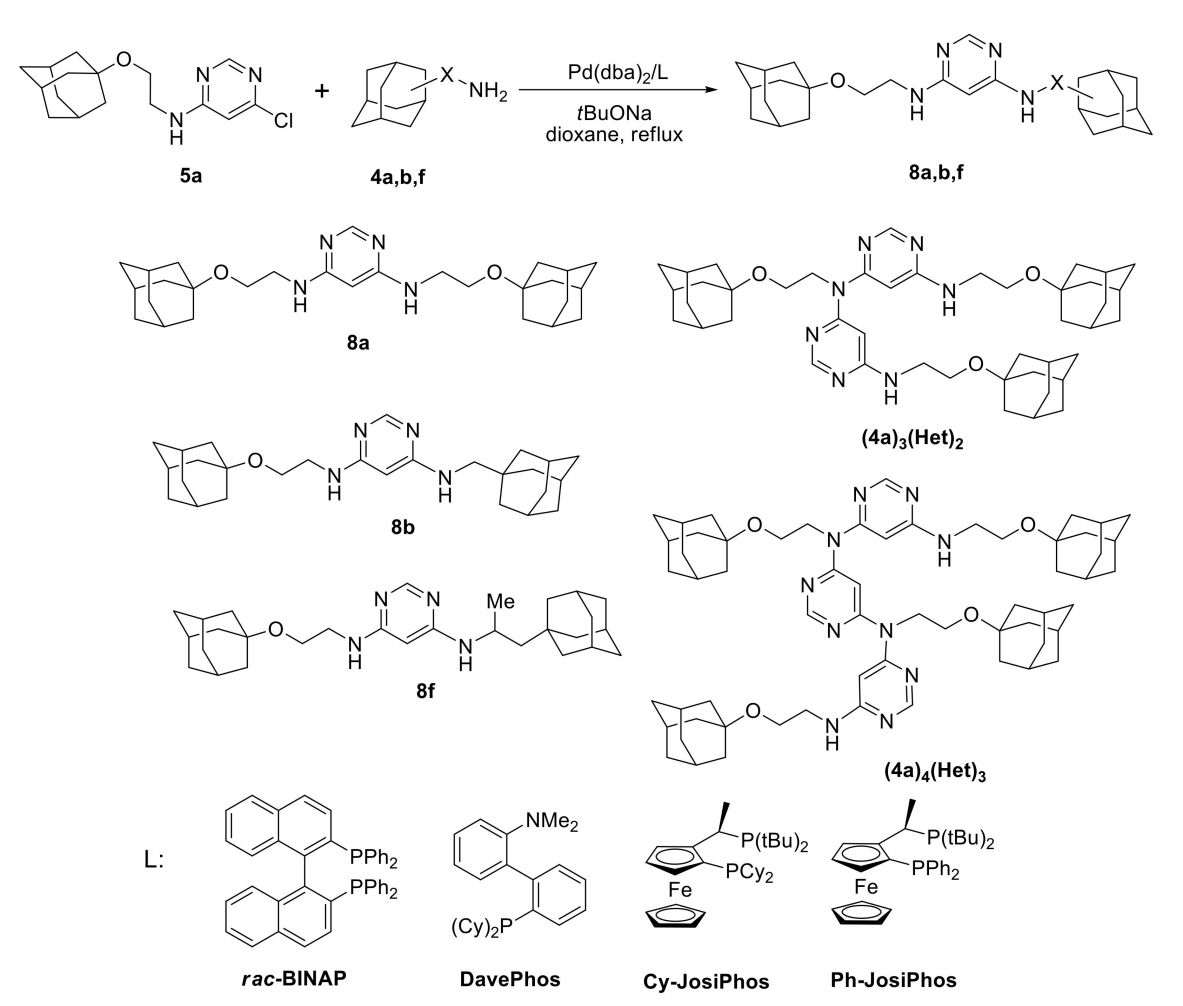
Synthesis of adamantane-containing 4,6-diaminopyrimidines.

The synthesis of adamantane-containing 4,6-diaminopyrimidines was studied using a model compound **5a** with Pd(dba)_2_ as a precatalyst (Scheme 4, Table 1). Classical phosphine ligands, like BINAP (2,2′-bis(diphenylphosphino)-1,1′-binaphthyl) and DavePhos (2-Dicyclohexylphosphino-2′-(*N,N*-dimethylamino)biphenyl) [35], were studied, as well as Ph-JosiPhos and Cy-JosiPhos (CyPF-^t^Bu), which were shown to be efficient in the amination of halosubstituted heterocycles [36,37], were also tested. Other donor phosphine ligands with bulky substituents (SPhos, XPhos, RuPhos BrettPhos) were not studied in this reaction as they earlier had been shown to be much less efficient in the heteroarylation of adamantane-containing amines with 2-bromopyridines [38]. The reaction of **5a** with 1 equiv. of amine **4a** in the presence of Pd(0)/BINAP (Table 1, entry 1) produced a complex mixture of compounds. The ^1^H NMR signals in the range of 4.0–4.5 ppm (attributed to the signals of CH_2_N(HetAr)_2_ groups) suggest the formation of various products of *N,N*-diheteroarylation including oligomeric ones. MALDI-TOF mass spectra revealed the presence of the oligomers comprising 2–3 pyrimidine units combined with 3 or 4 amine fragments which could not be separated by column chromatography (e.g., **(4a)_3_Het_2_**, [M + H]^+^ 738.48 and **(4a)_4_Het_3_**, [M + H]^+^ 1009.59, Scheme 4). *N*,*N*-diheteroarylation was observed to be typical in the amination of 2-halosubstituted 6-membered heterocycles (pyridine and quinoline) [32,33,39,40].

The use of bulky ligands (DavePhos, Ph-JosiPhos, and Cy-JosiPhos) did not lead to an increase in the product yield even in the presence of 2 equiv. amine (entries 2–4). The use of 4 equiv. amine allowed to obtain the target product in 60% yield (entry 5). The use of BINAP in this case gave the same yield, but with higher catalyst loading (entry 6). The reaction of more sterically hindered amines **4b** and **4f** with **5a** in the presence of Pd(0)/DavePhos catalyst led to the formation of the unsymmetrical products **8b** (40%) and **8f** (46%), respectively (entries 7, 8). *N,N*-diheteroarylation was also observed in all cases; however, corresponding products were always obtained as mixtures and could not be isolated in pure state.

**Scheme 5:**
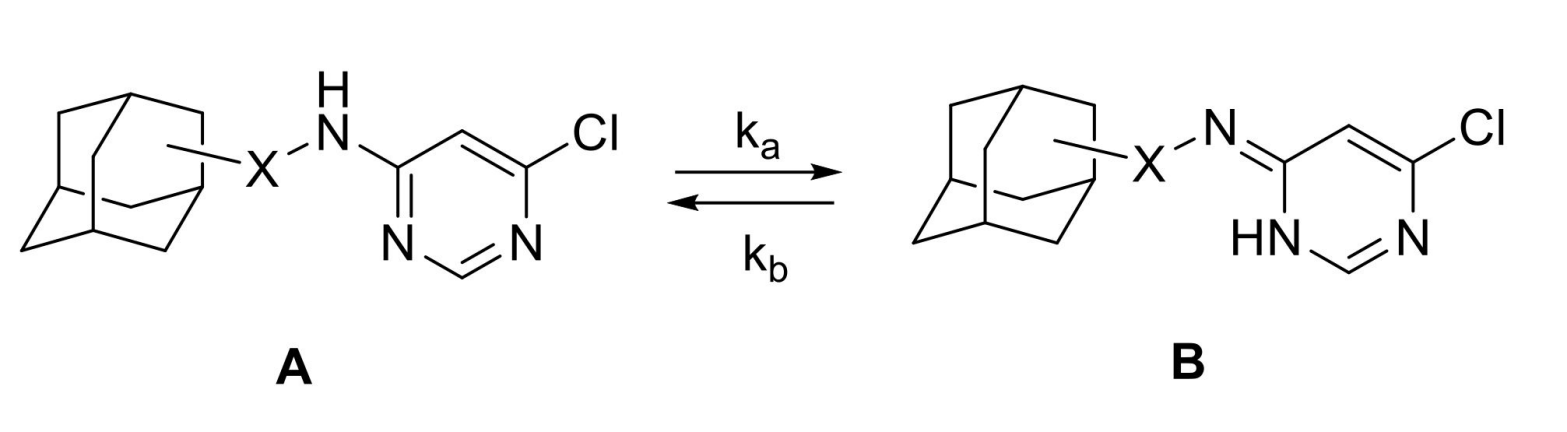
Prototropic equilibrium in 4-amino-6-chloropyrimidines.

Such propensity for the diheteroarylation can be explained by the fact that 4-amino-6-chloropyrimidines exist as two tautomers **A** and **B** in equilibrium (Scheme 5). It may increase the NH-Het proton mobility and result in a faster *N*,*N*-diheteroarylation. Tautomeric equilibria of amine-imine type are natural for 2-aminoheterocycles and have been under investigation as it is a common form of isomerism for nucleobases. This phenomenon governs the ability of the heterocycles to form hydrogen bonds [41] and participate in redox processes [42].

In the case of the pyrimidine derivatives **5,** the tautomers were observed in the ^1^H NMR spectra. Line broadening was notable for NH, aliphatic CHN protons, as well for the protons of the heterocyclic ring. To get a deeper insight in the phenomenon, the influence of the steric hindrances at the amino group on the tautomerism was studied for the compounds **5b**, **5c**, **5f,** and **5g**. NMR spectra of these compounds were registered in CDCl_3_ at different temperatures in the range 223–323 K to get activation energy characteristics presented in Table 2.

**Scheme 6:**
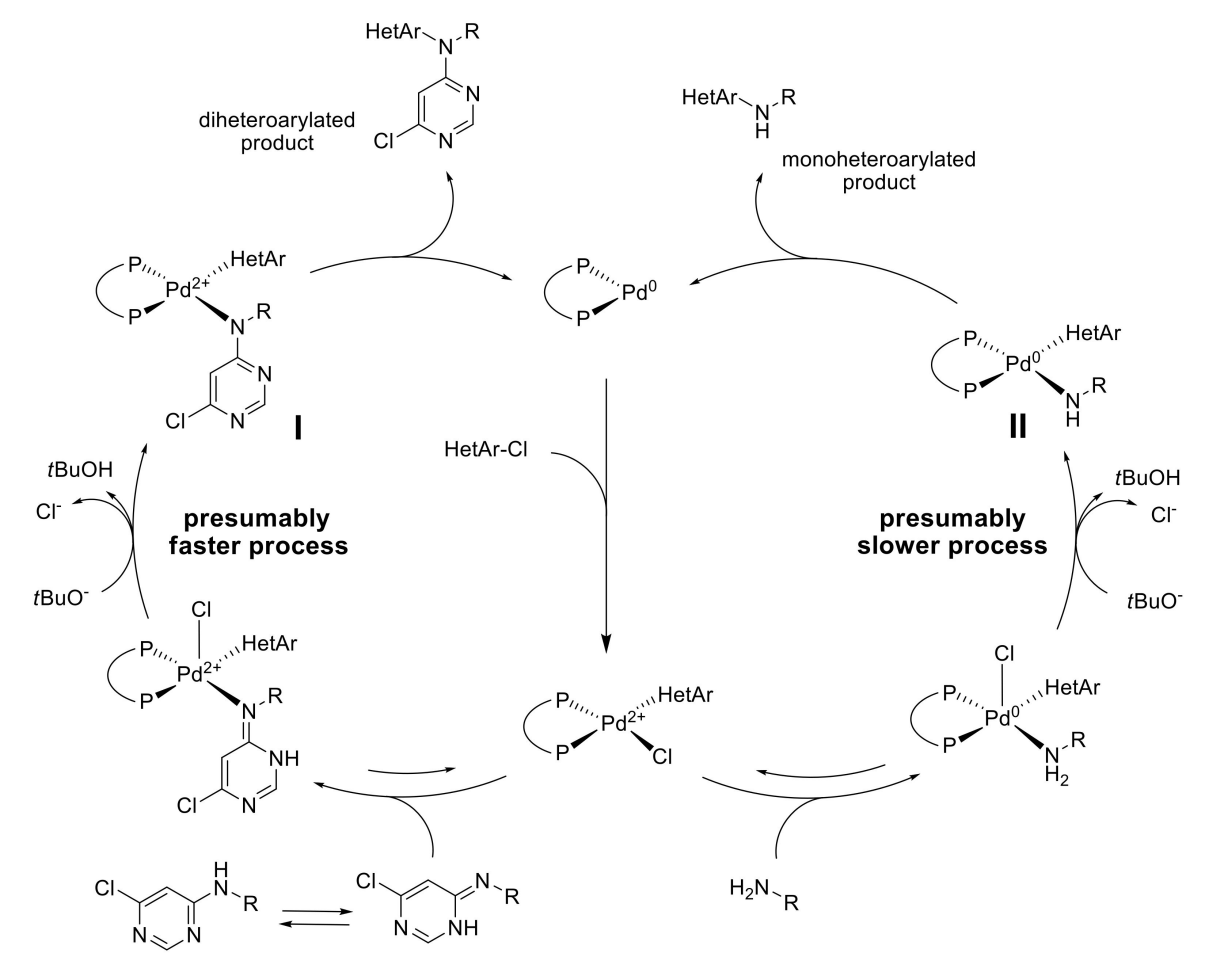
Proposed mechanism of the formation of mono- and di-heteroarylated products.

The spectra were fitted using WinDNMR software and tautomers ratio was calculated (see Supplementary Materials). Reaction rate constants sum (k = k_a_ + k_b_) and equilibrium constant (K = k_a_/k_b_ = [B]/[A]) were determined for each temperature. The activation energy characteristics (ΔH^≠^ and ΔS^≠^) were calculated from the experiments using Eyring equation and are given in Table 2. While enthalpy and entropy of the activation of the processes differ substantially, the Gibbs energy does not show significant dependence on the structure of the compounds. With elevating the temperature, an increase in the imine ratio is observed and reaches 40% at 351–325 K. This could explain an easy formation of the palladium amide **I** complex from the imine form and, consequently, the tendency of the monoarylated product to participate in the second arylation step forming the *N,N*-diaryl derivative (Scheme 6). As it is known, the deprotonation of the palladium amine complex with its transformation into the amide complex is a rate-determining step of the catalytic cycle, and, in the present case, formation of the amide complex **I** is faster than amide complex **II** because of equilibrium between **A** and **B** forms.

**Scheme 7:**
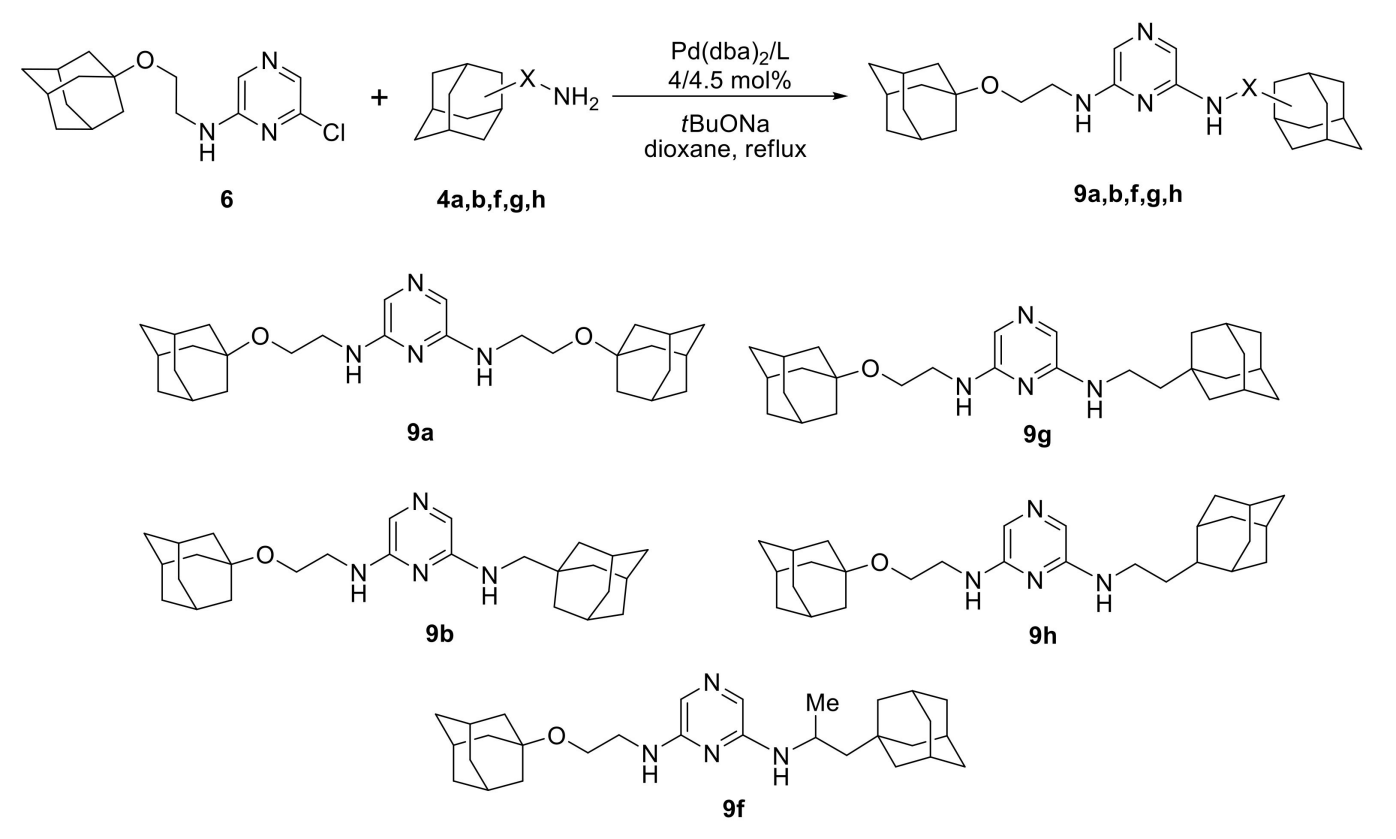
Synthesis of adamantane-containing 2,6-diaminopyrazines.

The amination of 2-amino-6-chloropyrazine **6** (Scheme 7) using 1 equiv. of **4a** also led to a complex mixture of oligomers (Table 3, entries 1, 2), preventing the isolation of the target compound **9a** in a pure state. The application of 2 equiv. amine in the presence of Pd(0)/Cy-JosiPhos catalytic system afforded **9a** in 30% yield (entry 3) and the use of 4 equiv. amine with Ph-JosiPhos allowed the isolation of **9a** in 90% yield (entry 4). Under analogous conditions, **9b**, **9g,** and **9h** were synthesized in moderate yields 36–48% (entries 5, 7, 8). Indeed, the target products were obtained in much better yields in the reaction mixtures, but the losses during the separation from the oligomeric products badly decreased the isolated yields. Compound **9f** could not be isolated in a pure state due to such problem; its yield was estimated as 47% by ^1^H NMR spectrum (entry 6).

**Scheme 8:**
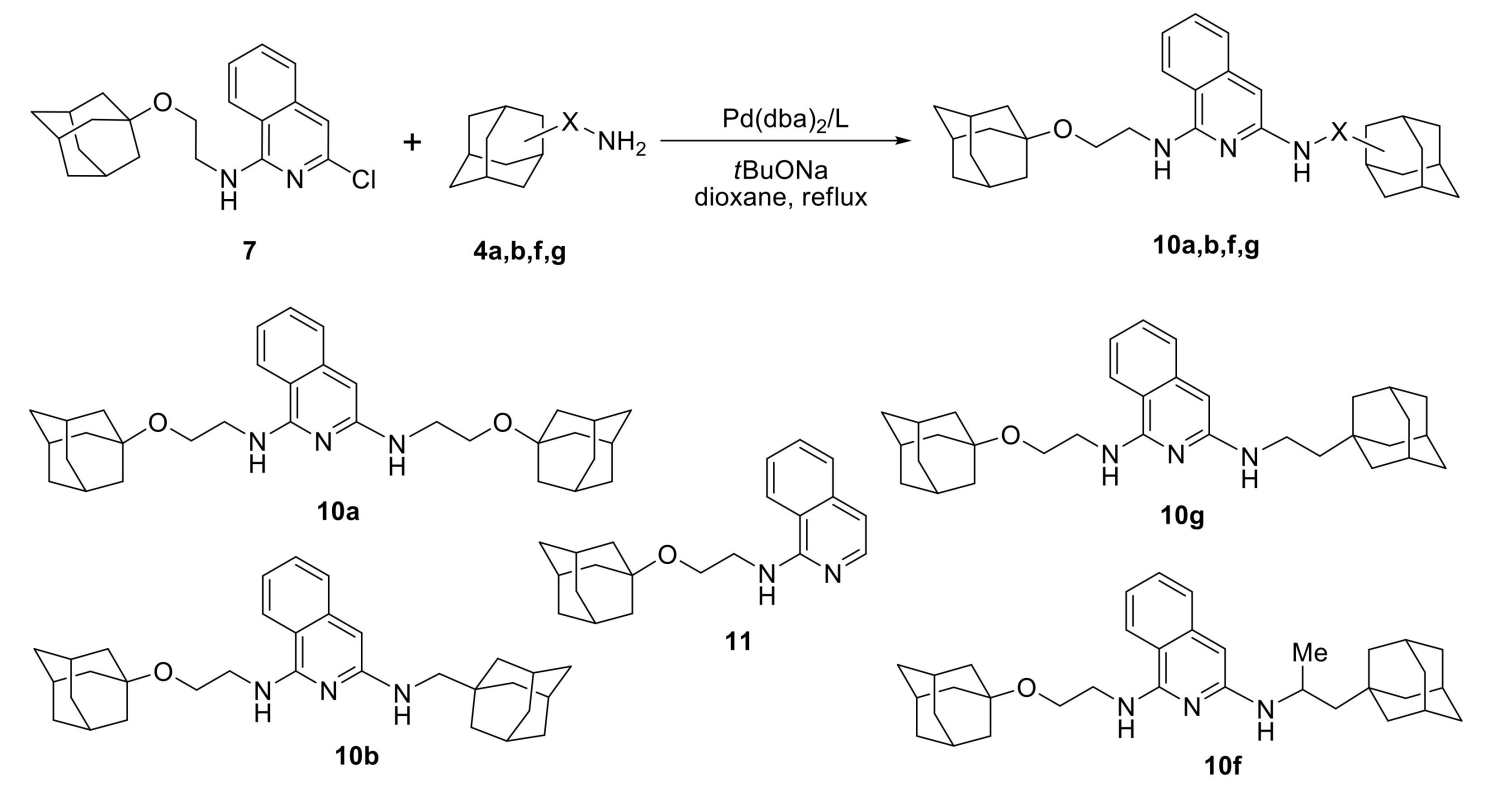
Synthesis of adamantane-containing 1,3-diaminoisoquinolines.

The amination of 2-amino-3-chloroisoquinoline **7** (Scheme 8) is also complicated by the formation of oligomers; however, in this case, it is somewhat less prominent, presumably due to more steric hindrances at the NH group. The application of 2 equiv. amine in the presence of Pd(0)/Cy-JosiPhos and Pd(0)/DavePhos catalytic systems gave only a mixture of oligomers (Table 4, entries 1,2). In the first case, the formation of the product **11** of C-Cl bond reduction was observed (entry 1). The use of 4 equiv. amine in the presence of Pd(0)/DavePhos afforded the desired compound **10a** in 77% yield (entry 3). The test of the BINAP ligand, even at greater catalyst loading, did not lead to an increase in the product yield (entry 4). Under optimized conditions, 1,3-diaminoisoquinolines **10b**, **10f** and **10g** were obtained in 43–67% yields (entries 5–7).

## 3. Materials and Methods

NMR spectra were registered using a Bruker Avance 400 spectrometer (Bruker Daltonics, Germany), MALDI-TOF spectra were obtained with a Bruker Autoflex II spectrometer (Bruker Daltonics, Germany) using 1,8,9-trihydroxyanthracene as a matrix and polyethylene glycols (PEGs) as internal standards. Dichloropyrimidine, dichloropyrazine, dichloroisoquinoline, BINAP, DavePhos and JosiPhos ligands, sodium *tert*-butoxide, and potassium carbonate were purchased from Aldrich (Daltonics, Germany) and Acros (Morris Plains, NJ, USA) and used without further purification. Amines **4a** and **4b** were prepared according to reported procedures of References [43,44], respectively. Amines **4c** and **4e,g,h**, were obtained according to a method described in Reference [45], amines **4d** and **4f** were obtained according to methods described in References [39,46]. Pd(dba)_2_ was synthesized from PdCl_2_ according to a known procedure [47]. Dioxane was distilled over NaOH followed by the distillation over sodium under argon. Dimethylformamide was distilled over sodium hydride under reduced pressure. Dichloromethane and methanol were used freshly distilled.

### 3.1. N-(Heteroaryl)-Substituted Adamantane-Containing Amines 5–7 (General Procedure)

A corresponding chlorosubstituted heteroarene (0.5–2 mmol), finely powdered anhydrous K_2_CO_3_ (1.25–8 mmol), DMF (1–4 mL), and a corresponding adamantane-containing amine **4** (0.5–2 mmol) were placed into a glass vial equipped with a magnetic stirrer and screw tap, and the reaction mixture was stirred for 24 h at 140 °C. On the reaction completion, the mixture was cooled to room temperature, dichloromethane (5 mL) was added, an inorganic precipitate was filtered off and additionally washed with dichloromethane (5 mL), the combined organic filtrates were concentrated in vacuo, and the residues were analyzed by NMR spectroscopy. The products were purified by chromatography on silica gel, using the following sequence of eluents: hexanes—CH_2_Cl_2_ (4:1–1:4), CH_2_Cl_2_, CH_2_Cl_2_—MeOH (200:1–50:1).

*N-[2-(1-Adamantyloxy)ethyl]-6-chloropyrimidin-4-amine* (**5a**). Obtained from dichloropyrimidine **1** (298 mg), amine **4a** (390 mg) in the presence of K_2_CO_3_ (1.12 g) in 4 mL of DMF. Yield 608 mg (99%), white amorphous powder, m.p. 136–137 °C. ^1^H-NMR (CDCl_3_) δ 1.55–1.64 (m, 6H, Ad), 1.69–1.70 (m, 6H, Ad), 2.10 (s, 3H, Ad), 3.41 (br. s, 2H, CH_2_(NH)), 3.55 (br.t, 2H, ^3^*J_obs_* = 4.9 Hz, CH2(O)), 5.73 (br. s., 1H, NH), 6.35 (s, 1H, H5), 8.29 (s, 1H, H2). ^13^C-NMR(CDCl_3_) δ 30.2 (3C, Ad), 36.1 (3C, Ad), 41.2 (3C, Ad), 41.6 (1C, CH_2_(NH)), 57.9 (1C, CH_2_(O)), 72.4 (1C, C_quat_(O)), 103.8 (1C, C5), 158.1 (2C, C2, C4), 163.1 (1C, C6). HRMS (MALDI-TOF): *m*/*z* [M + H]^+^ calcd for C_16_H_23_ClN_3_O: 308.1524; observed: 308.1481.

*N-[(1-Adamantyl)methyl]-6-chloropyrimidin-4-amine* (**5b**). Obtained from dichloropyrimidine (75 mg), amine **5b** (83 mg) in the presence of K_2_CO_3_ (276 mg) in 1 mL of DMF. Yield 90 mg (65%), yellowish viscous oil. ^1^H-NMR (CDCl_3_) δ 1.44–1.75 (m, 12H, Ad), 1.99 (s, 3H, Ad), 2.92 (br. s., 2H, CH_2_(NH)), 5.84 (br. s., 1H, NH), 6.33 (s, 1H, H5), 8.28 (s, 1H, H2). ^13^C-NMR(CDCl_3_) δ 28.1 (3C, Ad), 34.1 (1C, C_quat_), 36.8 (3C, Ad), 40.3 (3C, Ad), 53.5 (1C, CH_2_(NH)), 100.3 (1C, C5), 158.2 (2C, C2, C4), 159.1 (1C, C4), 163.9 (1C, C6). HRMS (MALDI-TOF): *m*/*z* [M + H]^+^ calcd for C_15_H_21_ClN_3_: 278.1419; observed: 278.1399.

*N-[(2-Adamantyl)methyl]-6-chloropyrimidin-4-amine* (**5c**). Obtained from K_2_CO_3_ (276 mg) in 1 mL of DMF. Yield 105 mg (76%), yellowish viscous oil. ^1^H-NMR (CDCl_3_) δ 1.55 (d, 2H, ^3^*J* = 11.8 Hz, Ad), 1.67–1.71 (m, 4H, Ad), 1.77–1.90 (m, 9H, Ad), 3.35 (br. s, 2H, CH_2_(NH)), 5.84 (br. s., 1H, NH), 6.33 (s, 1H, H5), 8.28 (s, 1H, H2). ^13^C-NMR(CDCl_3_) *δ* 27.9 (1C, Ad), 28.0 (1C, Ad), 30.0 (2C, Ad), 31.6 (2C, Ad), 37.9 (1C, Ad), 38.6 (2C, Ad), 43.9 (2C, CH_2_(NH), Ad), 100.4 (1C, C5), 153.5 (2C, C2, C4), 163.4 (1C, C6). HRMS (MALDI-TOF): *m*/*z* [M + H]^+^ calcd for C_15_H_21_ClN_3_: 278.1419; observed: 278.1463.

*N-(6-chloropyrimididin-4-yl)-N′-(1-Adamantyl)propan-1,3-diamine* (**5d**). Obtained from dichloropyrimidine (75 mg), amine **4d** (104 mg) in the presence of K_2_CO_3_ (276 mg) in 1 mL of DMF. Yield 96 mg (60%), brownish viscous oil. ^1^H-NMR (CDCl_3_) δ 1.52–1.66 (m, 14H, CH_2_, Ad), 2.18 (s, 3H, Ad), 3.20–3.39 (m, 5H, NH, CH_2_(NH)), 6.51 (s, 1H, H5), 8.34 (s, 1H, H2). ^13^C-NMR(CDCl_3_) δ 29.3 (3C, Ad), 29.8 (1C, CH_2_), 35.8 (3C, Ad), 36.7 (1C, CH_2_(NH)), 38.7 (1C, CH_2_(NH)), 42.2 (3C, Ad), 56.7 (1C, C_quat_), 104.9 (1C, C2), 156.8 (1C, C4), 161.9 (1C, C6), 162.2 (1C, C5). HRMS (MALDI-TOF): *m*/*z* [M + H]^+^ calcd for C_17_H_26_ClN_4_: 321.1841; observed: 321.1834.

*N-[4-(2-Adamantyl)piperazin-1-yl]-6-chloropyrimidine* (**5e**). Obtained from dichloropyrimidine (224 mg), amine **4e** (113 mg) in the presence of K_2_CO_3_ (276 mg) in 1 mL of DMF. Yield 125 mg (75%), brownish viscous oil. ^1^H-NMR (CDCl_3_) δ 1.43 (d, 2H, ^3^*J* = 12.1 Hz, Ad), 1.55–1.62 (m, 4H, Ad), 1.71–1.79 (m, 4H, Ad), 2.06 (s, 5H, Ad), 2.39–2.43 (m, 4H, CH_2_(N)), 3.62 (br. s, 4H, CH_2_(N)), 6.47 (s, 1H, H5), 8.35 (s, 1H, H2). ^13^C-NMR(CDCl_3_) δ 27.0 (1C, Ad), 27.3 (1C, Ad), 28.8 (2C, Ad), 31.1 (2C, Ad), 36.9 (2C, Ad), 37.5 (1C, Ad), 44.2 (2C, CH_2_(N)), 48.8 (2C, CH_2_(N)), 67.0 (1C, Ad(N)), 101.0 (1C, C2), 157.8 (1C, C5), 159.7 (1C, C4), 162.0 (1C, C6). HRMS (MALDI-TOF): *m*/*z* [M + H]^+^ calcd for C_18_H_26_ClN_4_: 333.1841; observed: 333.1866.

*N-[2-(1-Adamantyl)propan-2-yl]-6-chloropyrimidin-4-amine* (**5f**). Obtained from dichloropyrimidine (75 mg), amine **4f** (98 mg) in the presence of K_2_CO_3_ (276 mg) in 1 mL of DMF. Yield 92 mg (60%), yellowish viscous oil. ^1^H-NMR (CDCl_3_) δ 1.15 (d, 3H, ^3^*J* = 6.3 Hz, CH_3_), 1.26–1.29 (m, 2H, CH_2_(Ad)), 1.43–1.50 (m, 6H, Ad), 1.54–1.66 (m, 6H, Ad), 1.89 (s, 3H, Ad), 3.68 (br. s, 1H, CHN), 5.51 (br. s, 1H, NH), 6.27 (s, 1H, H5), 8.28 (s, 1H, H2). ^13^C-NMR(CDCl_3_) δ 23.0 (1C, CH_3_), 28.4 (3C, Ad), 32.4 (1C, C_quat_), 36.8 (3C, Ad), 42.8 (4C, Ad, CH_2_(Ad)), 52.0 (1C, CH_2_(NH)), 100.1 (1C, C5), 158.4 (2C, C2, C4), 161.9 (1C, C6). HRMS (MALDI-TOF): *m*/*z* [M + H]^+^ calcd for C_17_H_25_ClN_3_: 306.1732; observed: 306.1757.

*N-[2-(1-Adamantyl)ethyl]-6-chloropyrimidin-4-amine* (**5g**). Obtained from dichloropyrimidine (75 mg), amine **4g** (90 mg) in the presence of K_2_CO_3_ (276 mg) in 1 mL of DMF. Yield 112 mg (77%), yellowish glassy compound. ^1^H-NMR (CDCl_3_) δ 1.35–1.38 (m, 2H, CH_2_(Ad)), 1.48–1.55 (m, 6H, Ad), 1.57–1.73 (m, 6H, Ad), 1.95 (s, 3H, Ad), 3.22 (br. s, 2H, CH_2_N), 5.62 (br. s, NH), 6.29 (s, 1H, H5), 8.29 (s, 1H, H2). ^13^C-NMR(CDCl_3_) δ 28.6 (3C, Ad), 32.0 (1C, CH_2_(Ad)), 36.7 (1C, C_quat_ (Ad)), 37.0 (3C, Ad), 42.5 (3C, Ad), 43.5 (1C, Ad, CH_2_(NH)), 103.3 (1C, C5), 155.4 (1C, C4), 158.4 (1C, C2), 163.4 (1C, C6). HRMS (MALDI-TOF): *m*/*z* [M + H]^+^ calcd for C_16_H_23_ClN_3_: 292.1575; observed: 292.1540.

Syntheses of *N*-(2-(1-Adamantyloxy)ethyl)-6-chloropyrazin-2-amine (**6**) and *N*-[2-(1-Adamantyloxy)ethyl]-3-chloroisoquinolin-1-amine (**7**) were previously reported in ref. [31].

### 3.2. Palladium-Catalyzed Amination of Chloroheterocycles—General Procedure

A two-neck flask equipped with a condenser and a magnetic stirrer, flushed with dry argon, was charged with corresponding chloroheterocycle (0.2 mmol), Pd(dba)_2_ (2.5–12 mg, 2–8 mol%), phosphine ligand (2.5–9 mol%), and absolute dioxane (2 mL). The mixture was stirred for 2–3 min, then corresponding amine **4** (0.2–0.8 mmol) and *t*BuONa (0.375 mmol) were added, and the reaction mixture was refluxed for 24 h. After cooling it down to ambient temperature, the reaction mixture was diluted with CH_2_Cl_2_, the solution filtered and evaporated in vacuo, and the residue was chromatographed on silica gel using a sequence of eluents: hexanes, hexanes–CH_2_Cl_2_ 2:1–1:1, CH_2_Cl_2_, CH_2_Cl_2_/MeOH 200:1–3:1.

*N,N′-bis[2-(1-adamantyloxy)ethyl]pyrimidine-4,6-diamine* (**8a**). Obtained from chloroheterocycle **5a** (61.5 mg), amine **4a** (156 mg) in the presence of Pd(dba)_2_ (5 mg), DavePhos (3.5 mg) and *t*BuONa (29 mg). Eluent CH_2_Cl_2_/MeOH 50:1. Yield 57 mg (61%), yellowish viscous oil. ^1^H-NMR (CDCl_3_) δ 1.55–1.64 (m, 12H, Ad), 1.72 (s, 12H, Ad), 2.14 (s, 6H, Ad), 3.32–3.34 (m, 4H, CH_2_(NH))), 3.57 (t, 4H, ^3^*J* = 5.1 Hz, CH_2_(O)), 5.14 (br. s, 2H, NH), 5.29 (s, 1H, H5), 8.07 (s, 1H, H2). ^13^C-NMR(CDCl_3_) δ 30.4 (6C, Ad), 36.3 (6C, Ad), 41.5 (6C, Ad), 41.5 (2C, CH_2_(NH)), 58.2 (2C, CH_2_(O)), 72.5 (2C, C_quat_(O)), 80.2 (1C, C5), 157.5 (1C, C2), 162.7 (2C, C4, C6). HRMS (MALDI-TOF): *m*/*z* [M + H]^+^ calcd for C_28_H_43_N_4_O_2_: 467.3381; observed: 467.3344.

*N-[2-(1-adamantyloxy)ethyl]-N′-[(1-adamantyl)methyl]pyrimidine-4,6-diamine* (**8b**). Obtained from chloroheterocycle **5a** (61.5 mg), amine **4b** (132 mg) in the presence of Pd(dba)_2_ (5 mg), DavePhos (3.5 mg) and *t*BuONa (29 mg). Eluent CH_2_Cl_2_/MeOH 50:1. Yield 35 mg (40%), yellowish viscous oil. ^1^H-NMR (CDCl_3_) δ 1.52–1.71 (m, 24H, Ad), 1.97 (s, 3H, Ad), 2.12 (s, 3H, Ad), 2.83 (d, 2H, ^3^*J* = 5.9 Hz, CH_2_(NH)), 3.35 (q, 2H, ^3^*J* = 5.1 Hz, CH_2_(NH)), 3.57 (t, 2H, ^3^*J* = 5.9 Hz, CH_2_(O)), 5.20 (br. s, NH), 5.27 (s, 1H, H5), 8.00 (s, 1H, H2). ^13^C-NMR(CDCl_3_) δ 28.2 (3C, Ad), 30.4 (3C, Ad), 36.3 (3C, Ad), 36.8 (3C, Ad), 40.4 (3C, Ad), 41.5 (3C, Ad), 42.0 (1C, CH_2_(NH)), 53.6 (1C, CH_2_(NH)), 58.2 (1C, CH_2_(O)), 72.5 (1C, C_quat_(O)), 79.3 (1C, C5), 156.6 (1C, C2), 162.6 (2C, C4, C6). HRMS (MALDI-TOF): *m*/*z* [M + H]^+^ calcd for C_27_H_40_N_4_O: 437.3275; observed: 437.3255.

*N-[2-(1-adamantyloxy)ethyl]-N′-[2-(1-adamantyl)propan]pyrimidine-4,6-diamine* (**8f**). Obtained from chloroheterocycle **5a** (61.5 mg), amine **4f** (155 mg) in the presence of Pd(dba)_2_ (5 mg), DavePhos (3.5 mg) and *t*BuONa (29 mg). Eluent CH_2_Cl_2_/MeOH 50:1. Yield 43 mg (46%), yellowish viscous oil.^1^H-NMR (CDCl_3_) δ 1.14 (d, 3H, ^3^*J* = 6.3 Hz, CH_3_), 1.49–1.51 (m, 6H, Ad, CH_2_), 1.55–1.67 (m, 14H, Ad, CH_2_), 1.70–1.73 (m, 6H, Ad), 1.90 (s, 3H, Ad), 2.12 (s, 3H, Ad), 3.32–3.36 (q, 2H, ^3^*J* = 5.3 Hz, CH_2_(NH)), 3.56–3.60 (m, 3H, CH(NH), CH_2_(O)), 5.23 (s, 1H, H5), 8.04 (s, 1H, H2), NH protons were not unambiguously attributed.^13^C-NMR(CDCl_3_) δ 23.2 (1C, CH_3_), 28.2 (3C, Ad), 30.4 (3C, Ad), 32.4 (1C, C_quat_(Ad)), 36.3 (3C, Ad), 36.9 (3C, Ad), 41.5 (3C, Ad), 42.0 (1C, CH_2_(Ad)), 42.6 (1C, CH_2_(NH)), 42.9 (3C, Ad), 52.3 (1C, CH_2_(NH)), 58.2 (1C, CH_2_(O)), 72.4 (1C, C_quat_(O)), 79.6 (1C, C5), 157.7 (1C, C2), 161.3 (1C, C4), 162.8 (1C, C6). HRMS (MALDI-TOF): *m*/*z* [M + H]^+^ calcd for C_29_H_44_N_4_O: 465.3588; observed: 465.3603.

*N,N′-bis[2-(1-adamantyloxy)ethyl]pyrazine-2,6-diamine*(**9a**). Obtained from chloroheterocycle **6** (61.5 mg), amine **4a** (156 mg) in the presence of Pd(dba)_2_ (5 mg), Ph-JosiPhos (5 mg) and *t*BuONa (29 mg). Eluent CH_2_Cl_2_/MeOH 50:1. Yield 84 mg (90%), yellowish viscous oil. ^1^H-NMR (CDCl_3_) δ 1.56–1.65 (m, 12H, Ad), 1.72 (s, 12H, Ad), 2.13 (s, 6H, Ad), 3.39–3.43 (m, 4H, CH_2_(NH)), 3.56–3.59 (m, 4H, CH_2_(O)), 4.68 (s, 2H, NH), 7.17 (s, 2H, H3, H5). ^13^C-NMR(CDCl_3_) δ 30.5 (6C, Ad), 36.4 (6C, Ad), 41.6 (6C, Ad), 41.9 (2C, CH_2_(NH)), 58.5 (2C, CH_2_(O)), 72.4 (2C, C_quat_(O)), 118.4 (2C, C3, C5), 153.4 (2C, C2, C6). HRMS (MALDI-TOF): *m*/*z* [M + H]^+^ calcd for C_28_H_43_N_4_O_2_: 467.3381; observed: 467.3419.

*N-[2-(1-adamantyloxy)ethyl]-N′-[(1-adamantyl)methyl]pyrazine-2,6-diamine* (**9b**). Obtained from chloroheterocycle **6** (61.5 mg), amine **4b** (132 mg) in the presence of Pd(dba)_2_ (5 mg), Ph-JosiPhos (5 mg) and *t*BuONa (29 mg). Eluent CH_2_Cl_2_/MeOH 100:1. Yield 37 mg (42%), yellowish viscous oil. ^1^H-NMR (CDCl_3_) δ 1.52–1.72 (m, 24H, Ad), 1.96 (s, 3H, Ad), 2.12 (s, 3H, Ad), 2.95 (d, 2H, ^3^*J* = 6.7 Hz, CH_2_(NH)), 3.39 (q, 2H, ^3^*J* = 5.5 Hz, CH_2_(NH)), 3.57 (t, 2H, ^3^*J* = 5.5 Hz, CH_2_(O)), 4.36 (t, 1H, ^3^*J* = 5.9 Hz, NH), 4.66 (t, 1H, ^3^*J* = 5.5 Hz, NH), 7.12 (s, 1H, H5), 7.15 (s, 1H, H3). ^13^C-NMR(CDCl_3_) δ 28.2 (3C, Ad), 30.4 (3C, Ad), 34.1 (1C, C_quat_), 36.3 (3C, Ad), 36.9 (3C, Ad), 40.4 (3C, Ad), 41.5 (3C, Ad), 41.9 (1C, CH_2_(NH)), 53.2 (1C, CH_2_(Ad)), 58.4 (1C, CH_2_(O)), 72.4 (1C, C_quat_(O)), 115.4 (1C, C3), 115.7 (1C, C5), 153.6 (1C, C2), 154.3 (1C, C6). HRMS (MALDI-TOF): *m*/*z* [M + H]^+^ calcd for C_27_H_41_N_4_O: 437.3275; observed: 437.3261.

*N-[2-(1-adamantyloxy)ethyl]-N′-[2-(1-adamantyl)ethan]pyrazine-2,6-diamine* (**9g**). Obtained from chloroheterocycle **6** (61.5 mg), amine **4g** (143 mg) in the presence of Pd(dba)_2_ (5 mg), Ph-JosiPhos (5 mg) and *t*BuONa (29 mg). Eluent CH_2_Cl_2_/MeOH 50:1. Yield 43 mg (48%), yellowish viscous oil. ^1^H-NMR (CDCl_3_) δ 1.50–1.84 (m, 26H, Ad, CH_2_(Ad)), 2.13 (s, 6H, Ad), 3.20–3.25 (m, 2H, CH_2_(NH)), 3.39 (q, 2H, ^3^*J* = 5.4 Hz, CH_2_(NH)), 3.56 (t, 2H, ^3^*J* = 5.3 Hz, CH_2_(O)), 4.30 (t, 1H, ^3^*J* = 5.4, NH), 4.68 (t, 1H, ^3^*J* = 5.6 Hz, NH), 7.14 (s, 1H, H3), 7.17 (s, 1H, H5). ^13^C-NMR(CDCl_3_) δ 27.9 (1C, CH_2_(Ad)), 30.4 (3C, Ad), 31.5 (3C, Ad), 31.6 (3C, Ad), 31.8 (1C, C_quat_), 36.4 (3C, Ad), 38.2 (1C, CH_2_(NH)), 39.0 (3C, Ad), 41.5 (3C, Ad), 41.8 (1C, CH_2_(NH)), 58.5 (1C, CH_2_(O)), 72.3 (1C, C_quat_(O)), 117.6 (1C, C3), 118.6 (1C, C5), 153.4 (2C, C2, C6). HRMS (MALDI-TOF): *m*/*z* [M + H]^+^ calcd for C_28_H_43_N_4_O: 451.3431; observed: 451.3454.

*N-[2-(1-adamantyloxy)ethyl]-N′-[2-(2-adamantyl)ethan]pyrazine-2,6-diamine* (**9h**). Obtained from chloroheterocycle **6** (61.5 mg), amine **4h** (143 mg) in the presence of Pd(dba)_2_ (5 mg), Ph-JosiPhos (5 mg) and *t*BuONa (29 mg). Eluent CH_2_Cl_2_/MeOH 50:1. Yield 32 mg (36%), yellowish viscous oil. ^1^H-NMR (CDCl_3_) δ 1.51–1.94 (m, 29H, Ad, CH_2_(Ad)), 2.14 (s, 3H, Ad), 3.22–3.26 (m, 2H, CH_2_(NH)), 3.37–3.42 (m, 2H, CH_2_(NH)), 3.57 (t, 2H, ^3^*J* = 5.4 Hz, CH_2_(O)), 4.17 (s, 1H, NH), 4.69 (s, 1H, NH), 7.13 (s, 1H, H3), 7.18 (s, 1H, H5). ^13^C-NMR(CDCl_3_) δ 28.5 (1C, Ad), 28.6 (1C, Ad), 30.4 (3C, Ad), 36.3 (1C, Ad), 36.4 (3C, Ad), 36.9 (2C, Ad), 37.0 (2C, Ad), 41.5 (3C, Ad), 41.9 (1C, CH_2_(Ad)), 42.0 (1C, CH_2_(NH)), 42.4 (1C, Ad), 42.5 (2C, Ad), 44.0 (1C, CH_2_(NH)), 58.5 (1C, CH_2_(O)), 72.3 (1C, C_quat_(O)), 117.6 (1C, C3), 118.7 (1C, C5), 153.4 (1C, C6), 153.5 (1C, C2). HRMS (MALDI-TOF): *m*/*z* [M + H]^+^ calcd for C_28_H_43_N_4_O: 451.3431; observed: 451.3430.

*N,N′-bis[2-(1-adamantyloxy)ethyl]isoquinoline-1,3-diamine* (**10a**). Obtained from chloroheterocycle **7** (71.5 mg), amine **4a** (156 mg) in the presence of Pd(dba)_2_ (5 mg), DavePhos (3.5 mg) and *t*BuONa (29 mg). Eluent CH_2_Cl_2_/MeOH 50:1. Yield 79 mg (77%), yellowish viscous oil. ^1^H-NMR (CDCl_3_) δ 1.57–1.65 (m, 12H, Ad), 1.76 (s, 12H, Ad), 2.15 (s, 6H, Ad), 3.37 (t, 2H, ^3^*J* = 5.6 Hz, CH_2_(NH)), 3.63–3.67 (m, 4H, CH_2_(NH), CH_2_(O)), 3.70–3.72 (m, 2H, CH_2_(O)), 4.62 (br. s., 1H, NH), 5.65 (s, 1H, NH), 5.87 (s, 1H, H4), 7.04 (ddd, 1H, ^3^*J* = 8.2, ^3^*J* = 6.4, ^4^*J* = 1.7 Hz, H7), 7.33–7.39 (m, 2H, H5, H6), 7.54 (d, 1H, ^3^*J* = 8.3 Hz, H8). ^13^C-NMR(CDCl_3_) δ 30.4 (6C, Ad), 36.3 (6C, Ad), 41.5 (3C, Ad), 41.6 (3C, Ad), 41.7 (1C, CH_2_(NH)), 43.3 (1C, CH_2_(NH)), 58.6 (1C, CH_2_(O)), 58.9 (1C, CH_2_(O)), 72.2 (2C, C_quat_(O)), 85.7 (1C, C4), 113.1 (1C, C8a), 120.6 (1C, C5), 121.6 (1C, C8), 125.1 (1C, C7), 129.5 (1C, C6), 140.5 (1C, C4a), 154.3 (1C, C1), 154.8 (1C, C3). HRMS (MALDI-TOF): *m*/*z* [M + H]^+^ calcd for C_33_H_46_N_3_O_2_: 516.3584; observed: 516.3587.

*N^3^-(1-adamantylmethyl)-N^1^-[2-(1-adamantyloxy)ethyl]isoquinoline-1,3-diamine* (**10b**). Obtained from chloroheterocycle **7** (71.5 mg), amine **4b** (132 mg) in the presence of Pd(dba)_2_ (5 mg), DavePhos (3.5 mg) and *t*BuONa (29 mg). Eluent CH_2_Cl_2_/MeOH 200:1. Yield 61 mg (62%), green viscous oil. ^1^H-NMR (CDCl_3_) δ 1.62–1.77 (m, 24H, Ad), 2.00 (s, 3H, Ad), 2.16 (s, 3H, Ad), 2.91 (s, 2H, CH_2_(NH)), 3.67–3.76 (m, 4H, CH_2_(NH), CH_2_(O)), 4.27 (br. s, 1H, NH), 5.82 (s, 1H, H4), 7.00–7.04 (m, 1H, H7), 7.32-7.37 (m, 2H, H5, H6), 7.51 (d, 1H, ^3^*J* = 8.4 Hz, H8), 1 NH proton was not unambiguously attributed. ^13^C-NMR(CDCl_3_) δ 28.4 (3C, Ad), 30.5 (3C, Ad), 33.9 (1C, C_quat_(Ad)), 36.4 (3C, Ad), 37.1 (3C, Ad), 40.7 (3C, Ad), 41.7 (4C, Ad, CH_2_(Ad)), 55.2 (1C, CH_2_(NH)), 58.9 (1C, CH_2_(O)), 72.4 (1C, C_quat_(O)), 84.9 (1C, C4), 113.0 (1C, C8a), 120.4 (1C, C5), 121.6 (1C, C8), 125.1 (1C, C7), 129.5 (1C, C6), 140.7 (1C, C4a), 154.7 (1C, C1), 155.1 (1C, C3). HRMS (MALDI-TOF): *m*/*z* [M + H]^+^ calcd for C_32_H_44_N_3_O: 486.3479; observed: 486.3506.

*N^3^-[2-(1-adamantyl)-1-methylethyl]-N^1^-[2-(1-adamantyloxy)ethyl]isoquinoline-1,3-diamine* (**10f**). Obtained from chloroheterocycle **7** (71.5 mg), amine **4f** (155 mg) in the presence of Pd(dba)_2_ (5 mg), DavePhos (3.5 mg) and *t*BuONa (29 mg). Eluent CH_2_Cl_2_/MeOH 200:1. Yield 69 mg (67%), green viscous oil. ^1^H-NMR (CDCl_3_) δ 1.23 (d, 3H, ^3^*J* = 6.2 Hz, CH_3_), 1.58–1.69 (m, 20H, Ad, CH_2_), 1.76–1.77 (m, 6H, Ad), 1.93 (s, 3H, Ad), 2.15 (s, 3H, Ad), 3.68–3.78 (m, 5H, CH_2_(NH), CH(NH), CH_2_(O)), 4.17 (br. s, 1H, NH), 5.81 (s, 1H, H4), 7.00–7.05 (m, 1H, H7), 7.33–7.38 (m, 2H, H5, H6), 7.52 (d, 1H, ^3^*J* = 8.3 Hz, H8), 1 NH proton was not unambiguously attributed. ^13^C-NMR(CDCl_3_) δ 23.4 (1C, CH_3_), 28.7 (3C, Ad), 30.5 (3C, Ad), 32.6 (1C, C_quat_(Ad)), 36.4 (3C, Ad), 37.0 (3C, Ad), 41.7 (4C, Ad, CH), 42.9 (3C, Ad), 43.3 (1C, CH_2_(NH)), 52.7 (1C, CH_2_(NH)), 58.7 (1C, CH_2_(O)), 72.4 (1C, C_quat_(O)), 85.1 (1C, C4), 112.9 (1C, C8a), 120.2 (1C, C5), 121.7 (1C, C8), 125.0 (1C, C7), 129.5 (1C, C6), 140.7 (1C, C4a), 153.2 (1C, C1), 154.4 (1C, C3). HRMS (MALDI-TOF): *m*/*z* [M + H]^+^ calcd for C_34_H_48_N_3_O: 514.3792; observed: 514.3806.

*N^3^-[2-(1-adamantyl)ethyl]-N^1^-[2-(1-adamantyloxy)ethyl]isoquinoline-1,3-diamine* (**10g**). Obtained from chloroheterocycle **7** (71.5 mg), amine **4g** (143 mg) in the presence of Pd(dba)_2_ (5 mg), DavePhos (3.5 mg) and *t*BuONa (29 mg). Eluent CH_2_Cl_2_/MeOH 150:1. Yield 43 mg (43%), green viscous oil. ^1^H-NMR (CDCl_3_) δ 1.57–1.77 (m, 26H, Ad, CH_2_), 1.98 (s, 3H, Ad), 2.15 (s, 3H, Ad), 3.17–3.21 (m, 2H, CH_2_(NH)), 3.65–3.71 (m, 4H, CH_2_(NH), CH_2_(O)), 5.69 (br. s, 1H, NH), 5.81 (s, 1H, H4), 7.04 (m, 1H, ^3^*J* = 8.1 Hz, H7), 7.34–7.40 (m, 2H, H5, H6), 7.51-7.54 (m, 1H, H8), 1 NH proton was not unambiguously attributed HRMS (MALDI-TOF): *m*/*z* [M + H]^+^ calcd for C_33_H_46_N_3_O: 500.3635; observed: 500.3653.

## 4. Conclusions

To sum up, we have investigated a synthetic approach to adamantane-containing 4,6-diaminopyrimidine, 2,6-diaminopyrazine, and 1,3-diaminoisoquinoline based on consequent substitution of chlorine atoms in the corresponding dichloroheterocycle by means of convenient nucleophilic substitution and Pd-catalyzed amination, respectively. Selective monoamination of 4,6-diaminopyrimidine proceeds under catalyst-free conditions with products yields from good to excellent. Synthesis of 4,6-diaminopyrimidine, 2,6-diaminopyrazine and 1,3-diaminoisoquinoline by Pd-catalyzed amination of aminochloroderivatives is complicated by *N,N*-diarylation, leading to formation of oligomers; therefore, to suppress the side products formation, 4-fold excess of amine is required. Adamantane-containing 4,6-dialkylaminopyrimidines and 1,3-dialkylaminoisoquinolines have been obtained in moderate to good yields (40–60%) using DavePhos as a ligand while the use of Ph-JosiPhos ligand is necessary for the synthesis of adamantylated 2,6-diaminopyrazines. It has been shown that adamantane-containing 6-chloro-4-aminopyrimidines exhibit prototropic tautomerism, which was observed and quantitatively characterized by NMR-spectroscopy. Tautomerism between amine and imine forms of the *N*-heteroarylated derivatives of adamantaneamines is held responsible to the formation of *N,N*-diarylated species.

## Figures and Tables

**Table 1:** Pd-catalyzed amination of 4-amino-6-chloropyrimidine **5a**.

Entry	Amine	Equiv. of Amine	Ligand	Pd(dba)_2_/L, mol%	Product, Yield, %
1	**4a**	1	BINAP	4/4.5	**8a**, oligomers ^1^
2	**4a**	2	Cy-JosiPhos	2/2.5	**8a**, oligomers ^1^
3	**4a**	2	DavePhos	4/4.5	**8a**, oligomers ^1^
4	**4a**	2	Ph-JosiPhos	4/4.5	**8a**, oligomers ^1^
5	**4a**	4	DavePhos	4/4.5	**8a**, 60
6	**4a**	4	BINAP	8/9	**8a**, 61
7	**4b**	4	DavePhos	4/4.5	**8b**, 40
8	**4f**	4	DavePhos	4/4.5	**8f**, 46

^1^ Side products were observed in ^1^H NMR spectra of the reaction mixtures and confirmed by MALDI-TOF mass spectroscopy.

**Table 2:** The activation energy characteristics of prototropic equlibrium of **5b,c,f,g**.

Entry	Compound	X	Reaction	K_273_	ΔH^≠^, kcal/mol	ΔS^≠^, cal/mol × K	ΔG^≠^_(273 K)_, kcal/mol
1	**5g**	1-CH_2_CH_2_	**A**→**B**	0.45	17.84	13.58	14.24
2	**5g**	1-CH_2_CH_2_	**B**→**A**		16.89	12.65	13.81
3	**5b**	1-CH_2_	**A**→**B**	0.495	19.30	17.51	14.67
4	**5b**	1-CH_2_	**B**→**A**		18.46	15.81	14.29
5	**5c**	2-CH_2_	**A**→**B**	0.54	16.31	7.38	14.48
6	**5c**	2-CH_2_	**B**→**A**		15.42	5.30	14.15
7	**5f**	1-CH_2_CH(CH_3_)	**A**→**B**	0.575	17.36	11.90	14.26
8	**5f**	1-CH_2_CH(CH_3_)	**B**→**A**		16.69	10.59	13.96

**Table 3:** Pd-catalyzed amination of 2-amino-6-chloropyrazine **6**.

Entry	Amine	Equiv. of Amine	Ligand	Product, Yield (%)
1	**4a**	1	BINAP	**9a**, oligomers ^a^
2	**4a**	1	DavePhos	**9a**, oligomers ^a^
3	**4a**	2	Cy-JosiPhos	**9a**, 30
4	**4a**	4	Ph-JosiPhos	**9a**, 90
5	**4b**	4	Ph-JosiPhos	**9b**, 42
6	**4f**	4	Ph-JosiPhos	**9f**, (47) ^b^
7	**4g**	4	Ph-JosiPhos	**9g**, 48
8	**4h**	4	Ph-JosiPhos	**9h**, 36

^a^ Side products were observed in ^1^H NMR spectra of the reaction mixtures and confirmed by MALDI-TOF mass spectroscopy (**(4a)_3_Het_2_**, [M + H]^+^ 738.48 and **(4a)_4_Het_3_**, [M + H]^+^ 1009.61). ^b^ Yield was determined by ^1^H NMR spectrum of the reaction mixture.

**Table 4:** Pd-catalyzed amination of 1-amino-3-chloroisoquinoline **7**.

Entry	Amine	Equiv. of Amine	Ligand	Pd(dba)_2_/L, mol%	Product, Yield
1	**4a**	2	Cy-JosiPhos	4/4.5	**10a**, **11**^a^
2	**4a**	2	DavePhos	4/4.5	**10a**, oligomers ^a^
3	**4a**	4	DavePhos	4/4.5	**10a**, 77%
4	**4a**	4	BINAP	8/9	**10a**, 76%
5	**4b**	4	DavePhos	4/4.5	**10b**, 62%
6	**4f**	4	DavePhos	4/4.5	**10f**, 67%
7	**4g**	4	DavePhos	4/4.5	**10g**, 43%

^a^ Side products were observed in NMR spectra of the reaction mixtures and confirmed by MALDI-TOF mass spectroscopy (e.g., **(4a)_3_Het_2_**, [M + H]^+^ 836.52).

## Data Availability

The data presented in this study are available in Supplementary Materials or on request from the corresponding author.

## Supplementary Materials

The following are available online. Experimental details of studies of prototropic equilibria.

## Sample Availability

Samples of the compounds are not available from the authors.
