# Supplementary material for: Mono- and Diamination of 4,6-Dichloropyrimidine, 2,6-Dichloropyrazine and 1,3-Dichloroisoquinoline with Adamantane-Containing Amines
Source: Molecules. 2021 Mar 29;26(7):1910. doi: 10.3390/molecules26071910 (PMC8037717; doi:10.3390/molecules26071910)
Supplement: Supplementary file 1 [file molecules-26-01910-s001.pdf]

Supporting information

for the article

Mono- and diamination of 4,6-dichloropyrimidine, 2,6-dichloropyrazine and 1,3-dichloroisoquinoline with adamantane-containing amines

by
Alisa D. Kharlamova, Anton S. Abel, Alexei D. Averin, Olga A. Maloshitskaya,
Vitaly A. Rozniatovskiy, Evgenii N. Savelyev, Boris S. Orlinson, Ivan A. Novakov
and Irina P. Beletskaya

Study of prototropic tautomerism of adamantine-containing 4-amino-6-chloropyrimidines

General information.
^{1}H NMR spectra were registered at Agilent 400-MR spectrometer (400 MHz) in $CDCl_3$. The spectra were fitted using WinDNMR software. Kinetic parameters were calculated from the dependence of the chemical shift of the proton in position 2 of the pyrimidine core on the temperature. As a result rate constant ($k = k_a + k_b$) and equilibrium constant ($K = k_a / k_b$) were calculated from the tautomer ratio for each temperature. $\Delta H^{\neq}$ and $\Delta S^{\neq}$ were calculated using Eyring equation (1) .

$$\ln \frac{k}{T} = \frac{-\Delta H^{\dagger}}{R} \frac{1}{T} + \ln \frac{k_B}{h} + \frac{\Delta S^{\ddagger}}{R} \quad (1)$$

The standard deviation of the coefficients determined is ±2.5%

Compound 5g

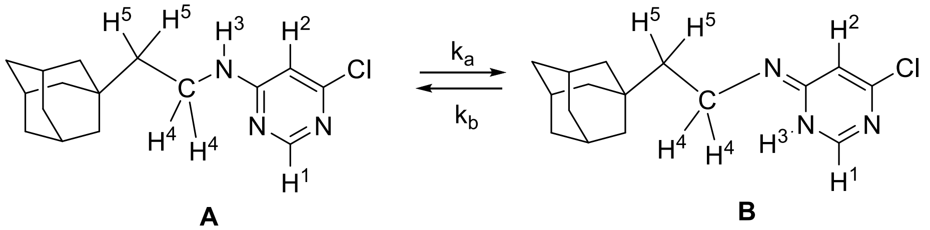

The ^{1}H NMR spectra were registered at temperatures in the range 243-308 K. The chemical shifts of proton signals at 243 K and 308 K are given in the table S1.

**Table S1.** Selected chemical shifts of proton signals at 243 K and 308 K for **5g**			
Proton	T = 243 K		T = 308 K
	**A** (major)	**B** (minor)	Average
H_1	8.21	8.33	8.28
H_2	6.27	6.33	6.27
H_3	6.91	5.56	5.18
H_4	3.08	3.35	3.25
H_5	1.36	1.35	1.36

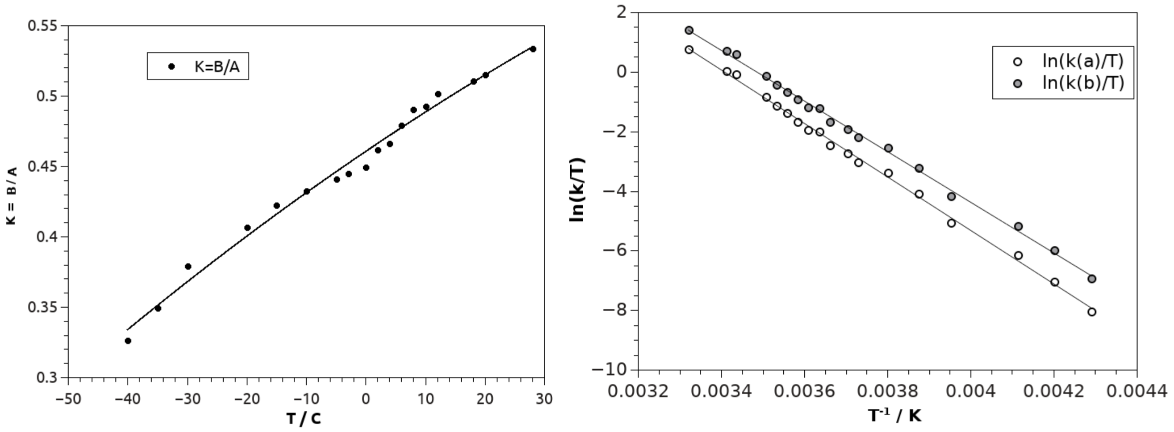

(a) **(b)**

Figure S1. (a) Dependence B/A ratio on the temperature for **5g**. **(b)** Dependence of ln (k/T) on 1/T for **5g**.

**Table S2.** The activation energy characteristics of prototropic transformation of **5g**		
	k_a (A → B)	k_b (B → A)
$\Delta H^{\neq}$ (kcal*mol^{-1})	17.84	16.89
$\Delta S^{\neq}$ (cal*mol^{-1}*T^{-1})	13.58	11.65
$\Delta G^{\neq}_{(273\ K)}$ (kcal*mol^{-1})	14.24	13.81

Compound 5b

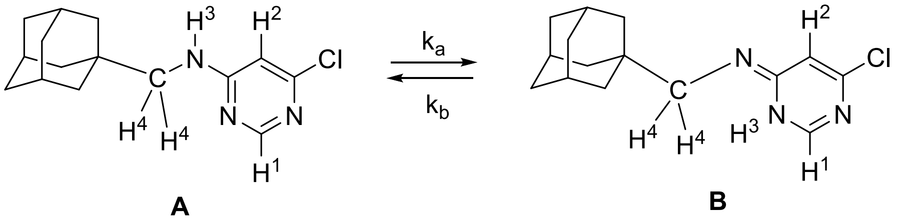

The ^{1}H NMR spectra were registered at temperatures in the range 233-303 K. The chemical shifts of proton signals at 233 K and 303 K are given in the table S3.

**Table S3.** Selected chemical shifts of proton signals at 233 K and 303 K for **5b**			
Proton	T = 233 K		T = 303 K
	**A** (major)	**B** (minor)	Average
H_1	8.19	8.28	8.25
H_2	6.35	6.48	6.33
H_3	7.09	6.27	5.45
H_4	2.80	3.13	2.95

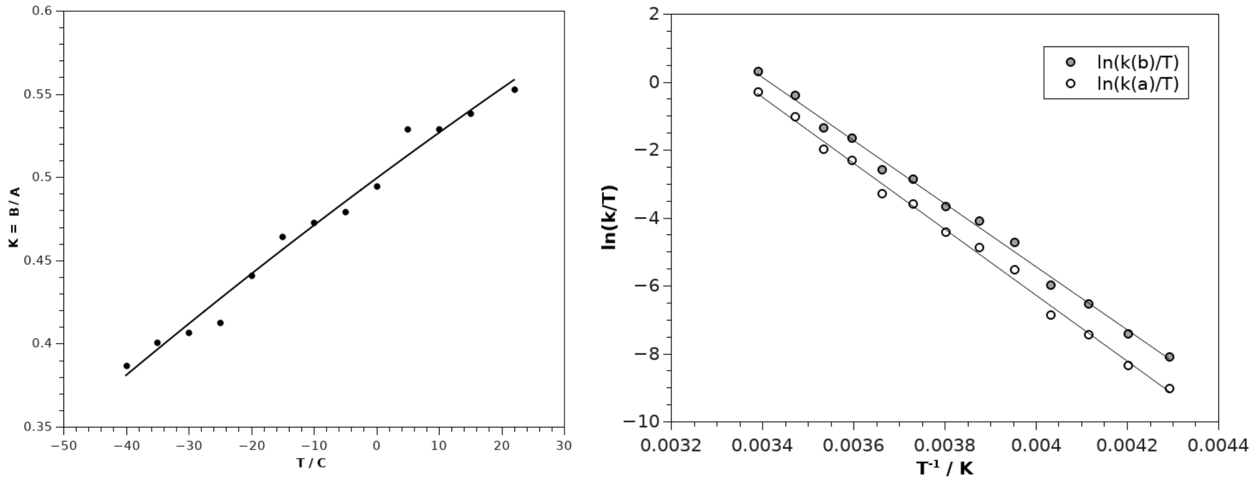

(a) **(b)**

Figure S2. (a) Dependence B/A ratio on the temperature for **5b**. **(b)** Dependence of ln (k/T) on 1/T for **5b**.

**Table S4.** The activation energy characteristics of prototropic transformation of **5b**		
	k_a (A → B)	k_b (B → A)
$\Delta H^{\neq}$ (kcal*mol^{-1})	19.30	18.46
$\Delta S^{\neq}$ (cal*mol^{-1}*T^{-1})	17.51	15.81
$\Delta G^{\neq}_{(273\ K)}$ (kcal*mol^{-1})	14.67	14.29

Compound 5c

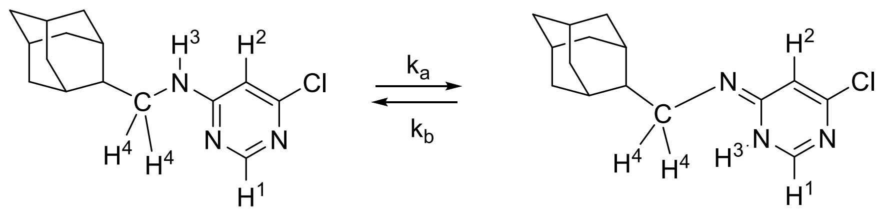

The ^{1}H NMR spectra were registered at temperatures in the range 233-313 K. The chemical shifts of proton signals at 233 K and 313 K are given in the table S5.

Table S5. Selected chemical shifts of proton signals at 233 K and 313 K for **5c**

Proton	T = 233 K		T = 313 K
	**A** (major)	**B** (minor)	Average
H_1	8.16	8.27	8.25
H_2	6.28	6.35	6.29
H_3	7.45	6.35	5.53
H_4	3.54	4.26	3.97

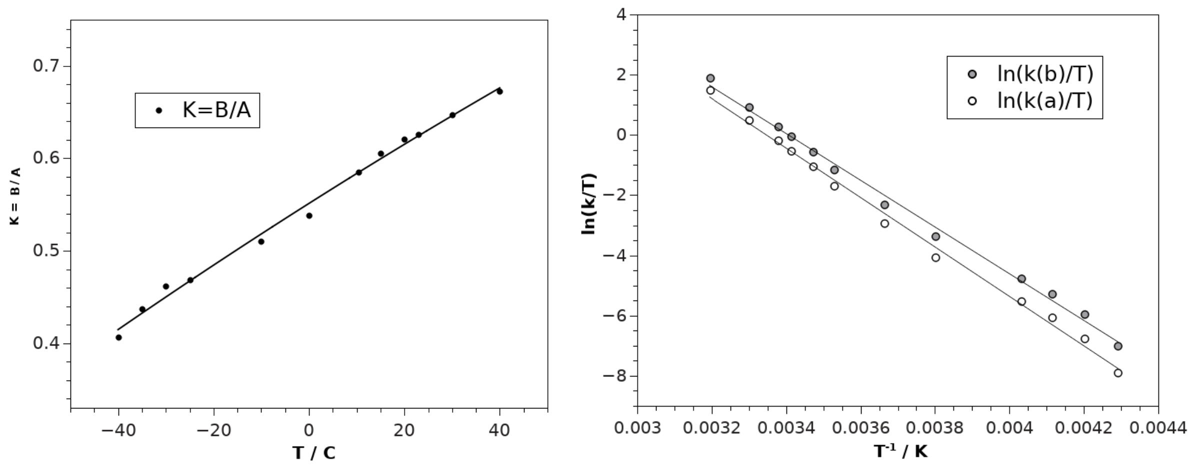

(a) **(b)**

Figure S3. (a) Dependence B/A ratio on the temperature for **5c**. **(b)** Dependence of ln (k/T) on 1/T for **5c**.

Table S6. The activation energy characteristics of prototropic transformation of **5c**

	k_a (A → B)	k_b (B → A)
$\Delta H^{\neq}$ (kcal*mol^{-1})	16.31	15.42
$\Delta S^{\neq}$ (cal*mol^{-1}*T^{-1})	7.38	5.30
$\Delta G^{\neq}_{(273\ K)}$ (kcal*mol^{-1})	14.48	14.15

Compound 5f

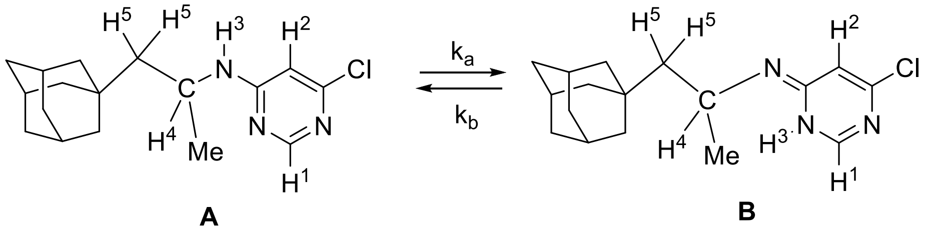

The ^{1}H NMR spectra were registered at temperatures in the range 223-323 K. The chemical shifts of proton signals at 223 K and 323 K are given in the table S7.

**Table S7.** Selected chemical shifts of proton signals at 223 K and 323 K for **5f**			
Proton	T = 223 K		T = 323 K
	**A** (major)	**B** (minor)	Average
H_1	8.24	8.33	8.29
H_2	6.32	6.31	6.26
H_3	6.84	5.65	4.96
H_4	3.17	3.47	3.36
H_5	1.25	1.25	1.28

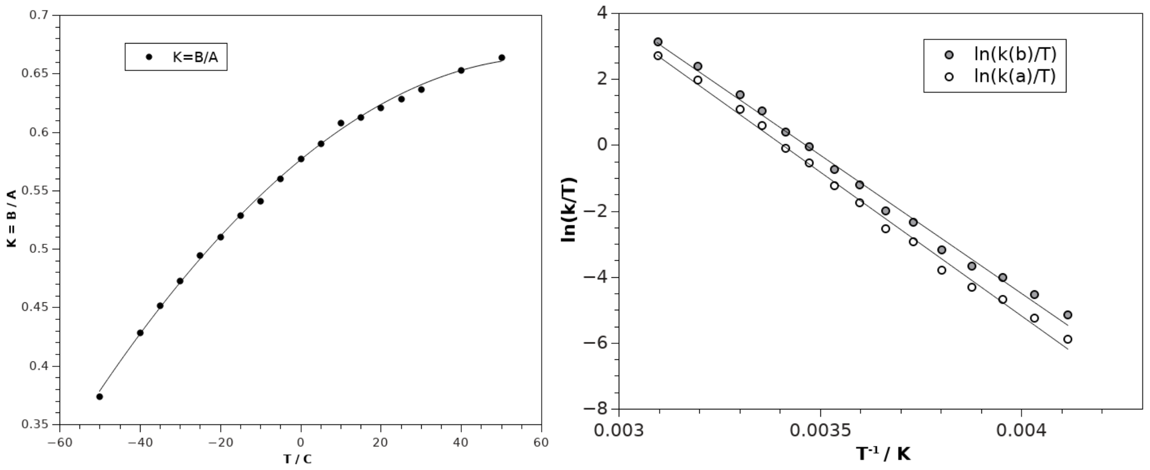

Figure S4. (a) Dependence B/A ratio on the temperature for **5f**. **(b)** Dependence of ln (k/T) on 1/T for **5f**.

**Table S8.** The activation energy characteristics of prototropic transformation of **5f**		
	k_a (A → B)	k_b (B → A)
$\Delta H^{\neq}$ (kcal*mol^{-1})	17.36	16.69
$\Delta S^{\neq}$ (cal*mol^{-1}*T^{-1})	11.90	10.59
$\Delta G^{\neq}_{(273\ K)}$ (kcal*mol^{-1})	14.26	13.96

NMR spectra

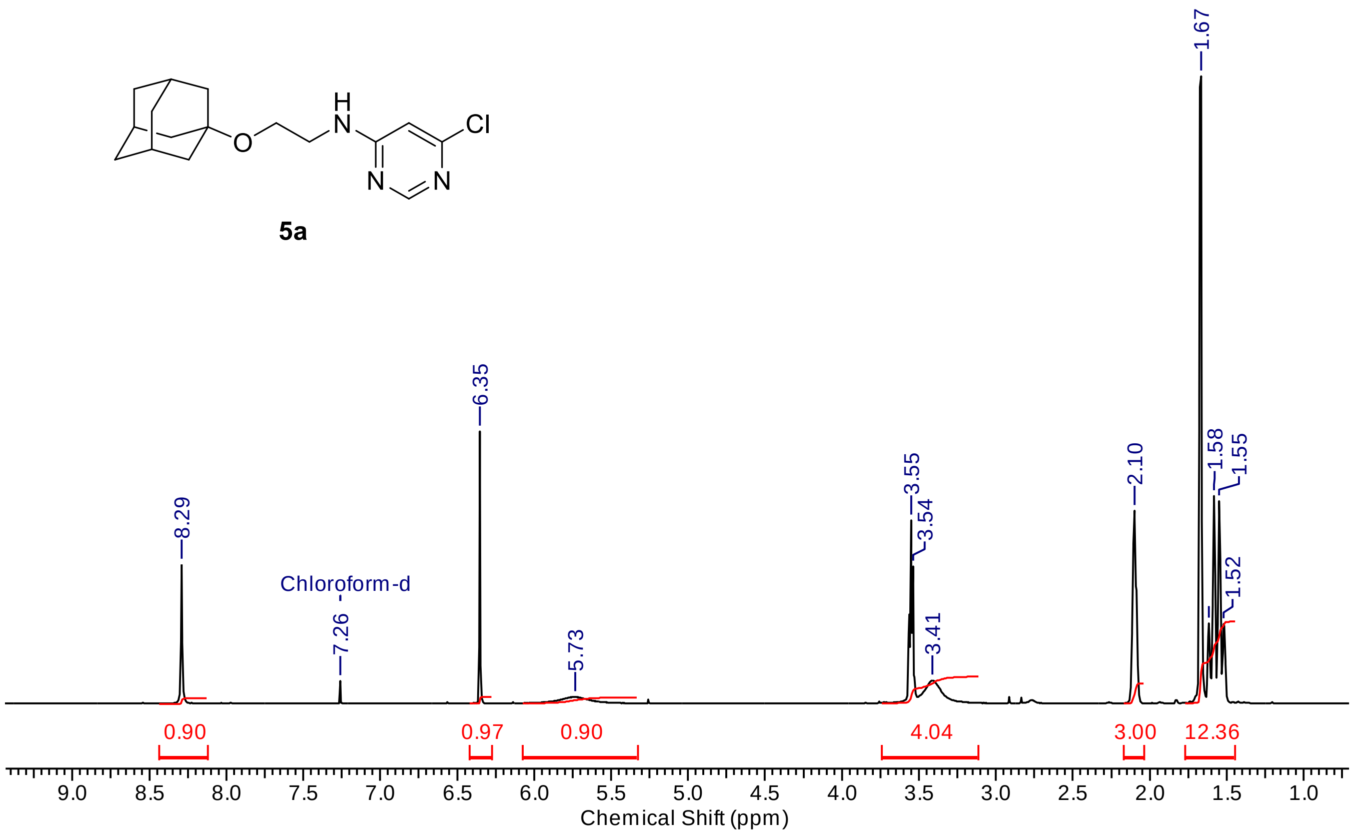

Figure S5. ^{1}H NMR spectrum of **5a** ($CDCl_3$, 400MHz, 300K).

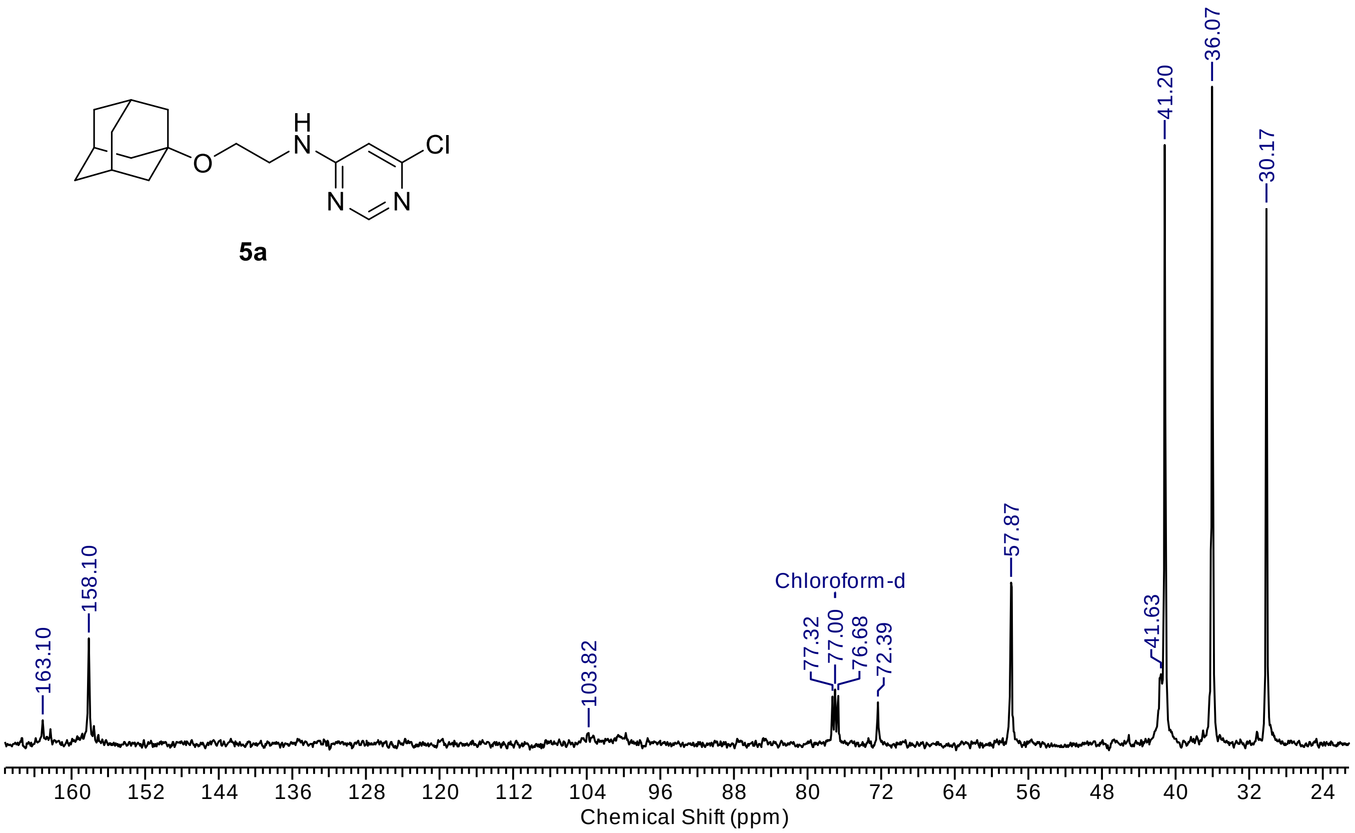

Figure S6. ^{13}C NMR spectrum of **5a** ($CDCl_3$, 100.6 MHz, 300K).

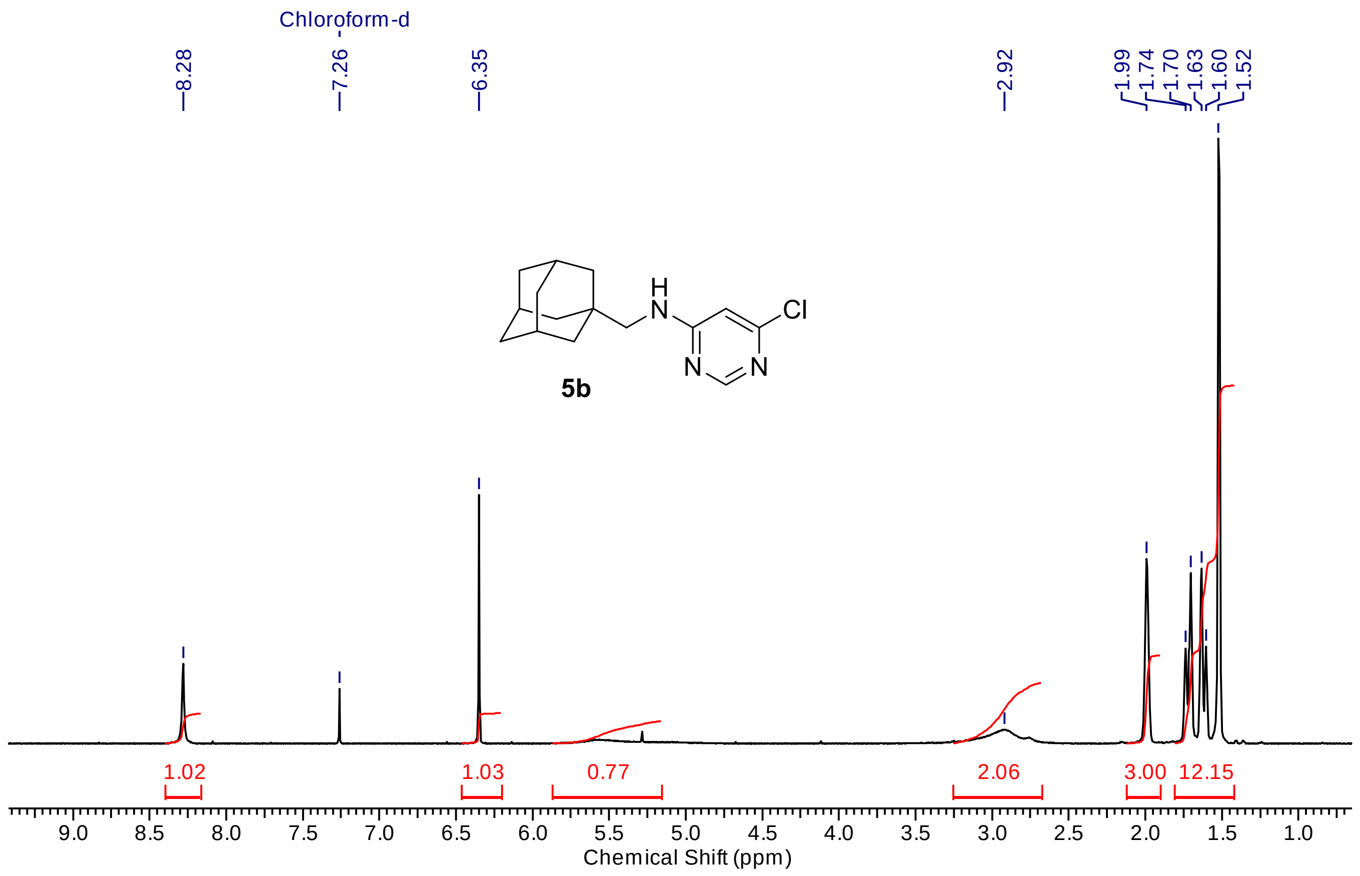

Figure S7. ^{1}H NMR spectrum of **5b** ($CDCl_3$, 400MHz, 300K).

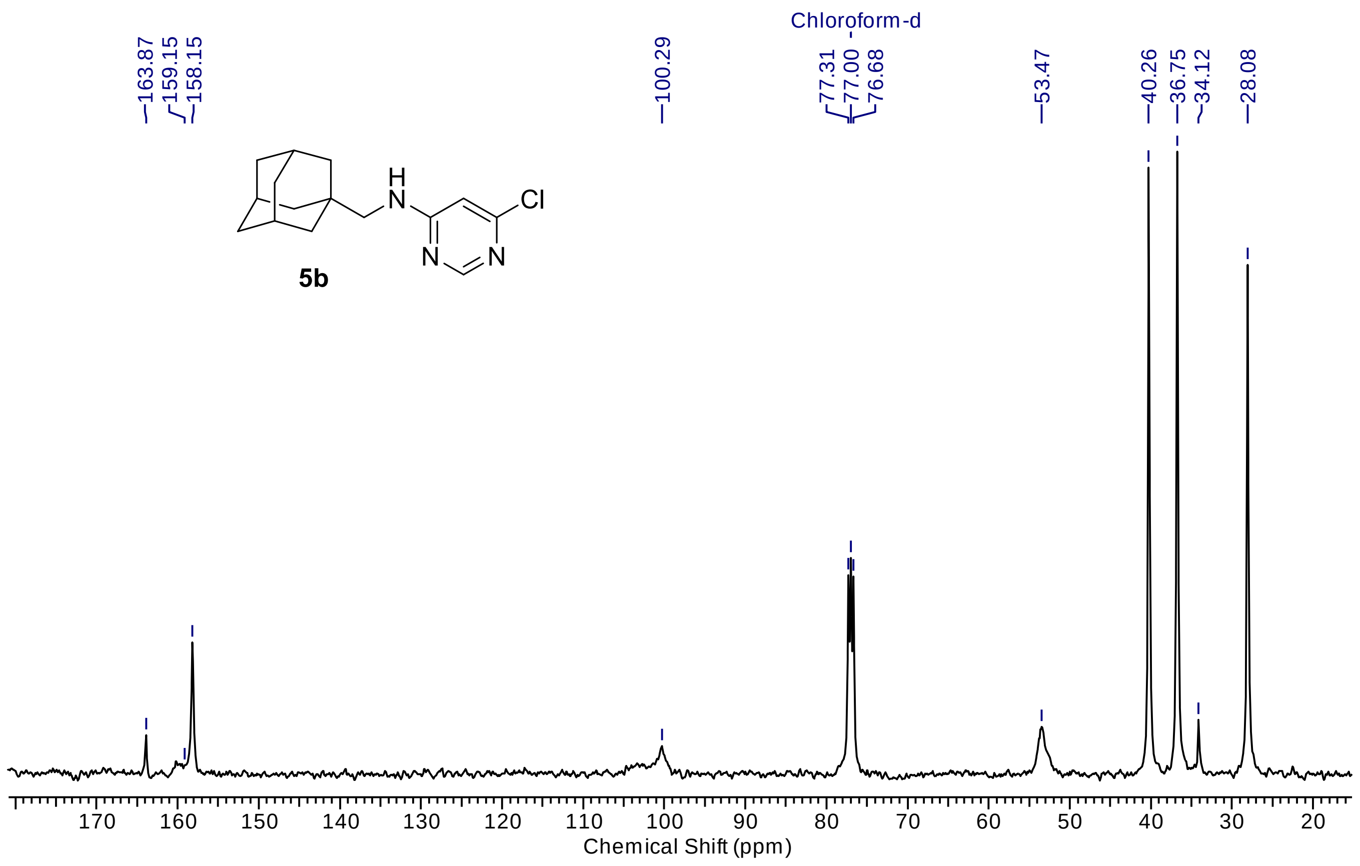

Figure S8. ^{13}C NMR spectrum of **5b** ($CDCl_3$, 100.6 MHz, 300K).

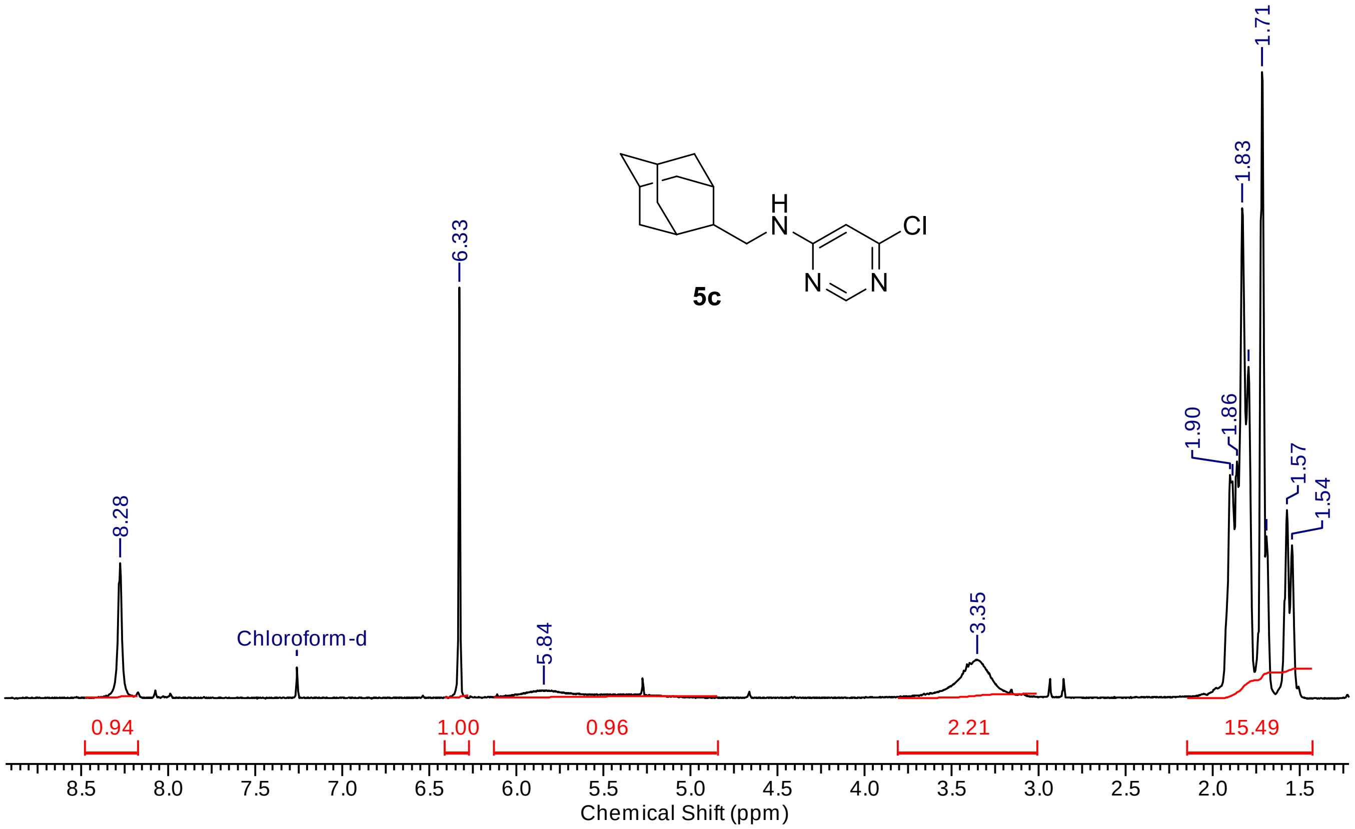

Figure S9. ^{1}H NMR spectrum of **5c** ($CDCl_3$, 400MHz, 300K).

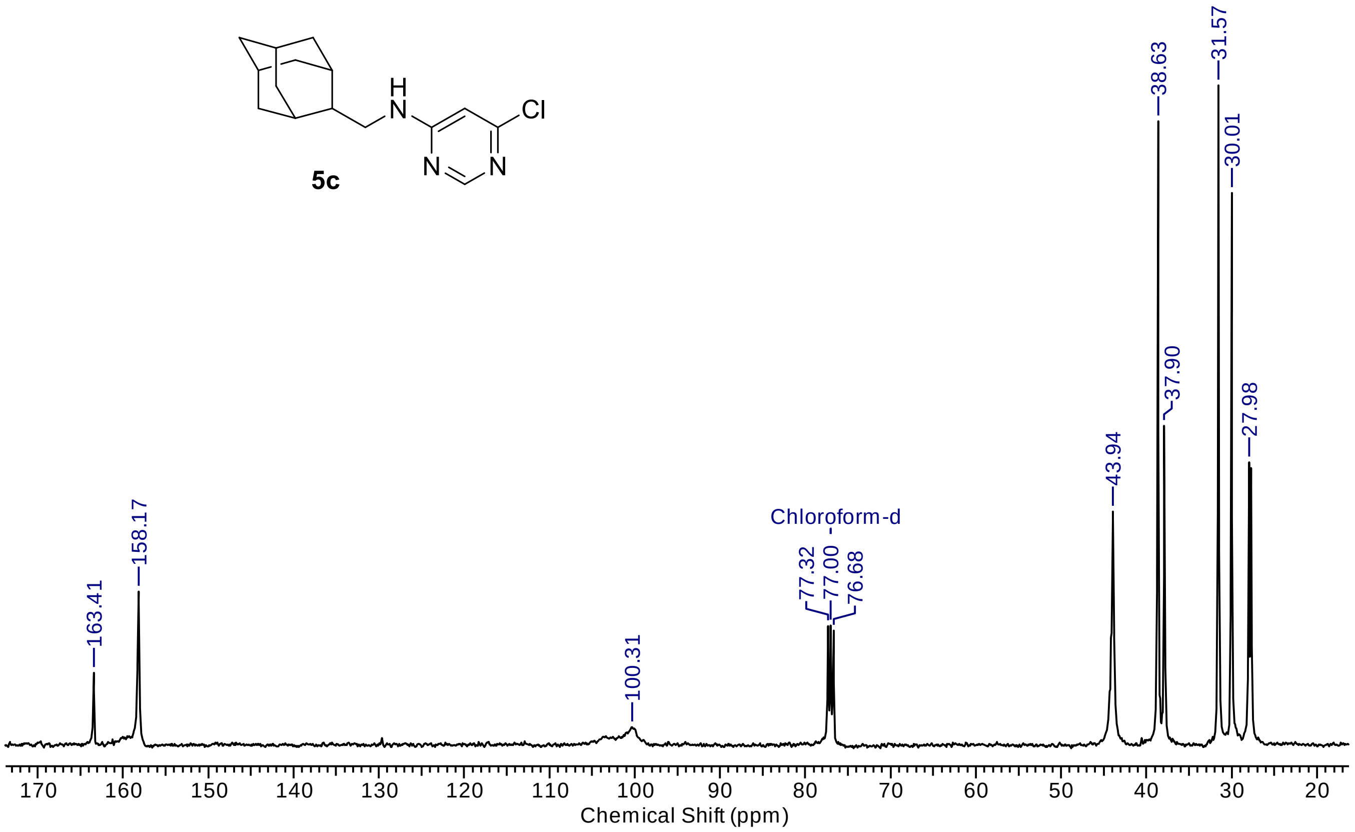

Figure S10. ^{13}C NMR spectrum of **5c** ($CDCl_3$, 100.6 MHz, 300K).

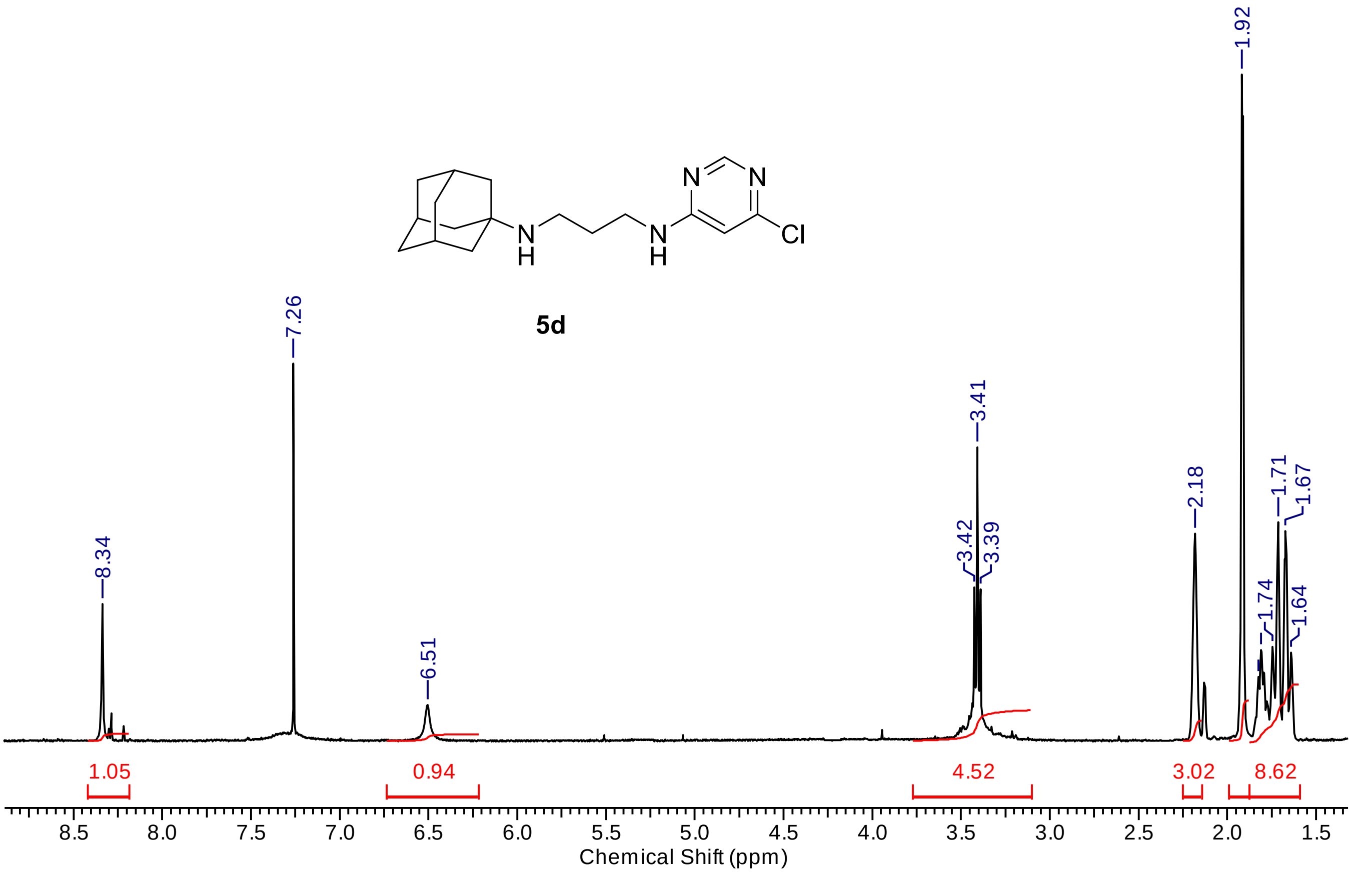

Figure S11. ^{1}H NMR spectrum of **5d** ($CDCl_3$, 400MHz, 300K).

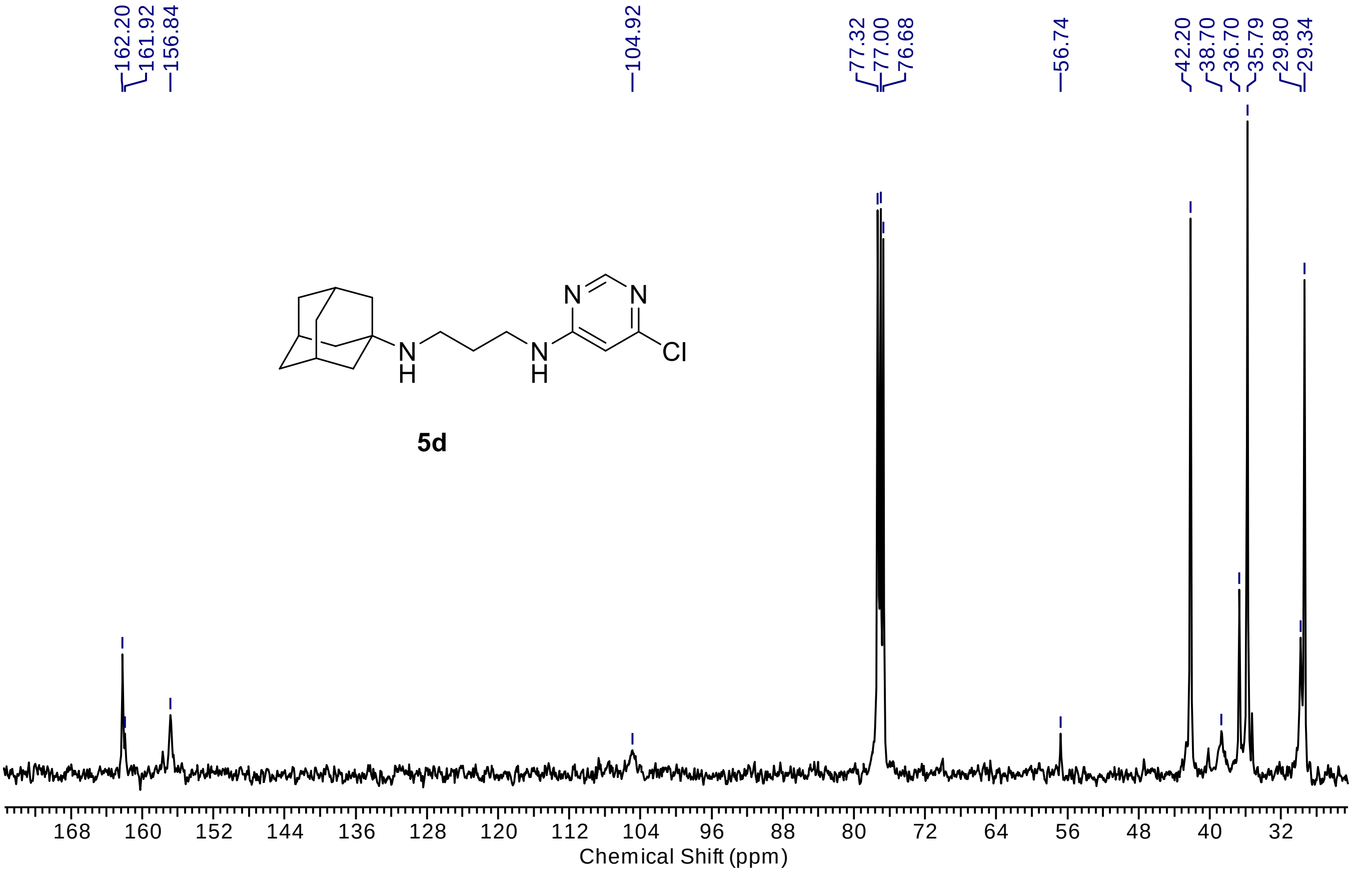

Figure S12. ^{13}C NMR spectrum of **5d** ($CDCl_3$, 100.6 MHz, 300K).

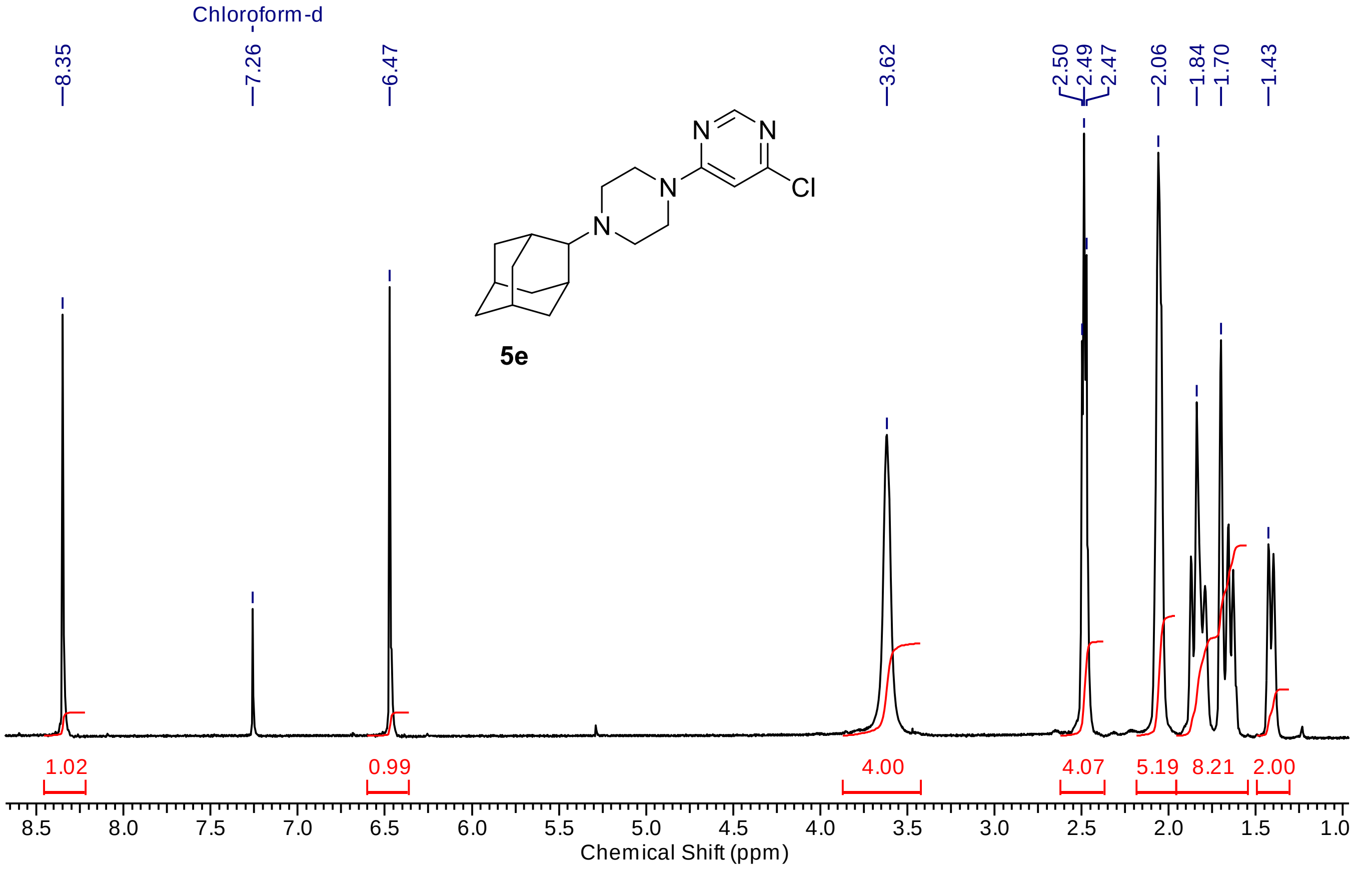

Figure S13. ^{1}H NMR spectrum of **5e** ($CDCl_3$, 400MHz, 300K).

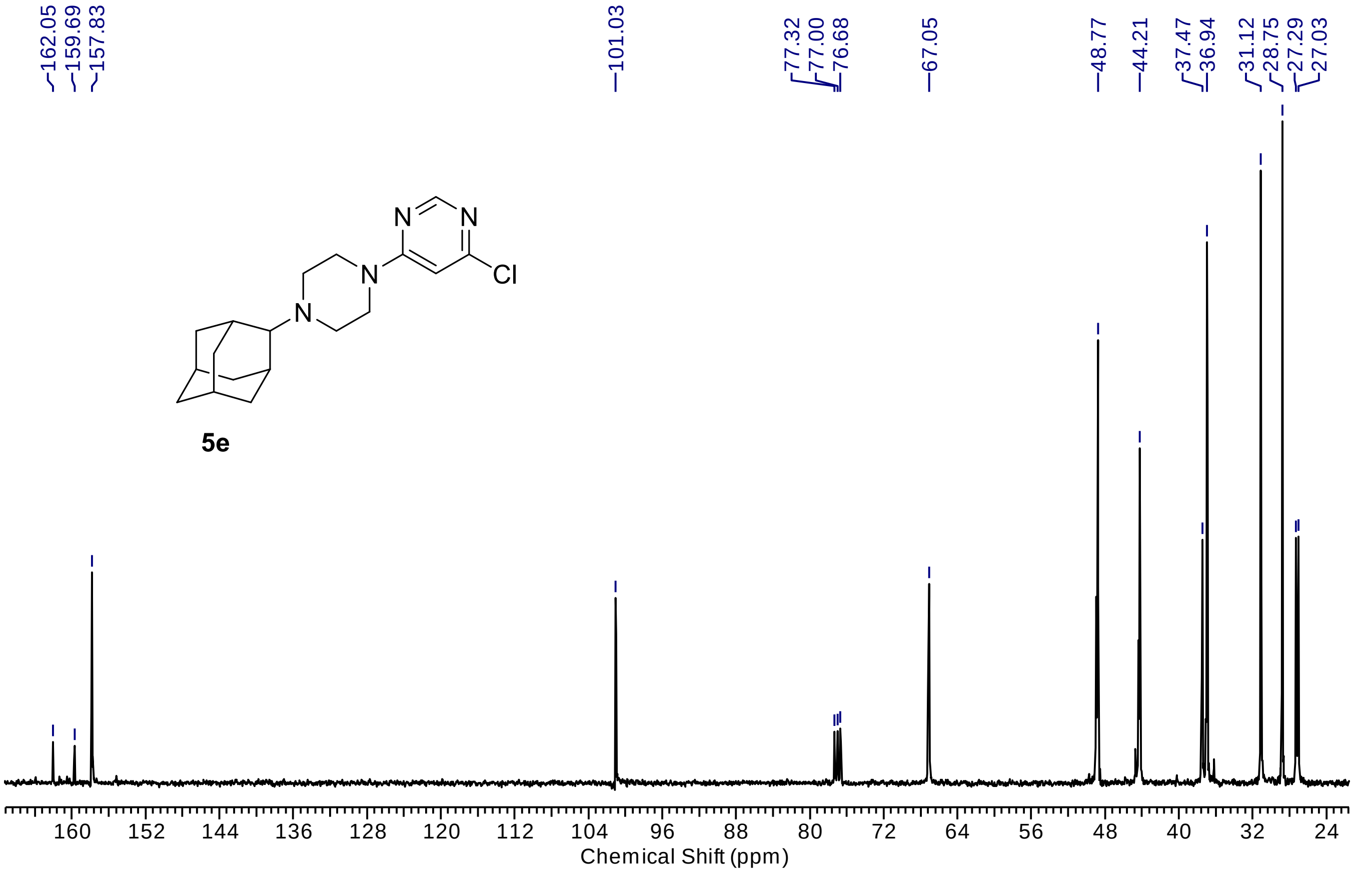

Figure S14. ^{13}C NMR spectrum of **5e** ($CDCl_3$, 100.6 MHz, 300K).

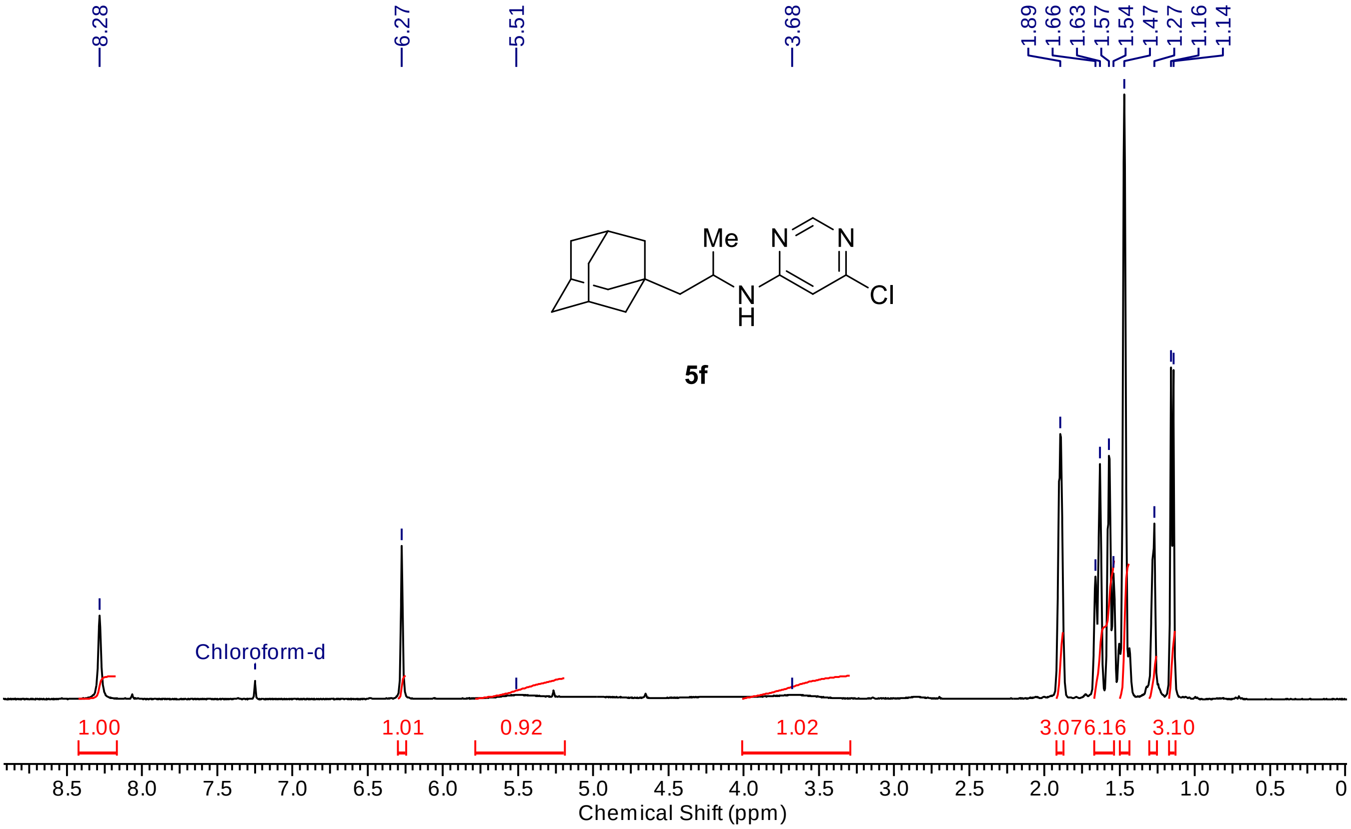

Figure S15. ^{1}H NMR spectrum of **5f** ($CDCl_3$, 400MHz, 300K).

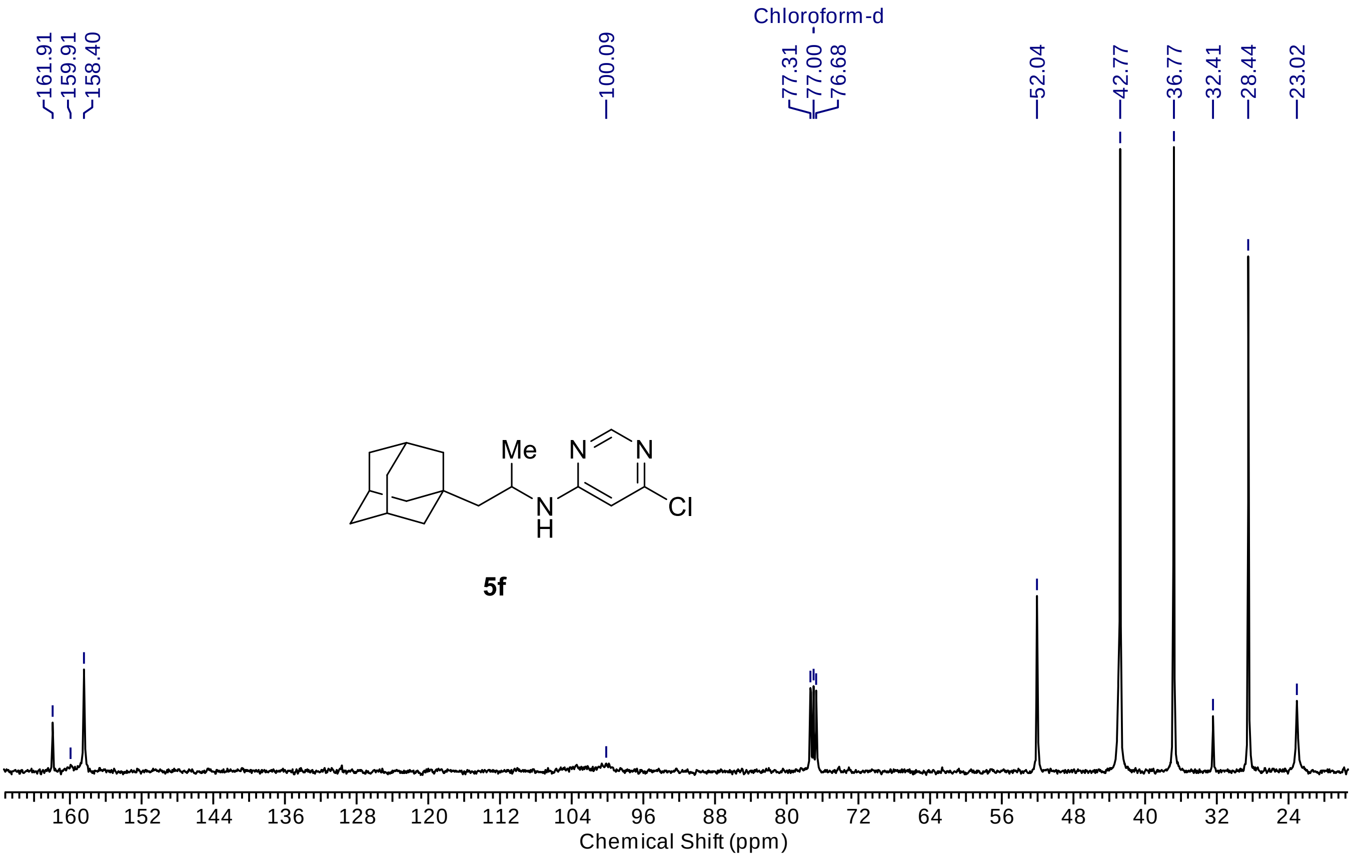

Figure S16. ^{13}C NMR spectrum of **5f** ($CDCl_3$, 100.6 MHz, 300K).

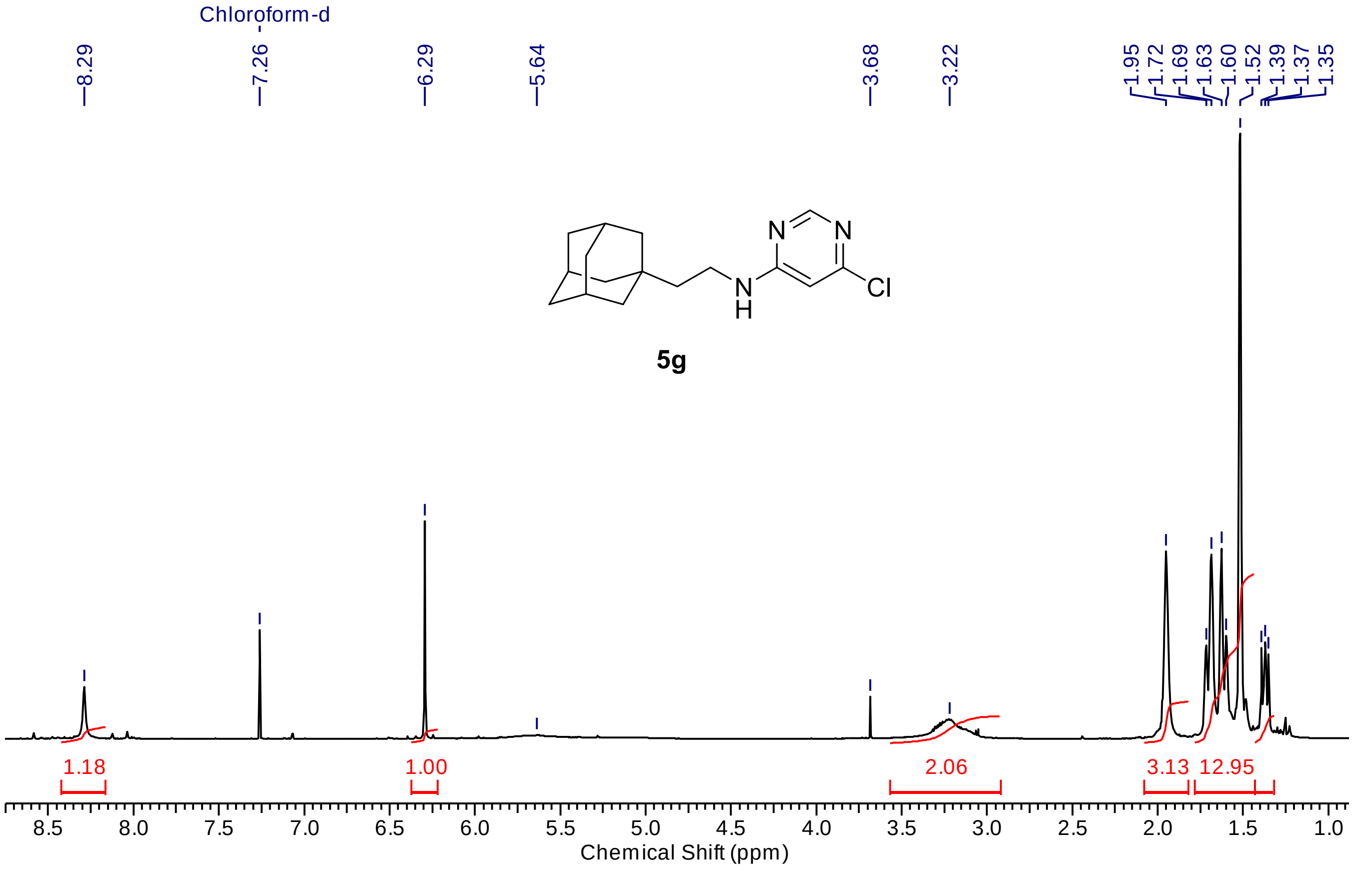

Figure S17. ^{1}H NMR spectrum of **5g** ($CDCl_3$, 400MHz, 300K).

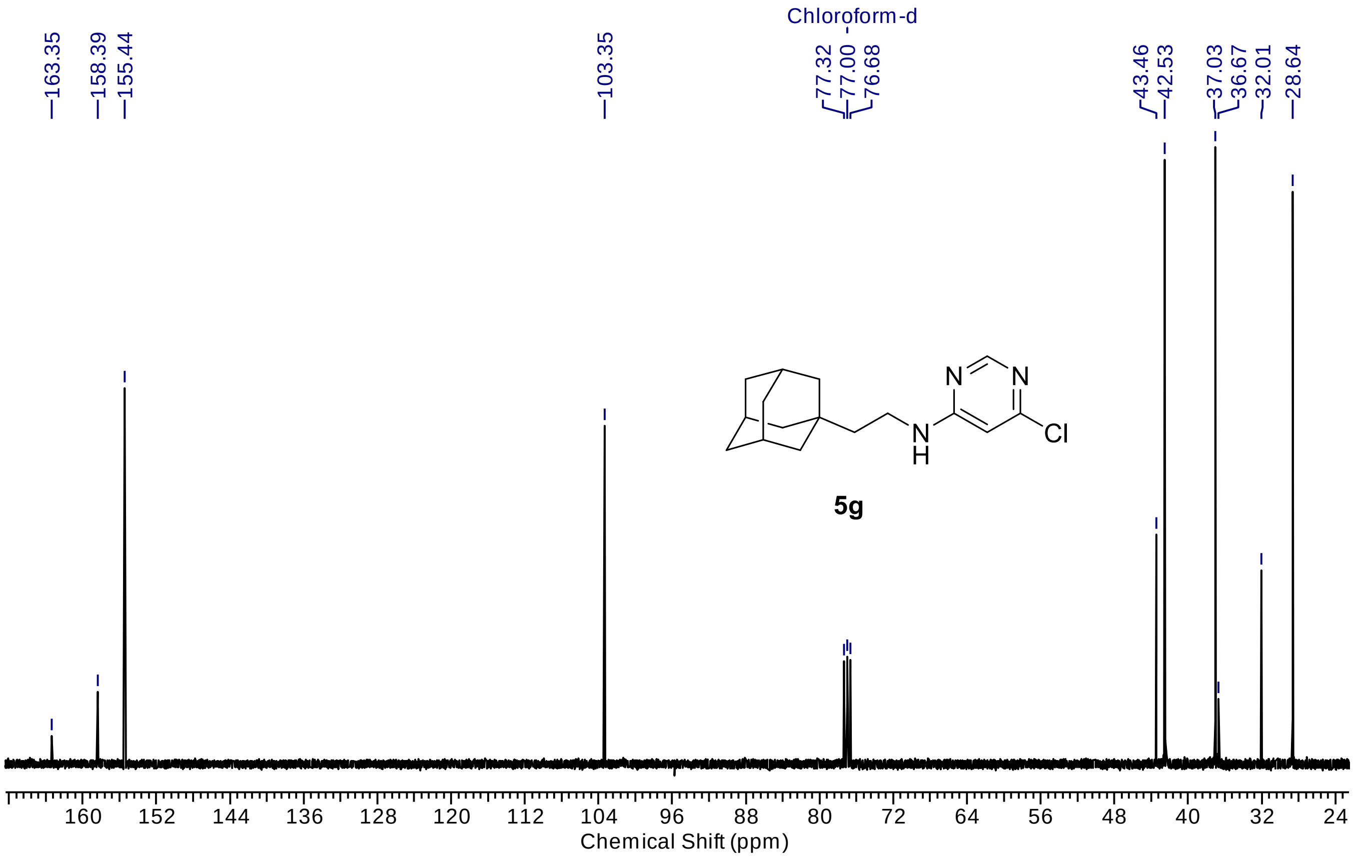

Figure S18. ^{13}C NMR spectrum of **5g** ($CDCl_3$, 100.6 MHz, 300K).

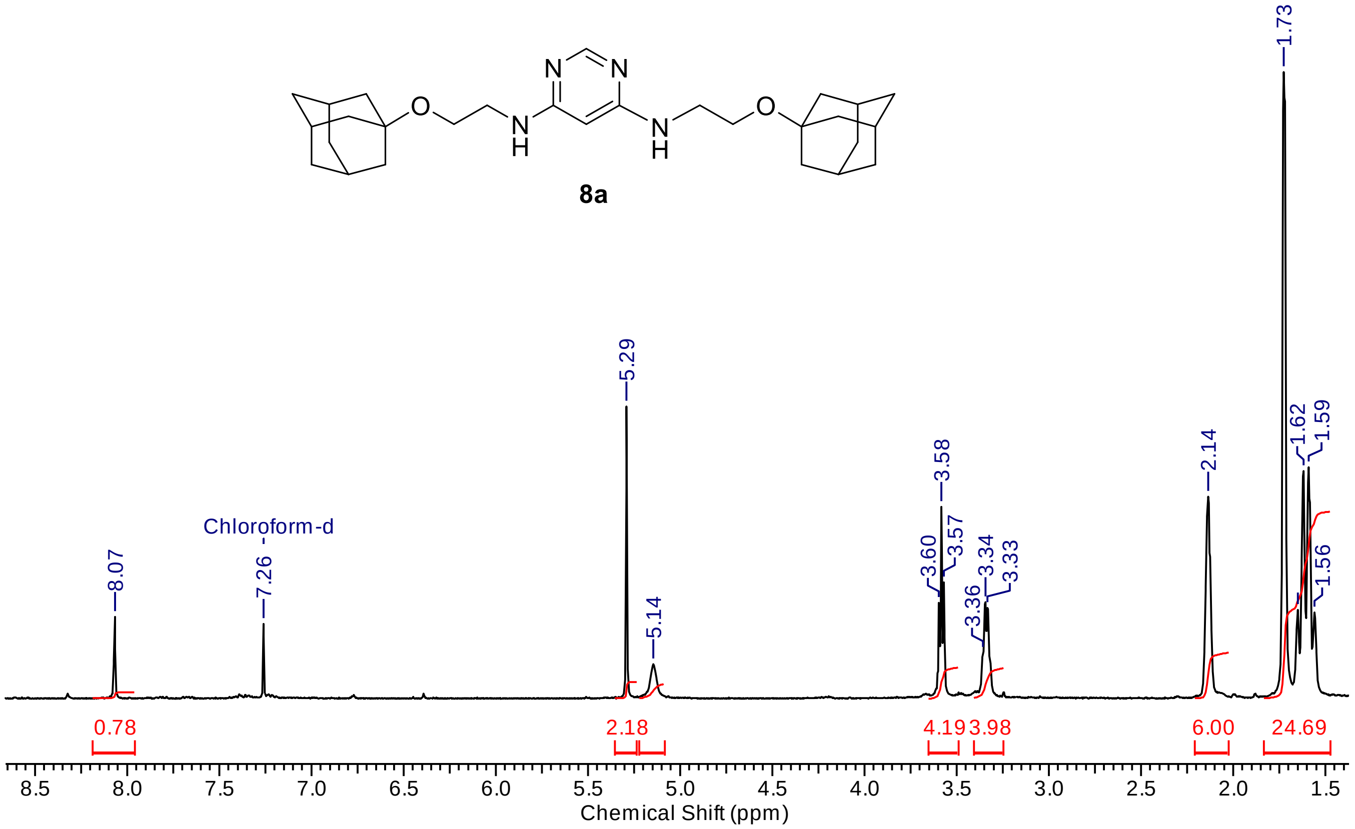

Figure S19. ^{1}H NMR spectrum of **8a** ($CDCl_3$, 400MHz, 300K).

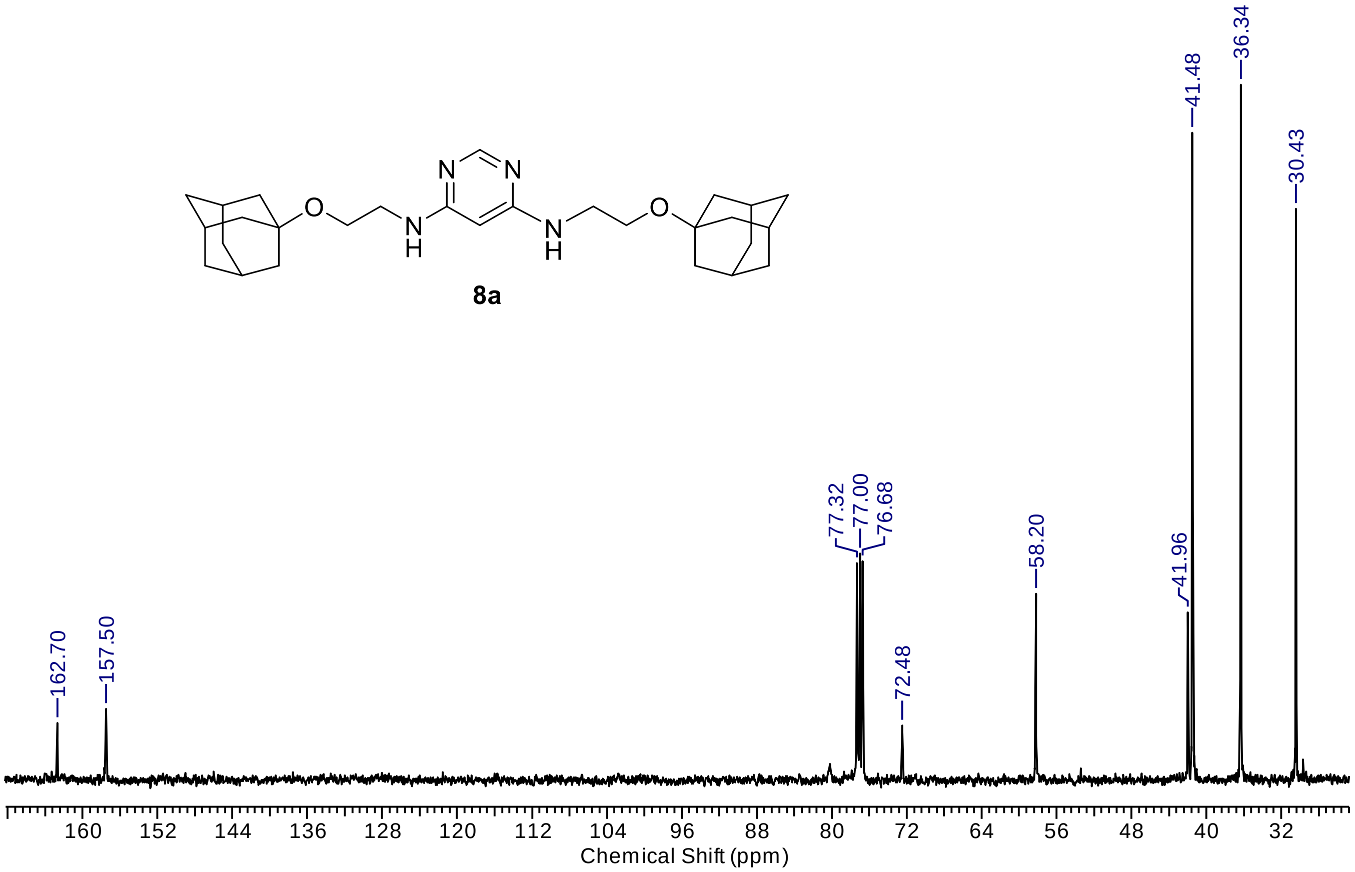

Figure S20. ^{13}C NMR spectrum of **8a** ($CDCl_3$, 100.6 MHz, 300K).

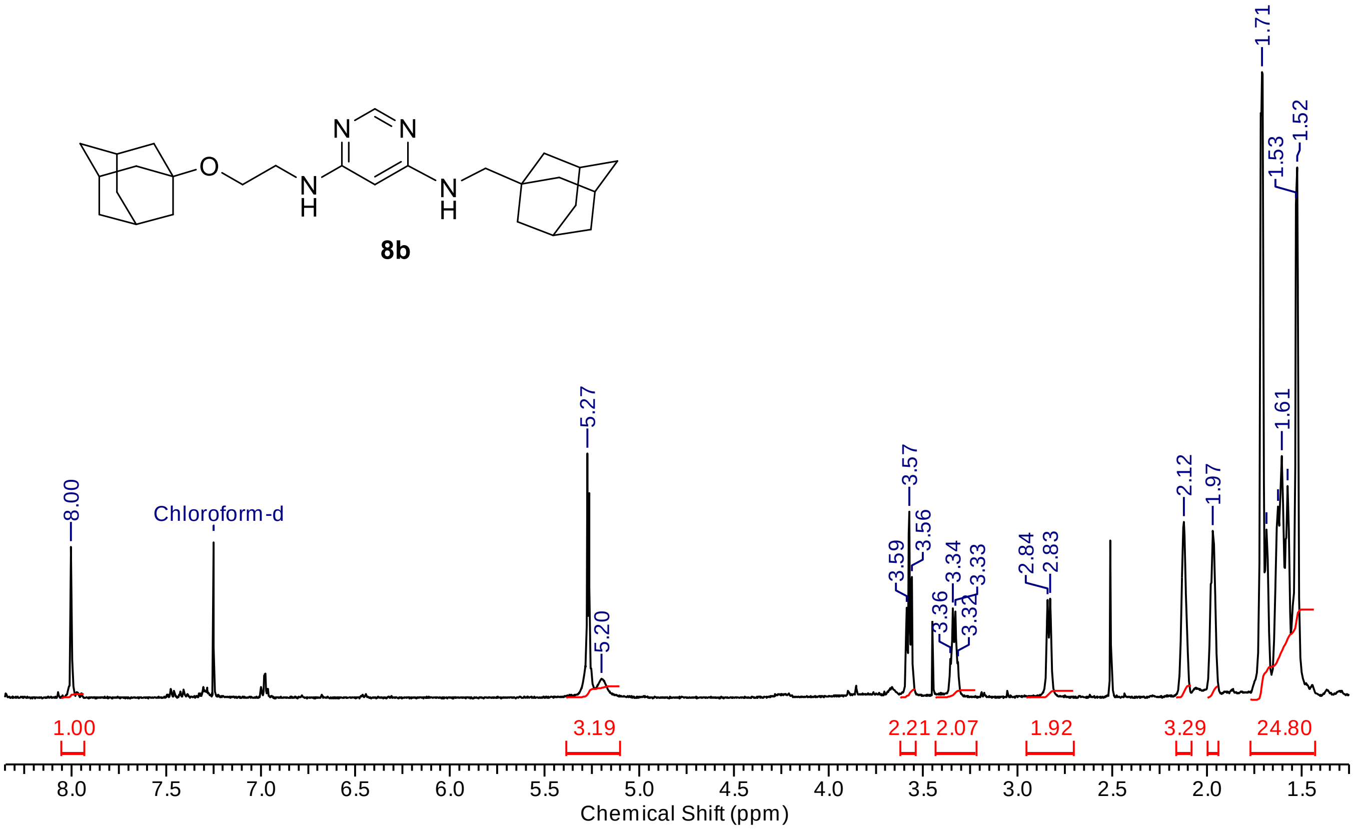

Figure S21. ^{1}H NMR spectrum of **8b** ($CDCl_3$, 400MHz, 300K).

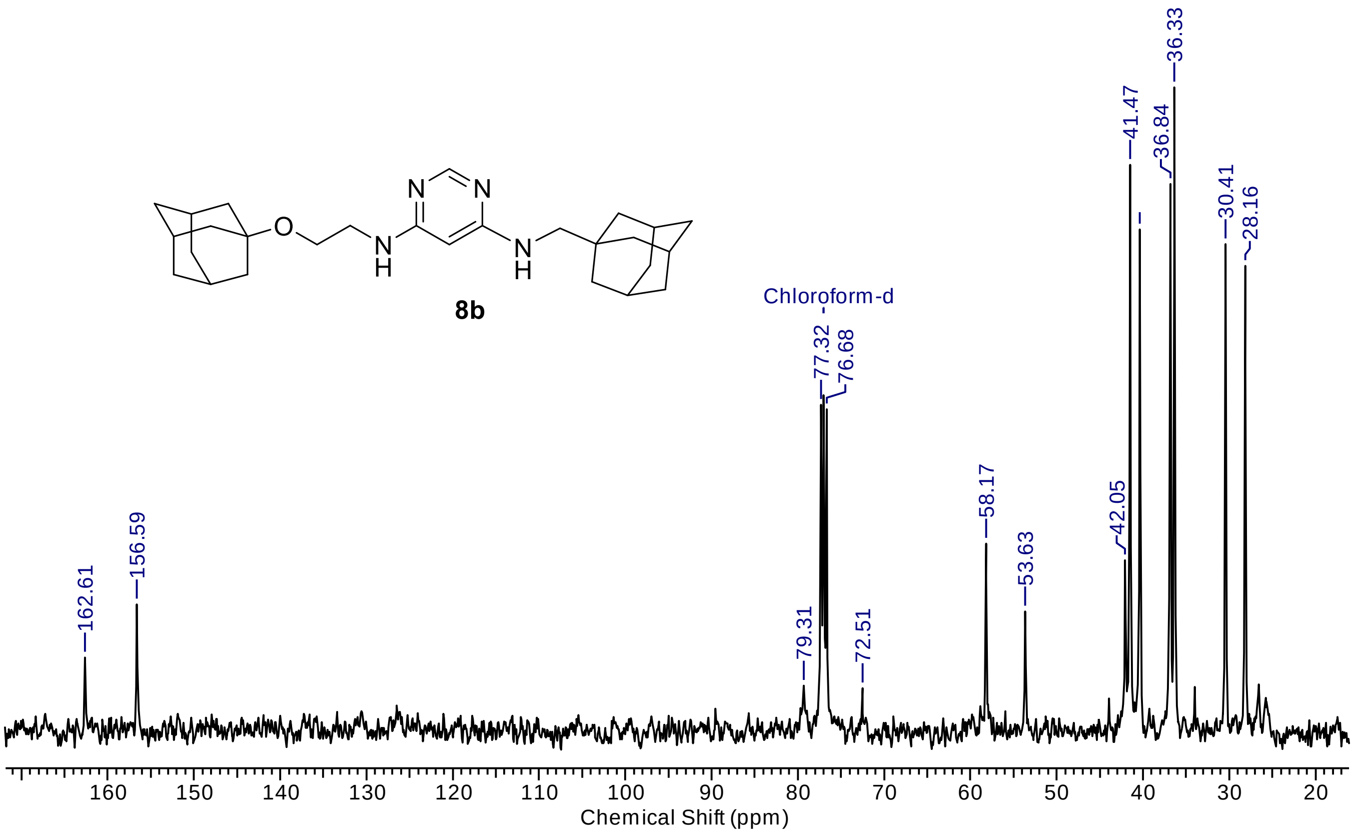

Figure S22. ^{13}C NMR spectrum of **8b** ($CDCl_3$, 100.6 MHz, 300K).

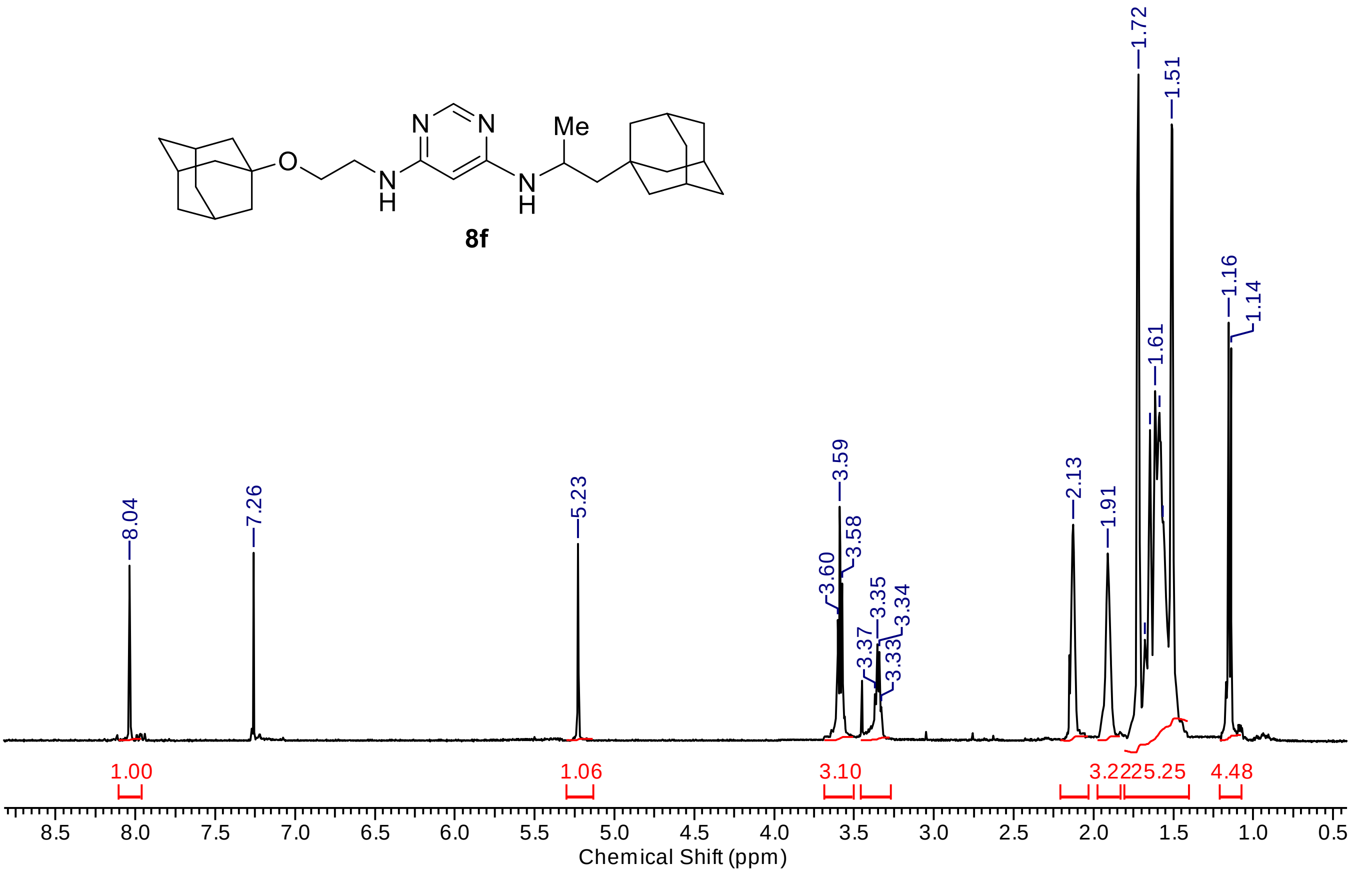

Figure S23. ^{1}H NMR spectrum of **8f** ($CDCl_3$, 400MHz, 300K).

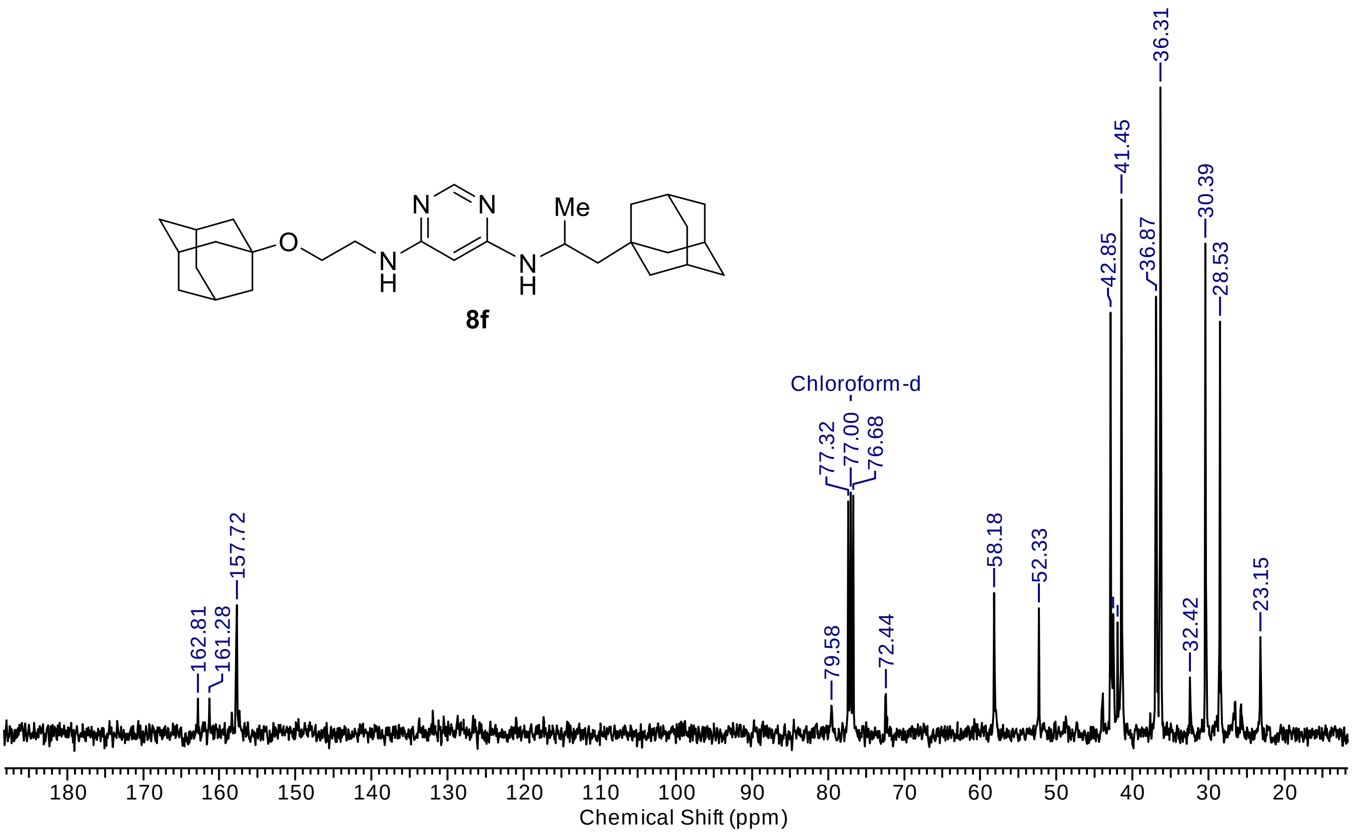

Figure S24. ^{13}C NMR spectrum of **8f** ($CDCl_3$, 100.6 MHz, 300K).

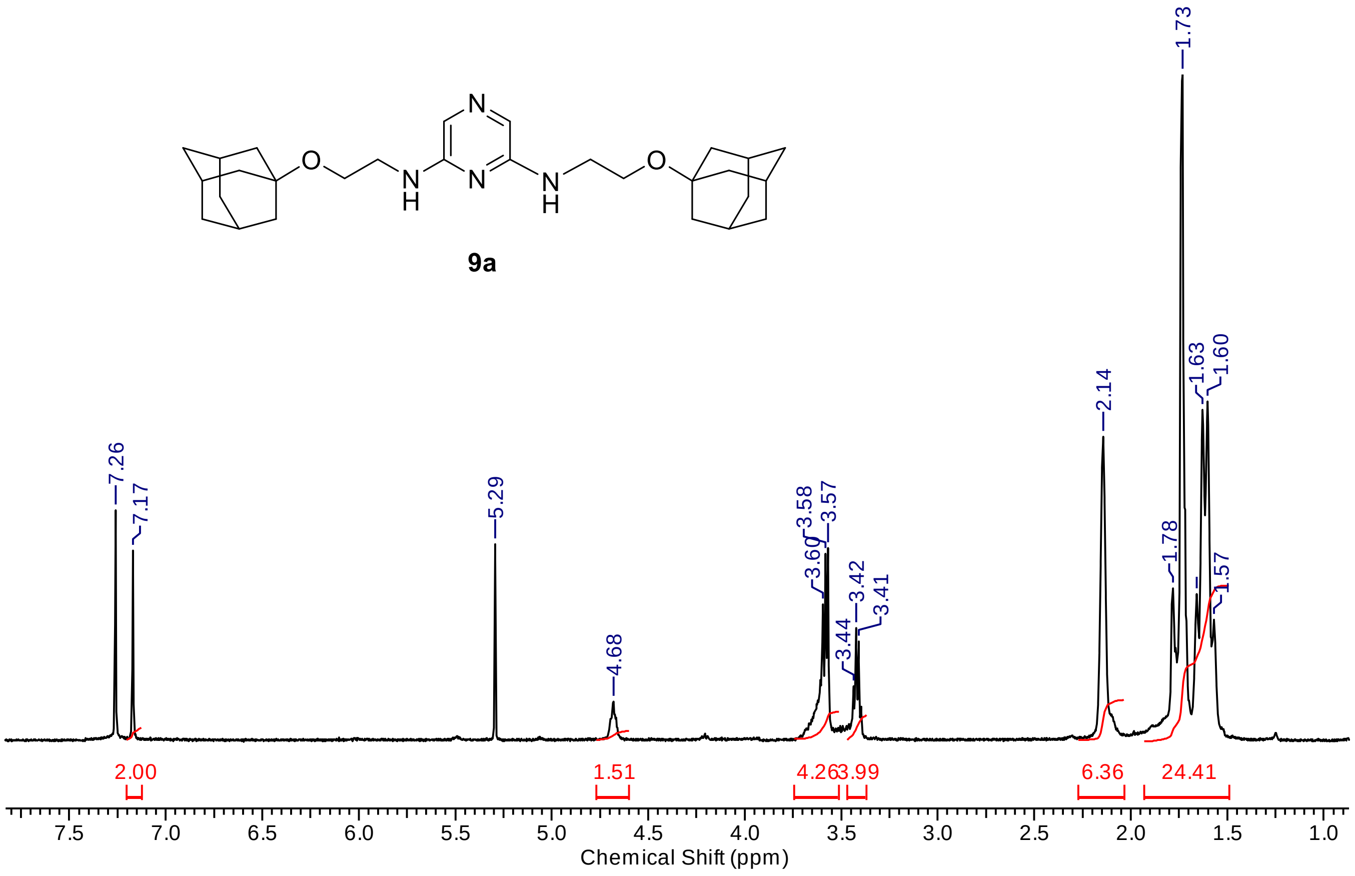

Figure S25. ^{1}H NMR spectrum of **9a** ($CDCl_3$, 400MHz, 300K).

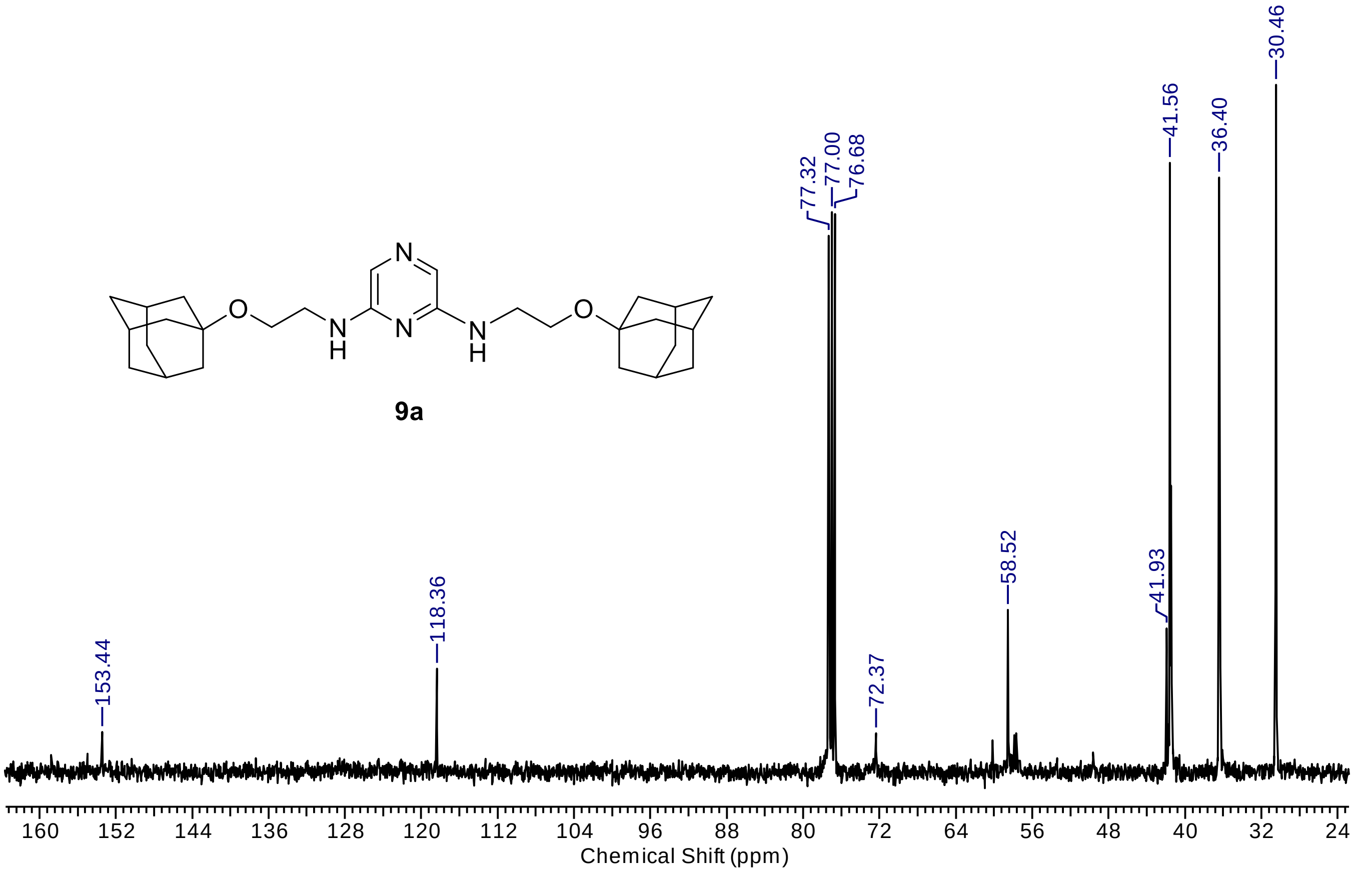

Figure S26. ^{13}C NMR spectrum of **9a** ($CDCl_3$, 100.6 MHz, 300K).

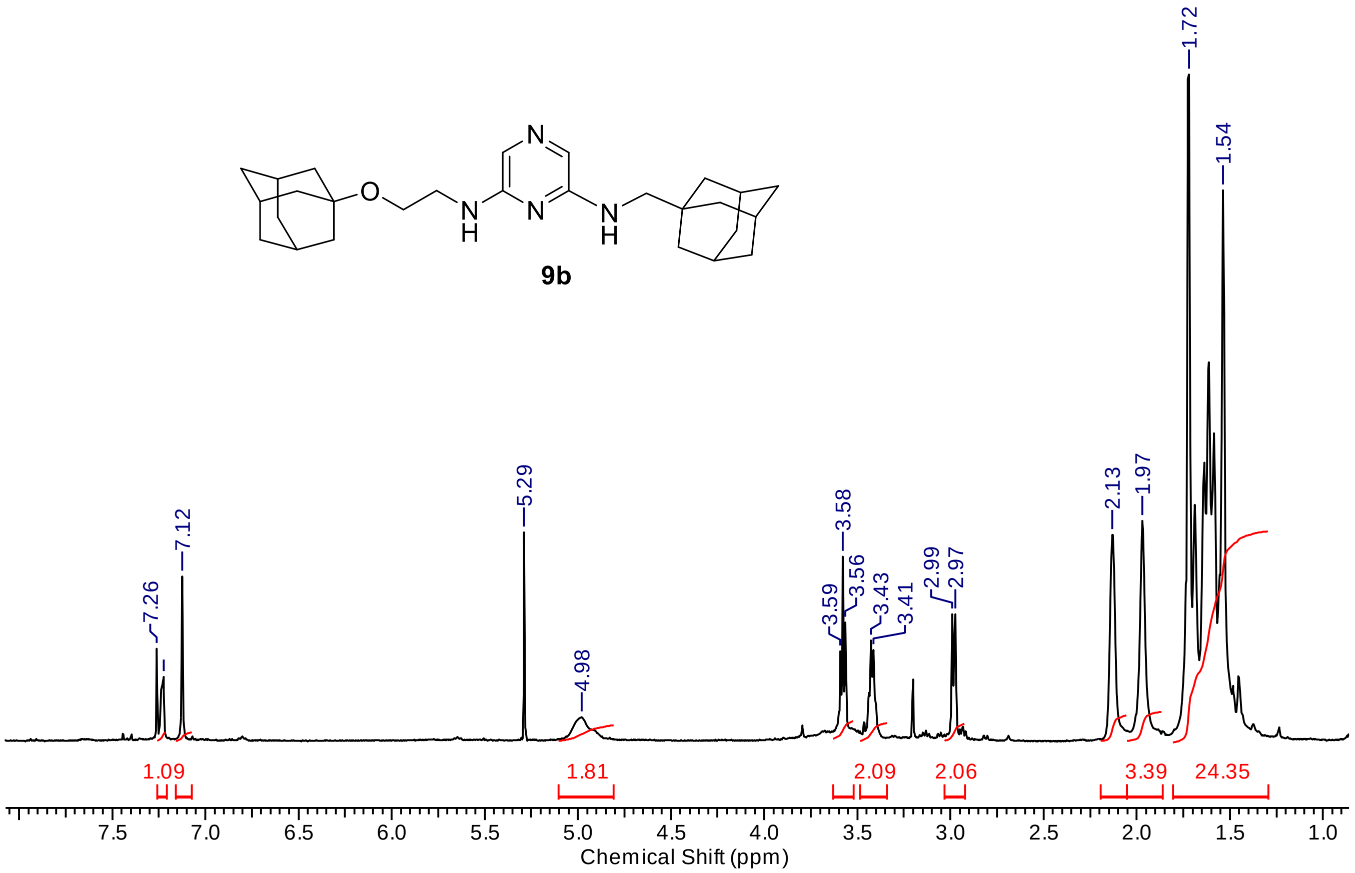

Figure S27. ^{1}H NMR spectrum of **9b** ($CDCl_3$, 400MHz, 300K).

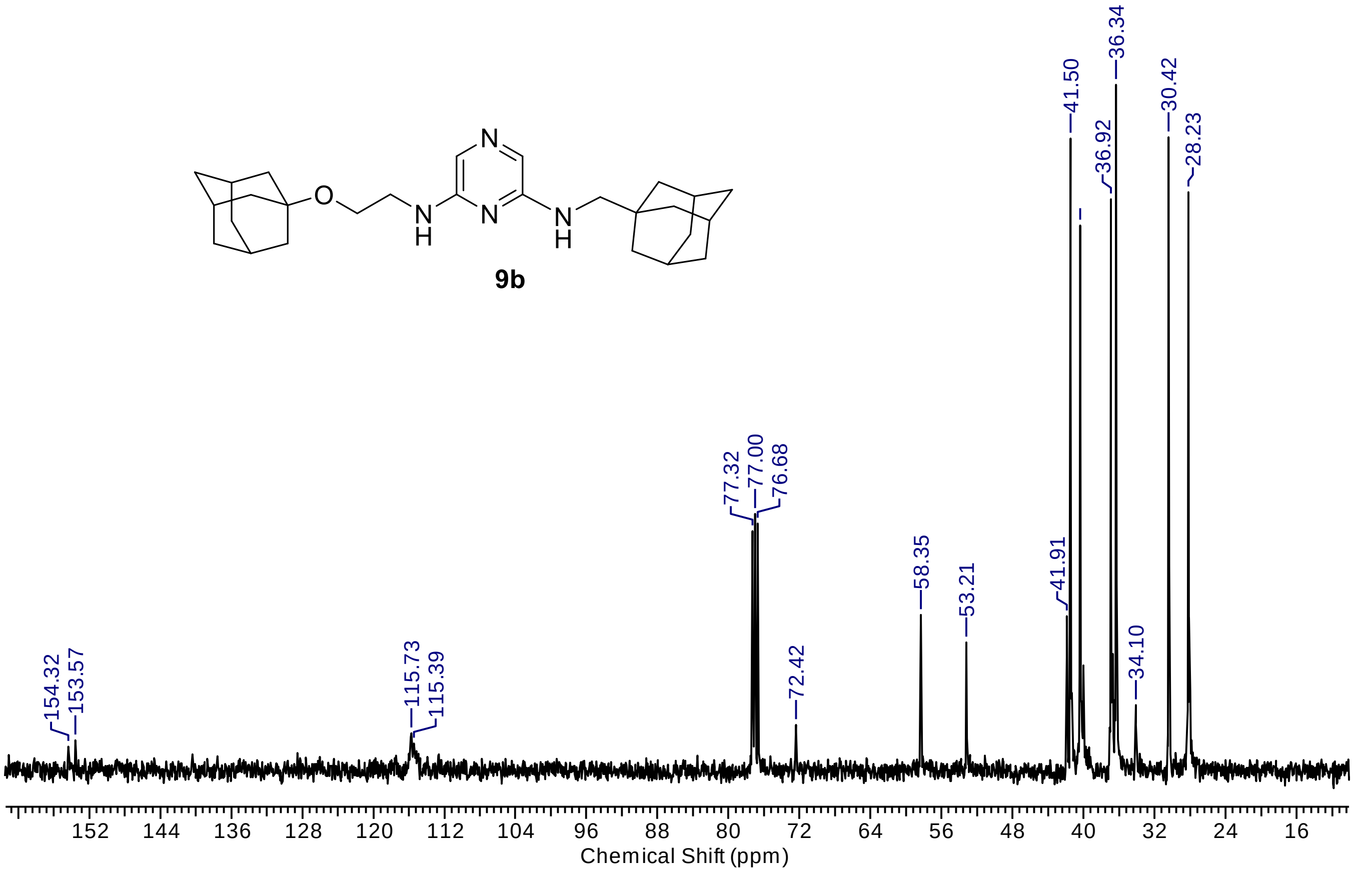

Figure S28. ^{13}C NMR spectrum of **9b** ($CDCl_3$, 100.6 MHz, 300K).

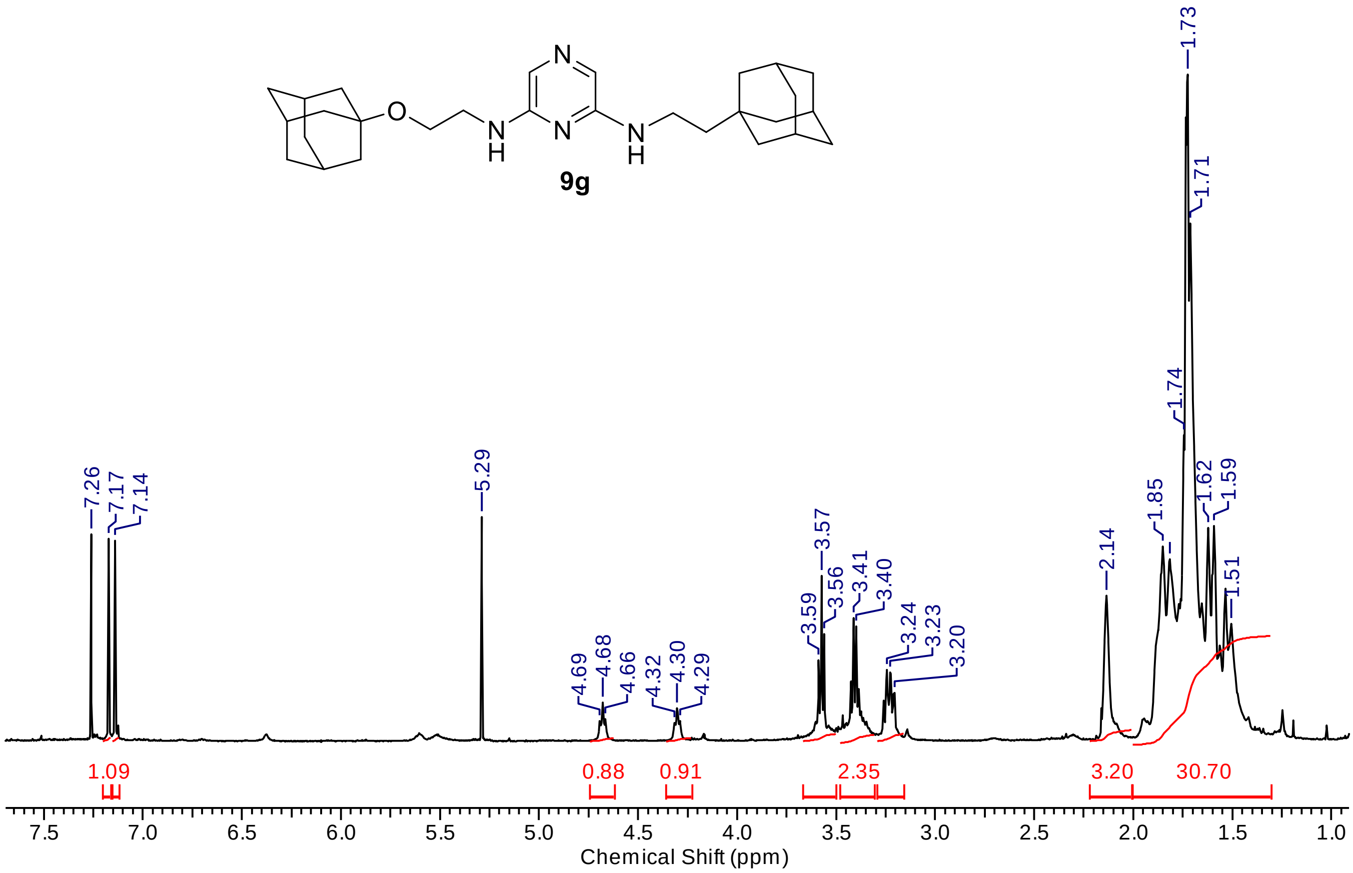

Figure S29. ^{1}H NMR spectrum of **9g** ($CDCl_3$, 400MHz, 300K).

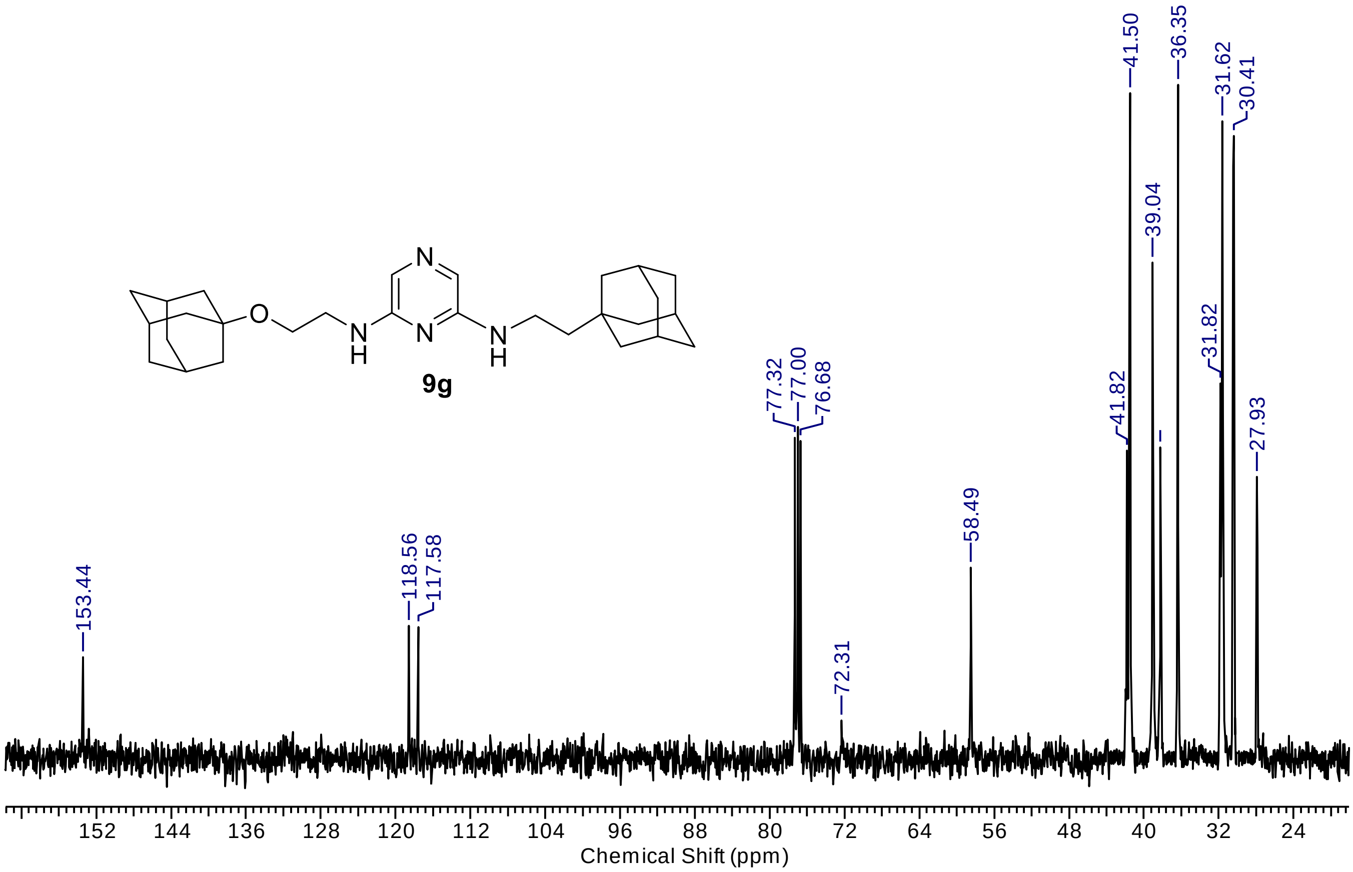

Figure S30. ^{13}C NMR spectrum of **9g** ($CDCl_3$, 100.6 MHz, 300K).

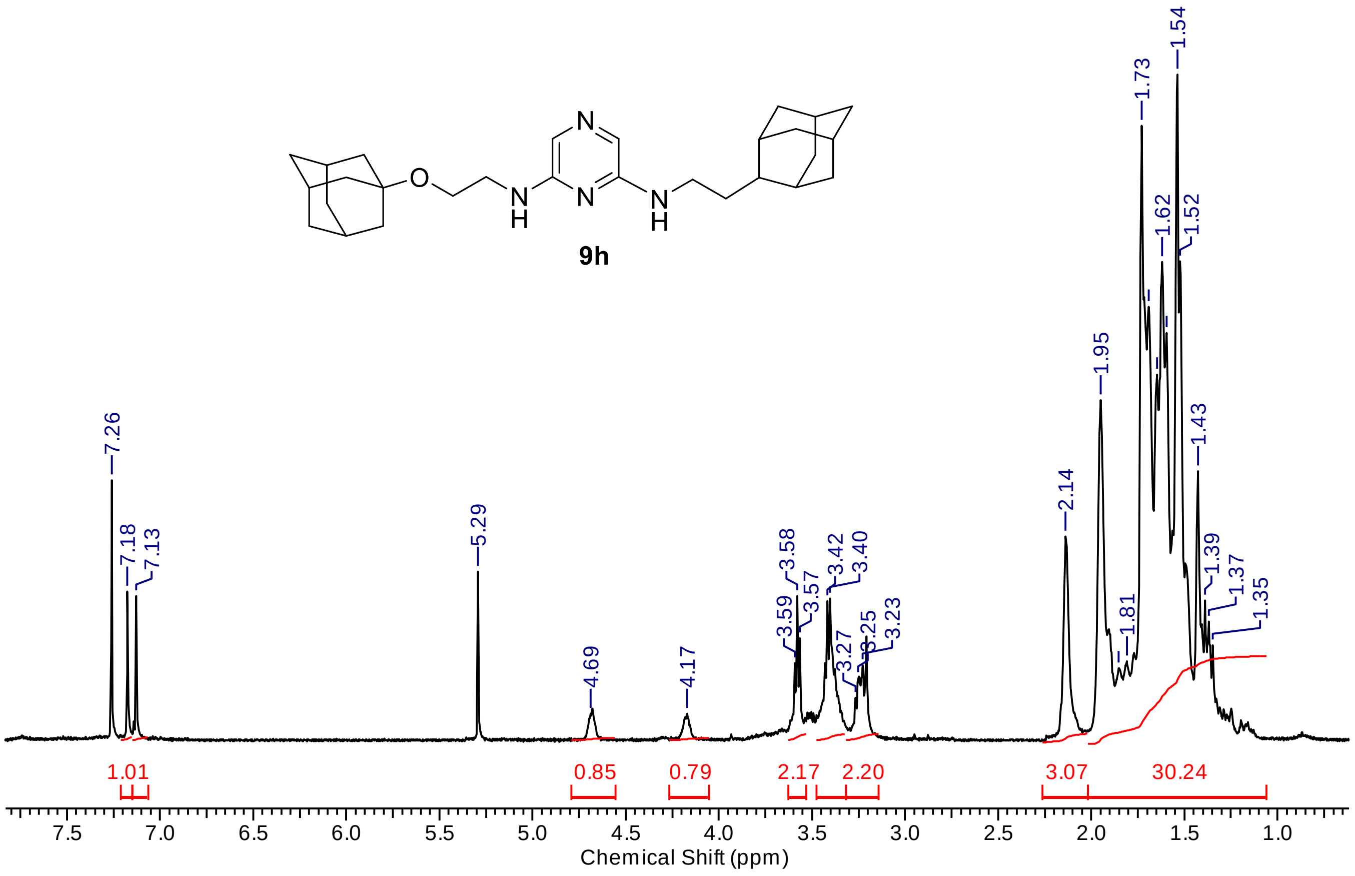

Figure S31. ^{1}H NMR spectrum of **9h** ($CDCl_3$, 400MHz, 300K).

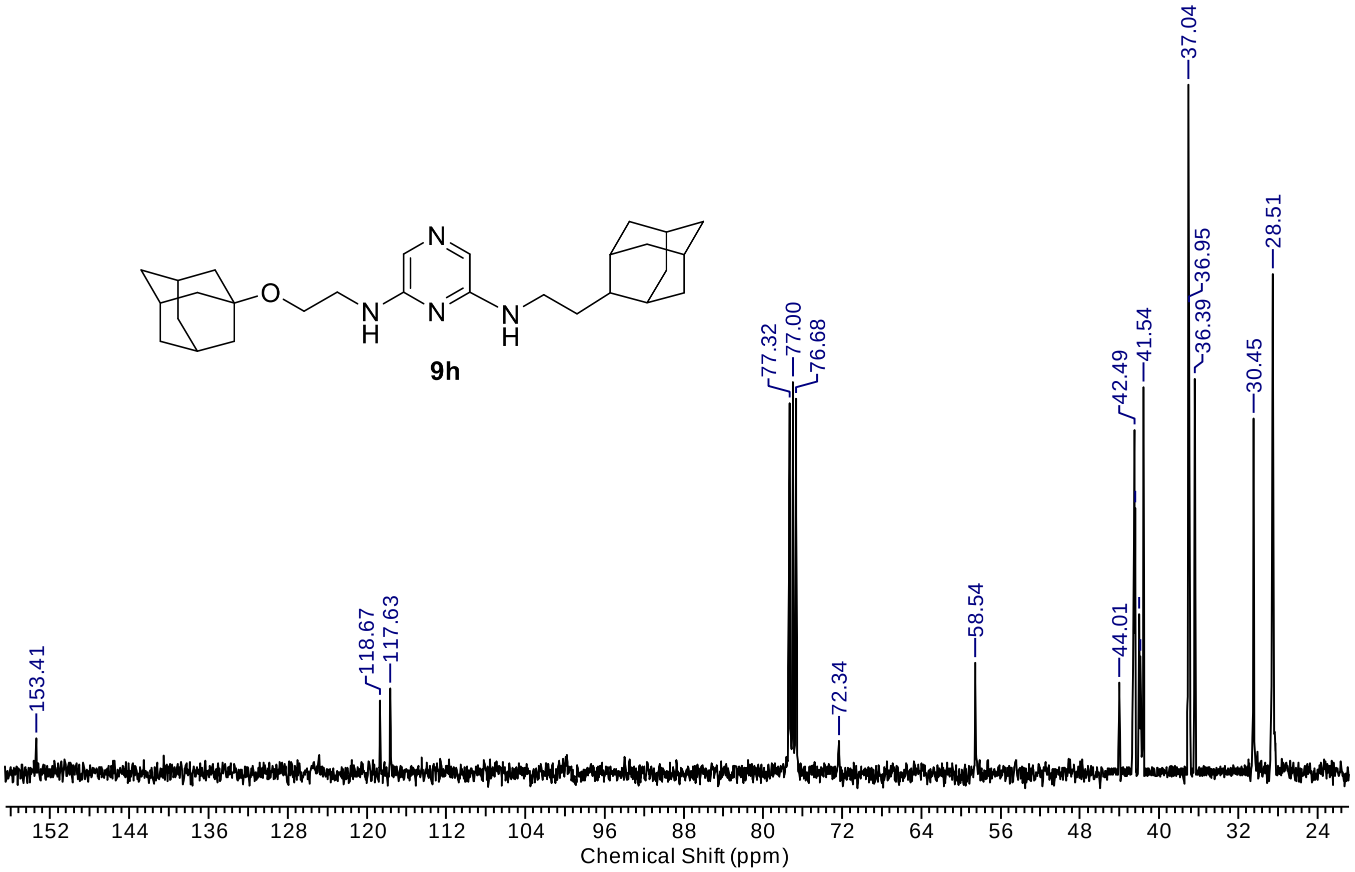

Figure S32. ^{13}C NMR spectrum of **9h** ($CDCl_3$, 100.6 MHz, 300K).

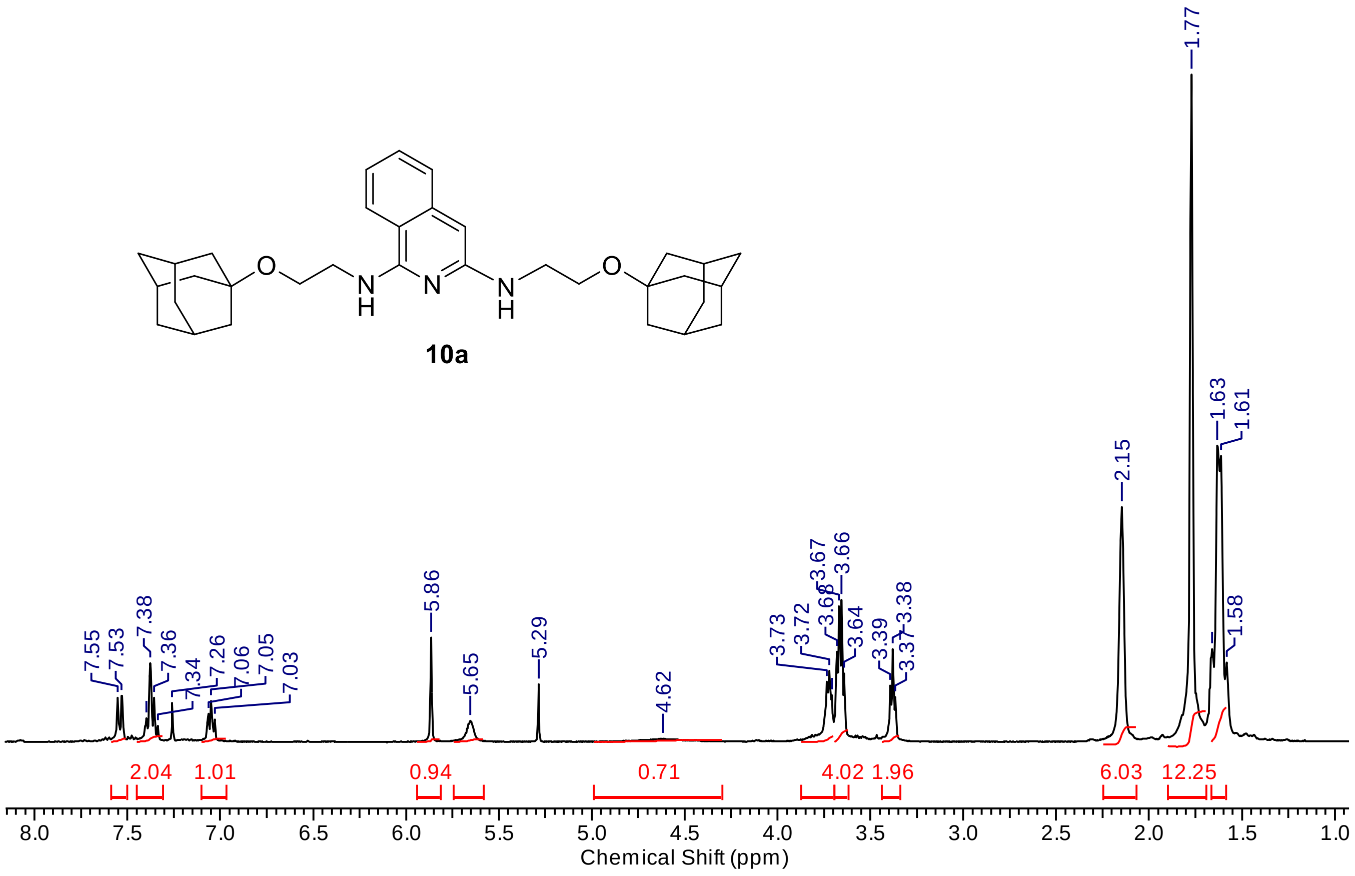

Figure S33. ^{1}H NMR spectrum of **10a** ($CDCl_3$, 400MHz, 300K).

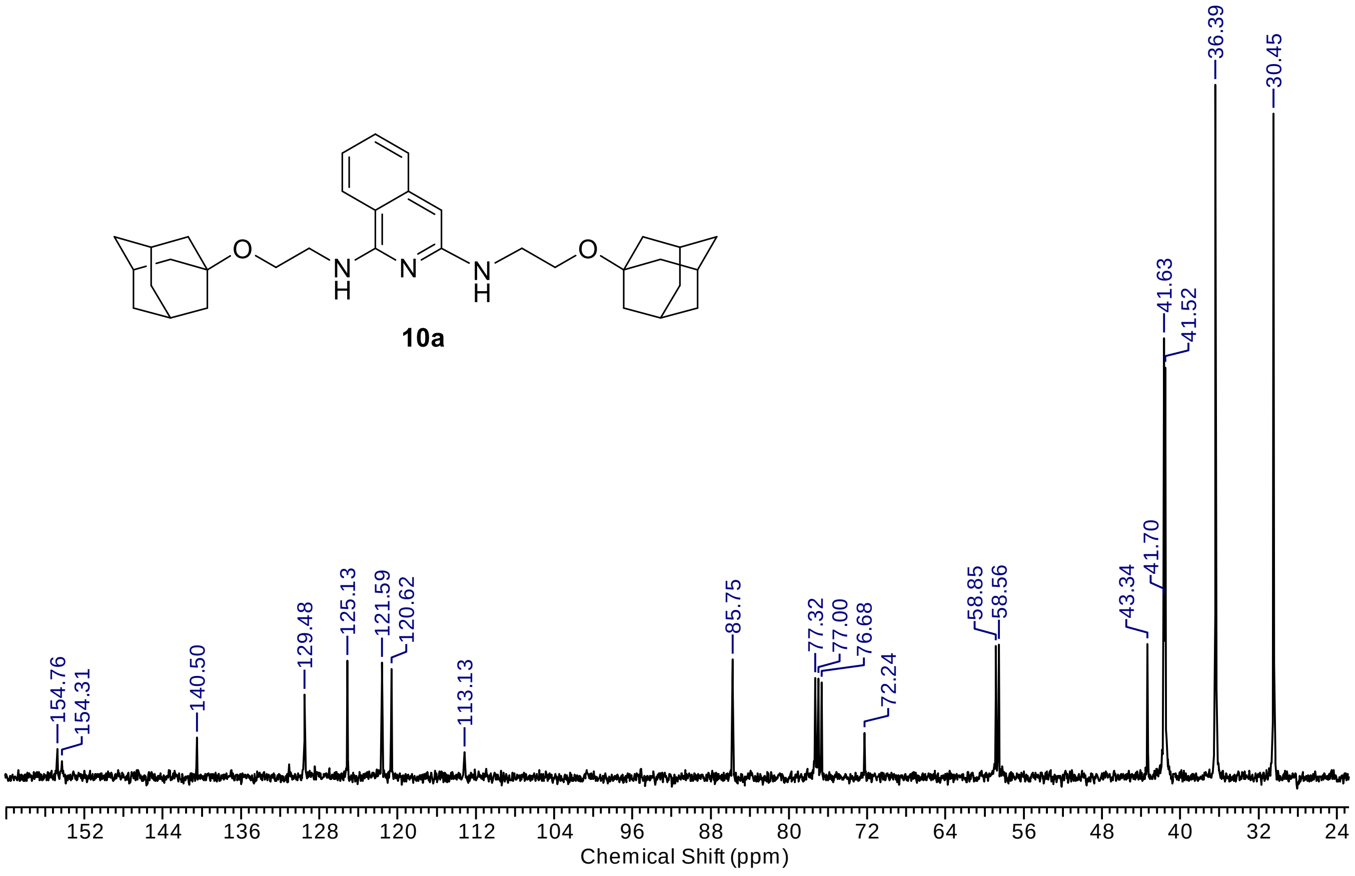

Figure S34. ^{13}C NMR spectrum of **10a** ($CDCl_3$, 100.6 MHz, 300K).

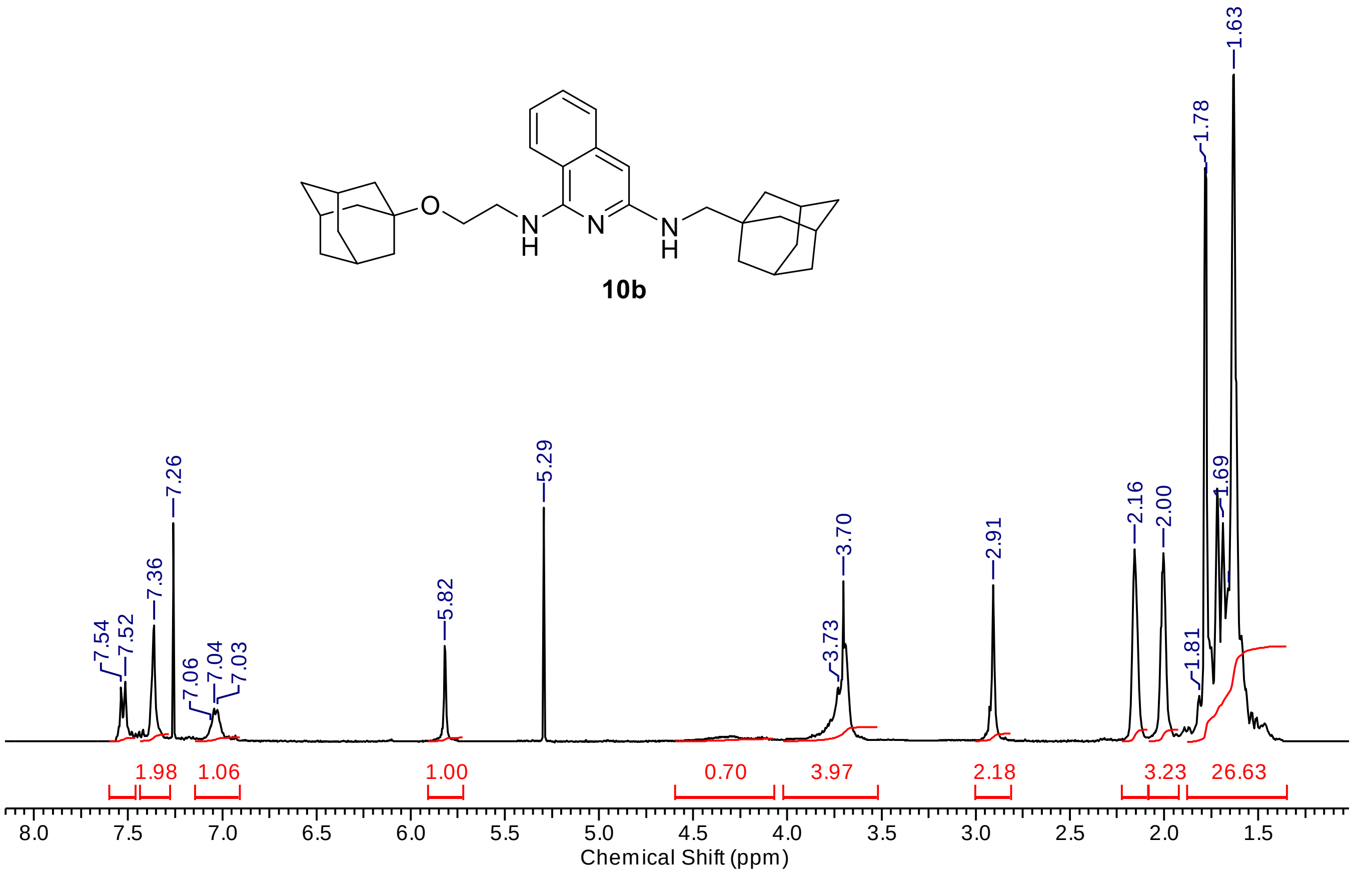

Figure S35. ^{1}H NMR spectrum of **10b** ($CDCl_3$, 400MHz, 300K).

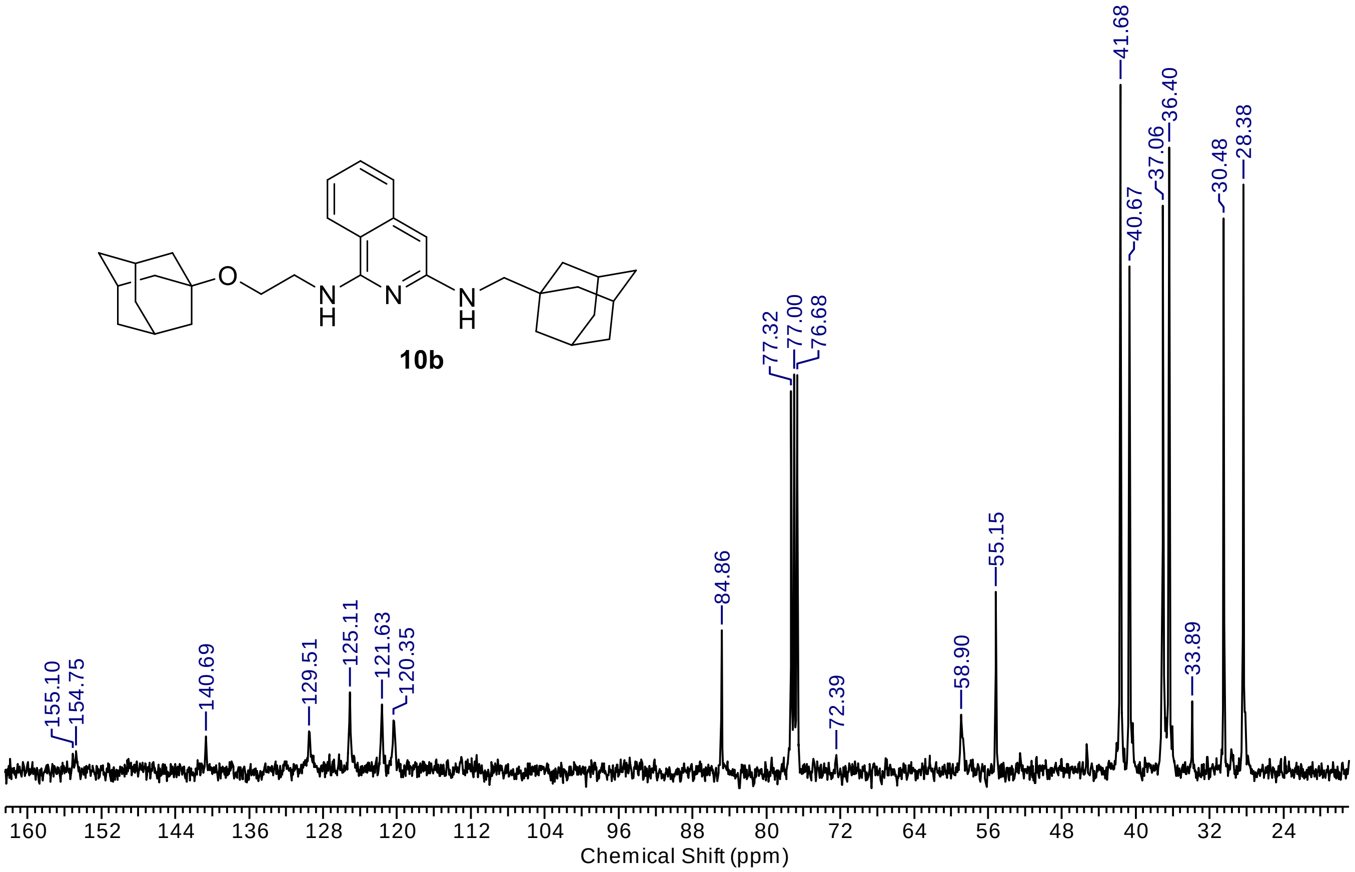

Figure S36. ^{13}C NMR spectrum of **10b** ($CDCl_3$, 100.6 MHz, 300K).

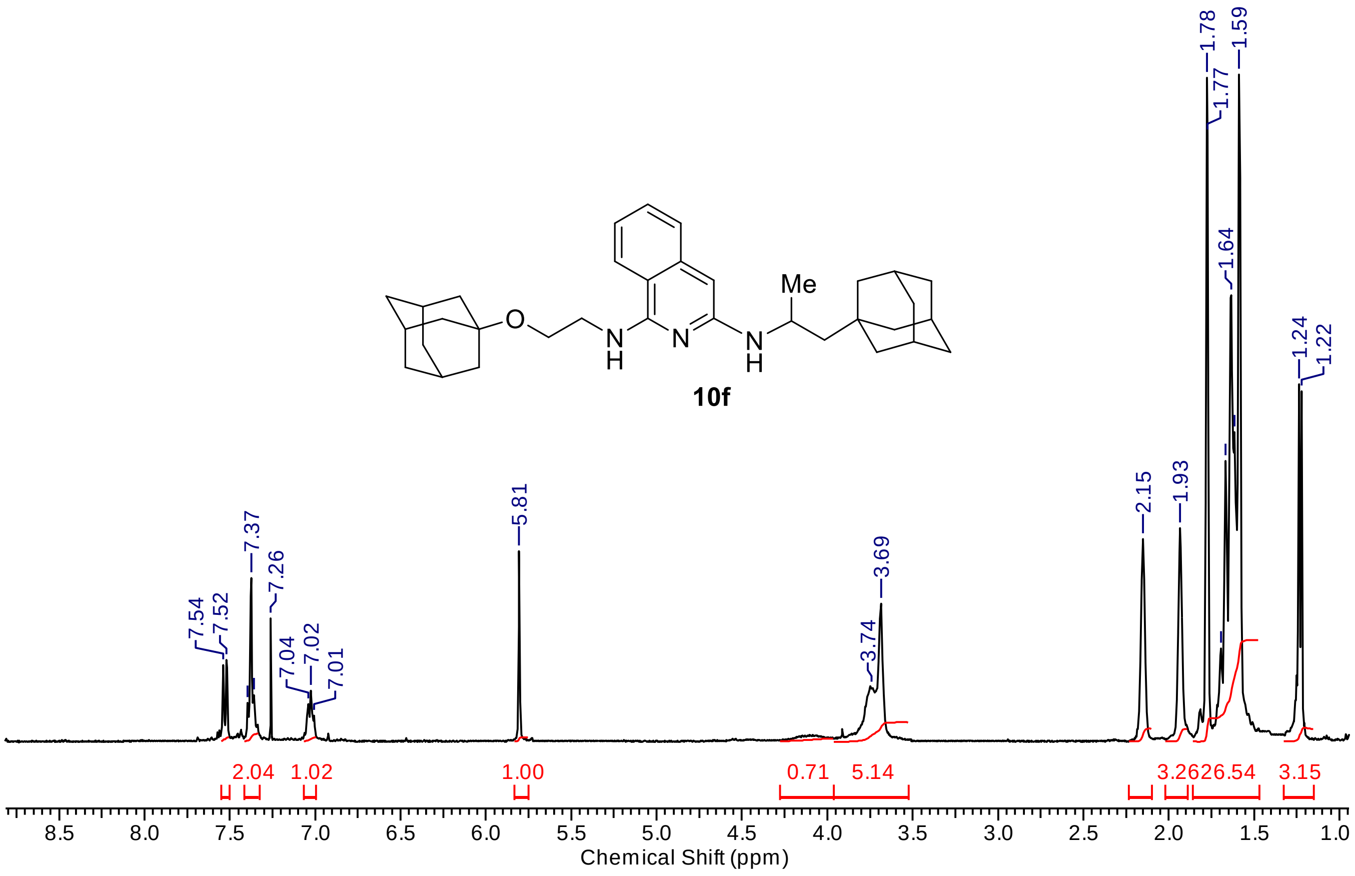

Figure S37. ^{1}H NMR spectrum of **10f** ($CDCl_3$, 400MHz, 300K).

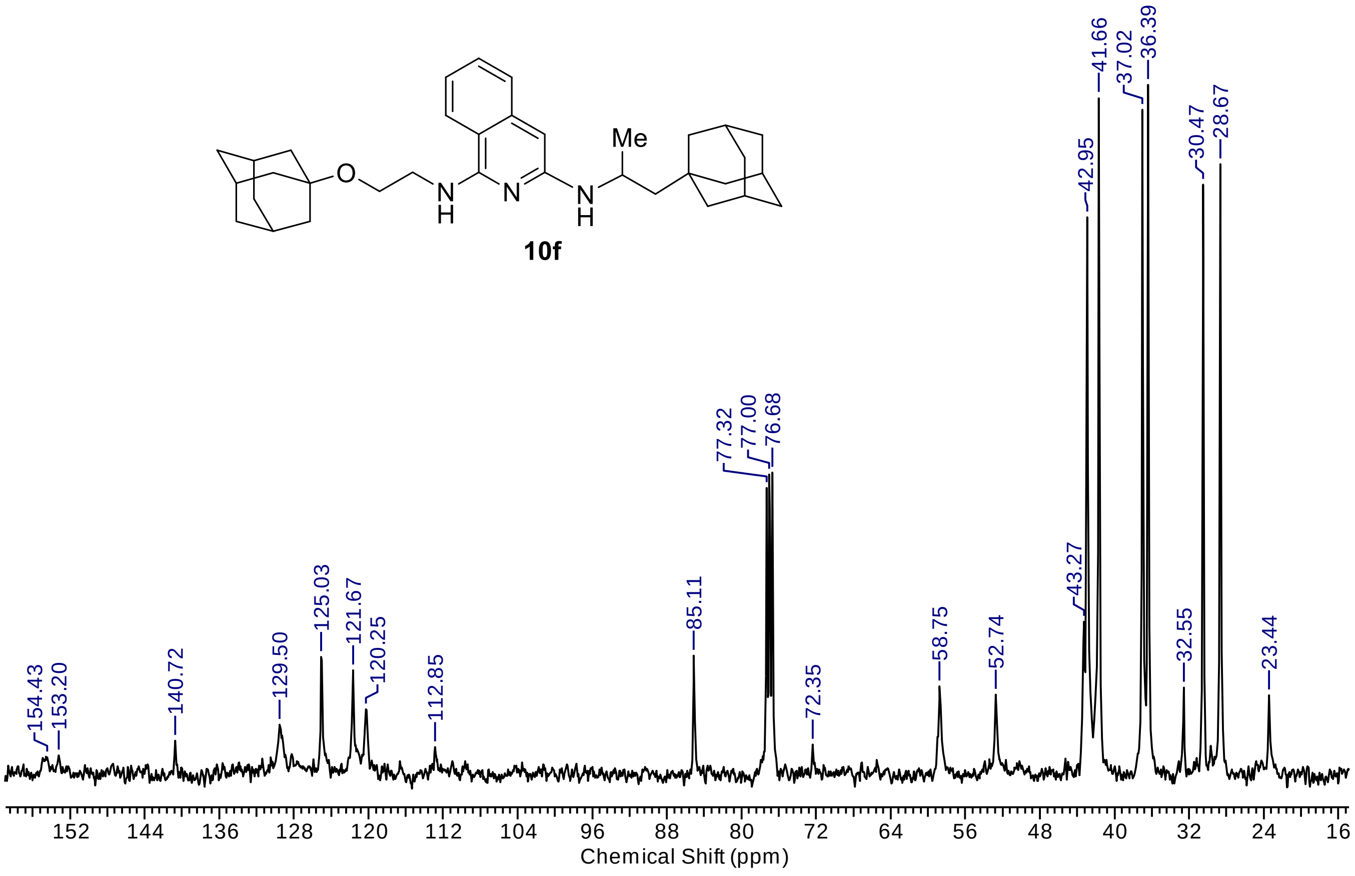

Figure S38. ^{13}C NMR spectrum of **10f** ($CDCl_3$, 100.6 MHz, 300K).

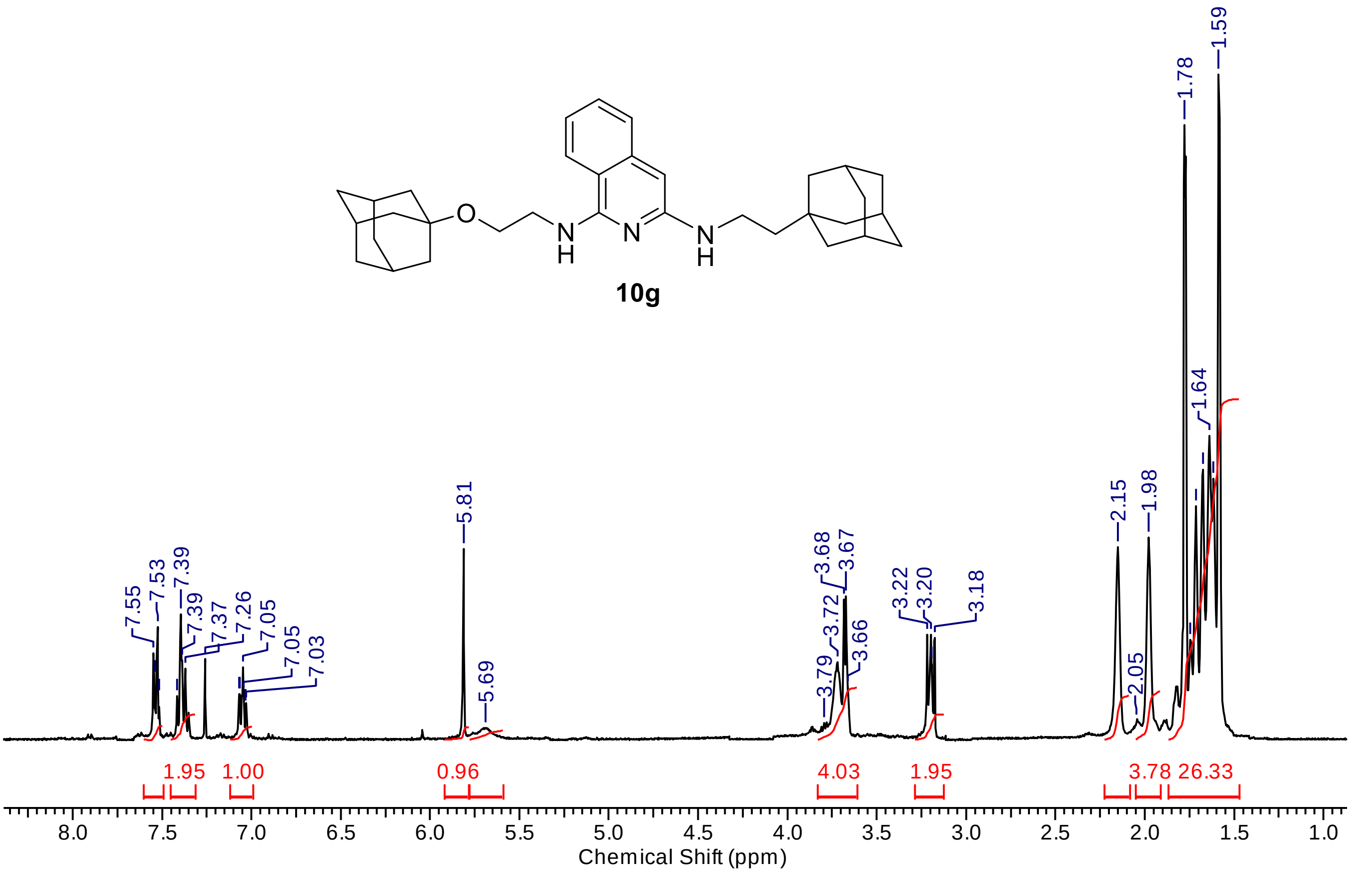

Figure S39. ^{1}H NMR spectrum of **10g** ($CDCl_3$, 400MHz, 300K).

MALDI-TOF spectra

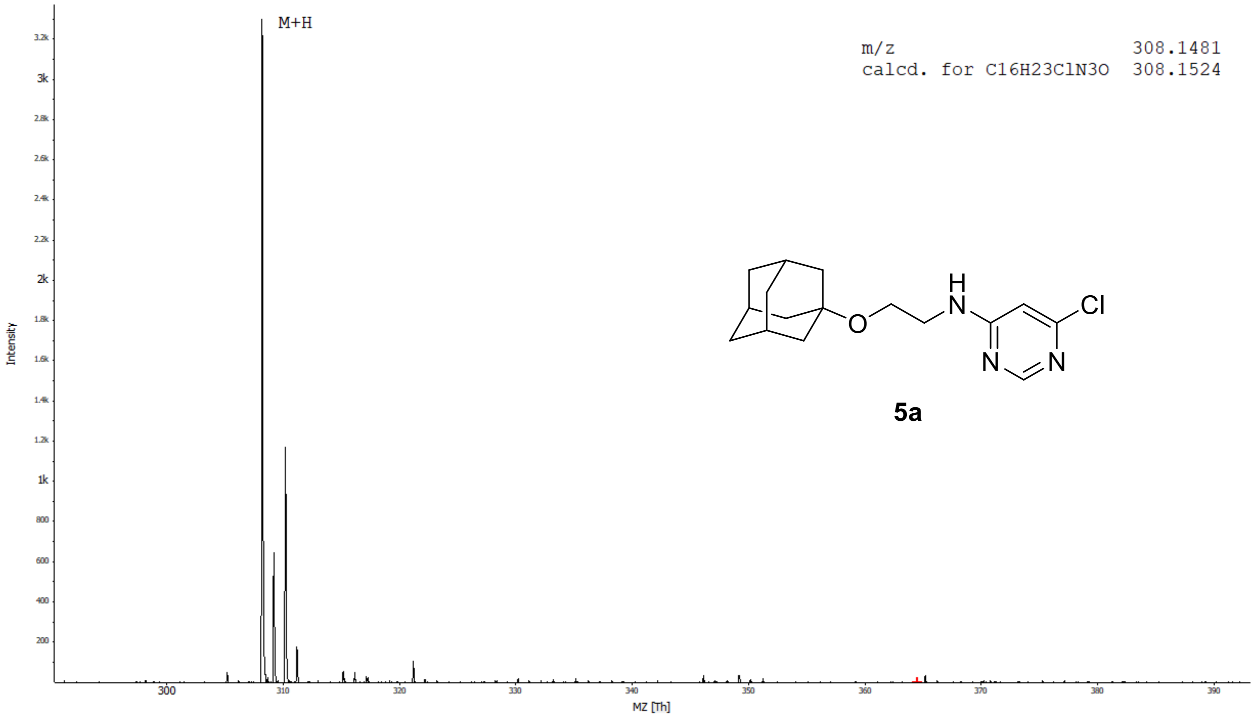

Figure S40. MALDI-TOF spectra of the compound **5a**. Matrix: 1,8,9-trihydroxyanthracene. Calibration standard: PEG-300.

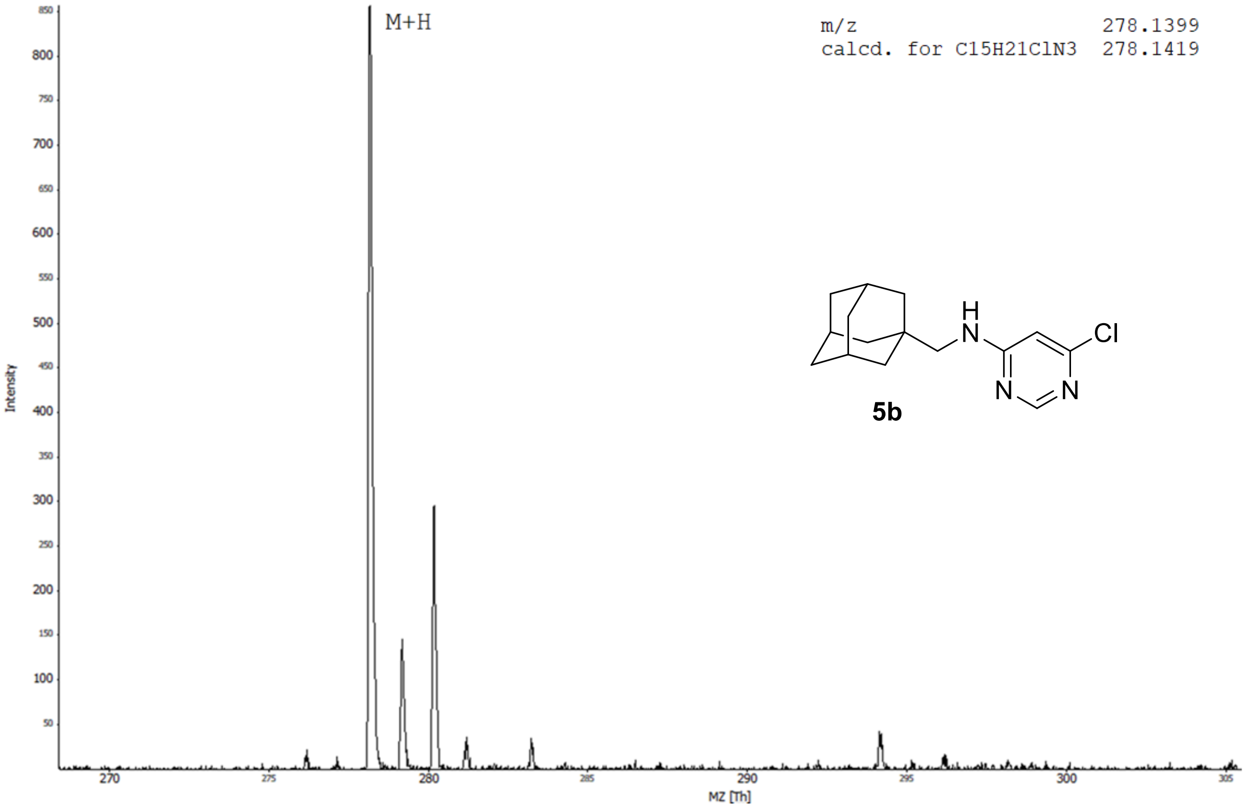

Figure S41. MALDI-TOF spectra of the compound **5b**. Matrix: 1,8,9-trihydroxyanthracene. Calibration standard: PEG-300.

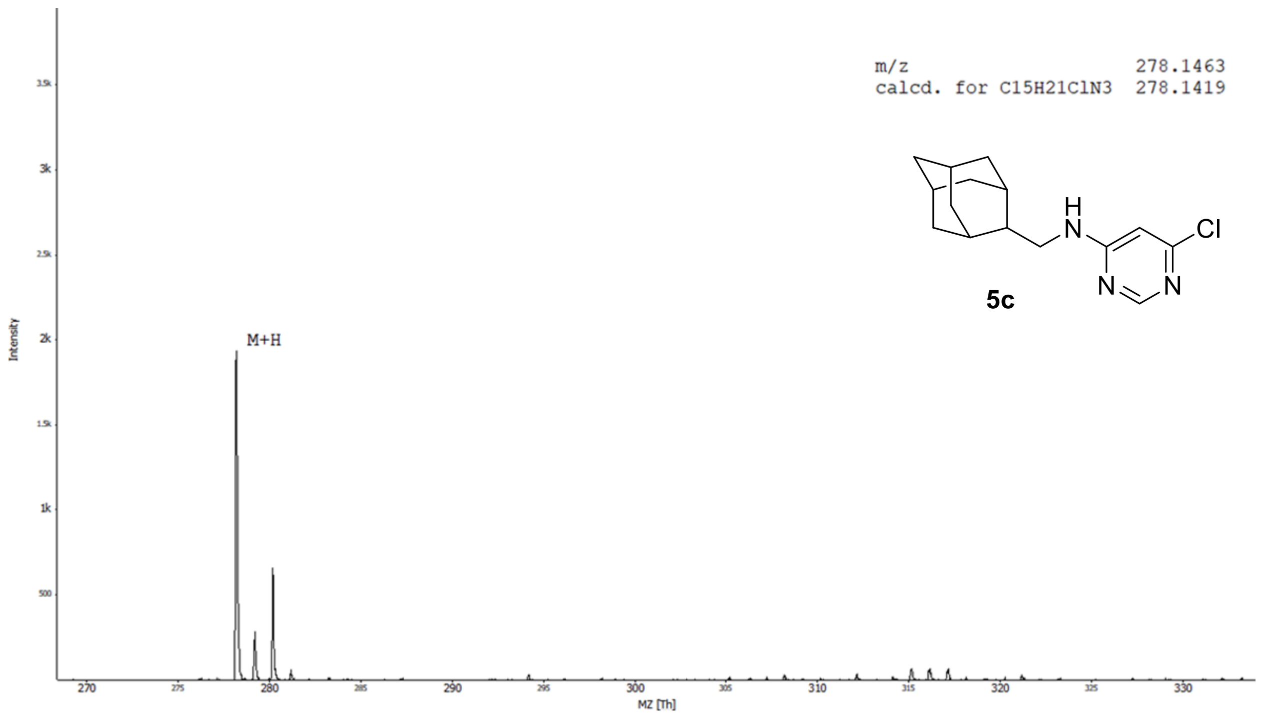

Figure S42. MALDI-TOF spectra of the compound **5c**. Matrix: 1,8,9-trihydroxyanthracene. Calibration standard: PEG-300.

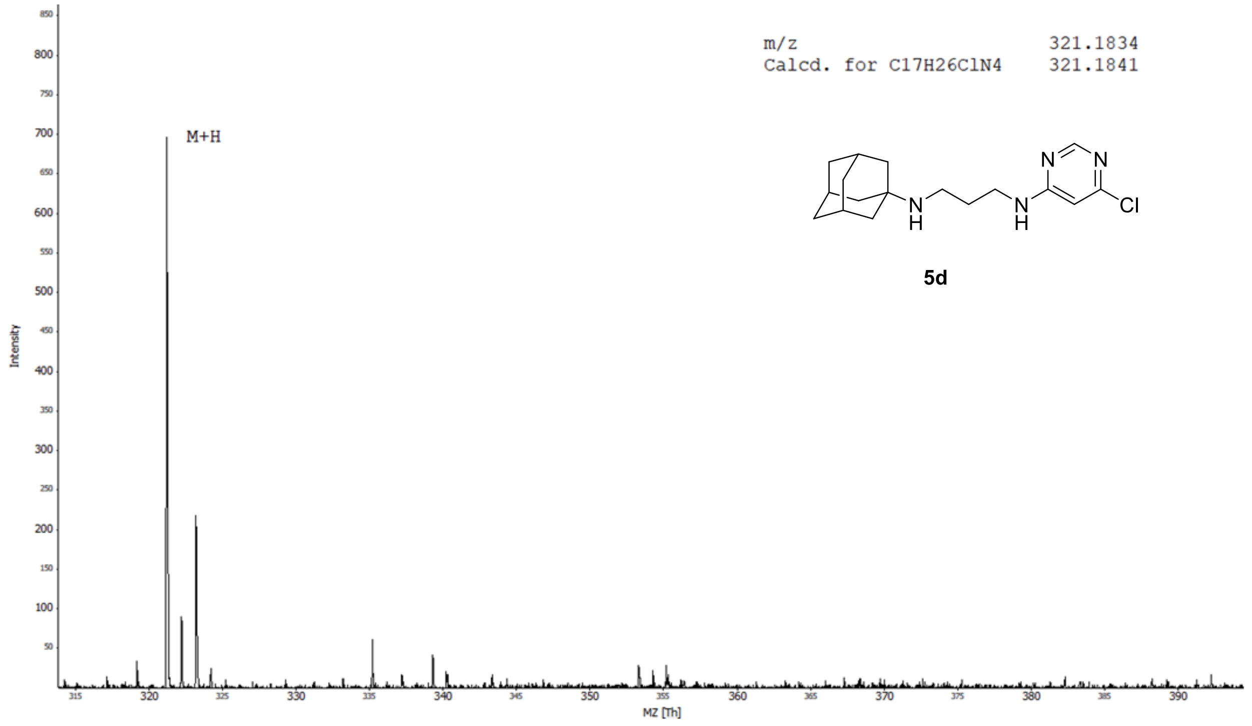

Figure S43. MALDI-TOF spectra of the compound **5d**. Matrix: 1,8,9-trihydroxyanthracene. Calibration standard: PEG-300.

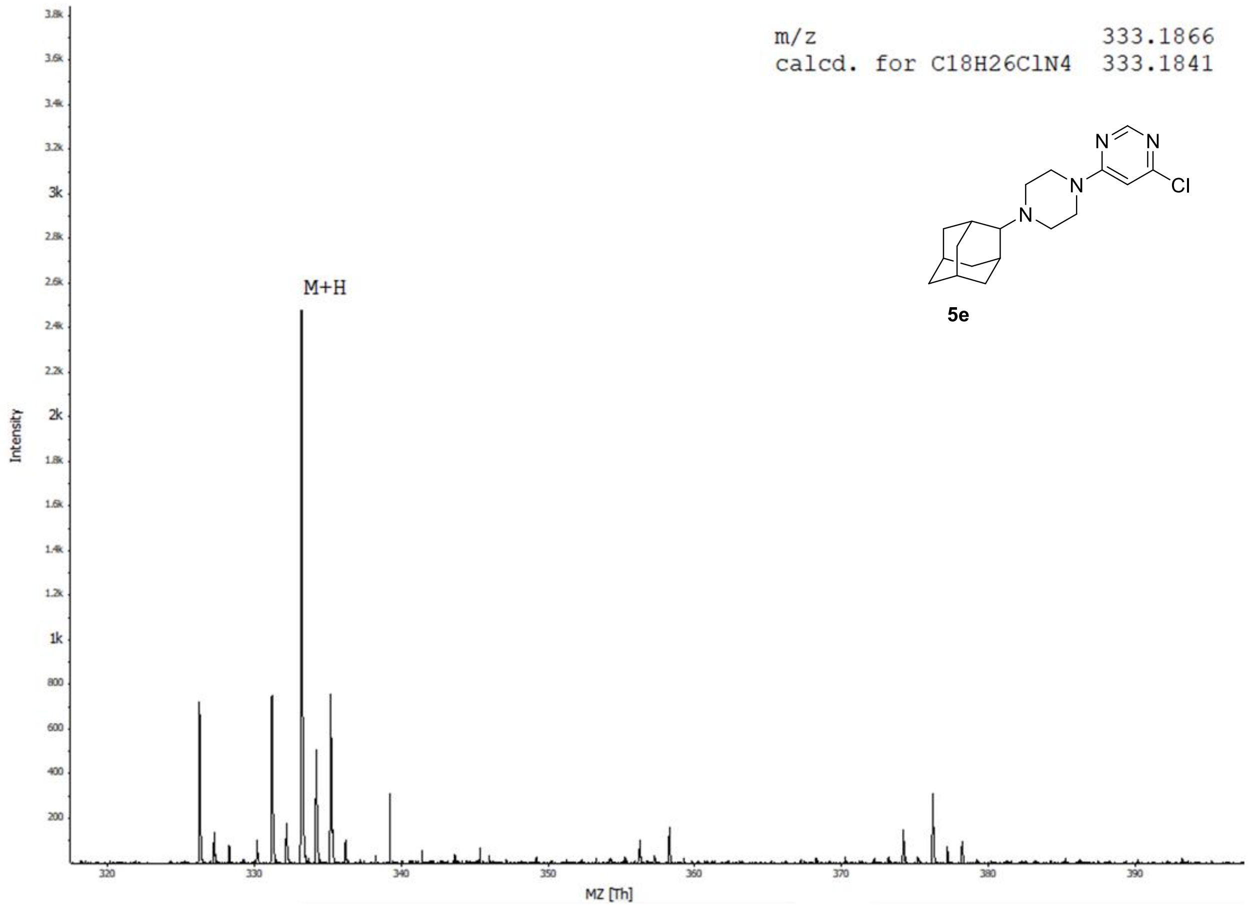

Figure S44. MALDI-TOF spectra of the compound **5e**. Matrix: 1,8,9-trihydroxyanthracene. Calibration standard: PEG-300.

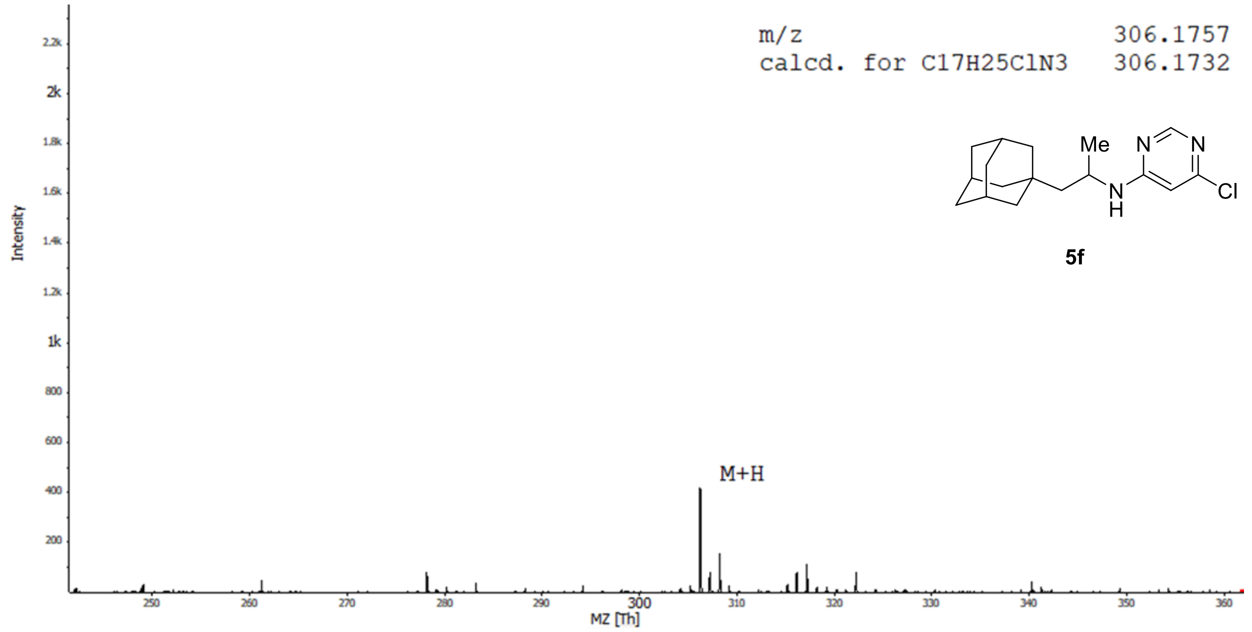

Figure S45. MALDI-TOF spectra of the compound **5f**. Matrix: 1,8,9-trihydroxyanthracene. Calibration standard: PEG-300.

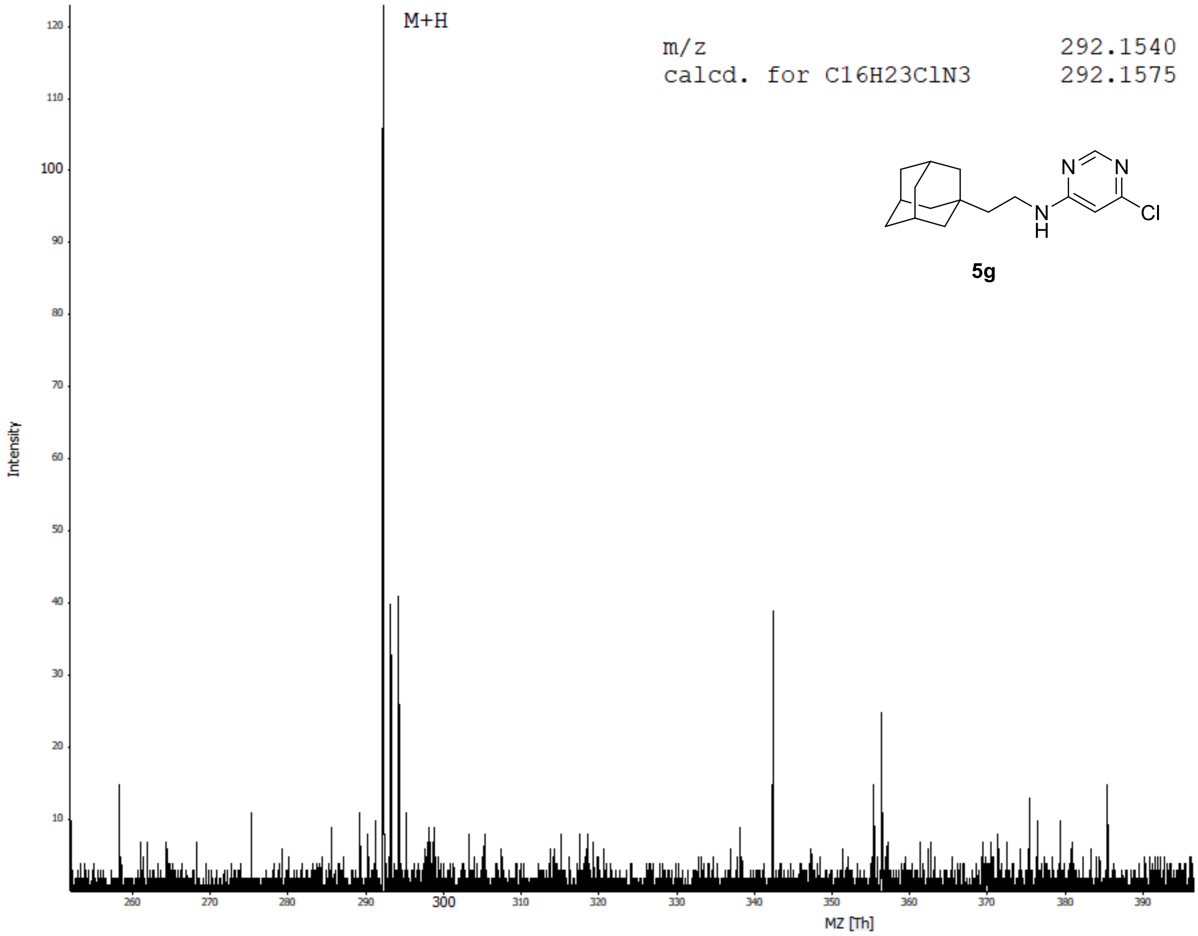

Figure S46. MALDI-TOF spectra of the compound **5g**. Matrix: 1,8,9-trihydroxyanthracene. Calibration standard: PEG-300.

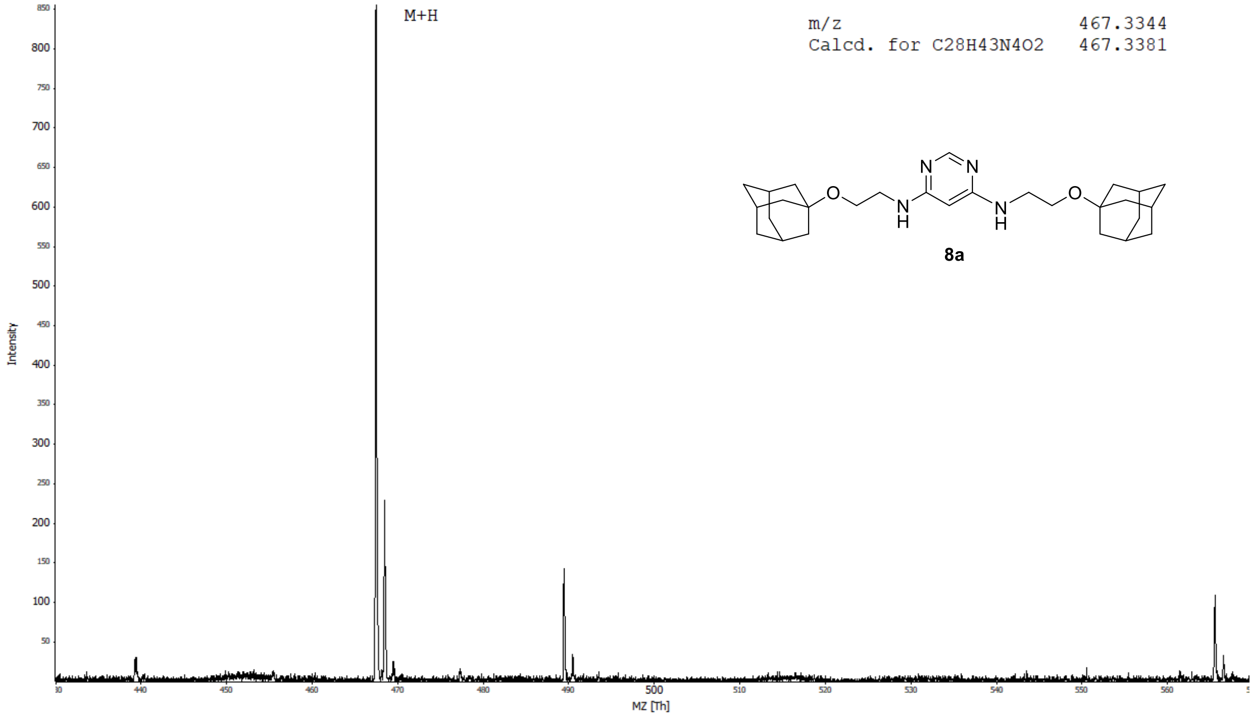

Figure S47. MALDI-TOF spectra of the compound **8a**. Matrix: 1,8,9-trihydroxyanthracene. Calibration standard: PEG-400.

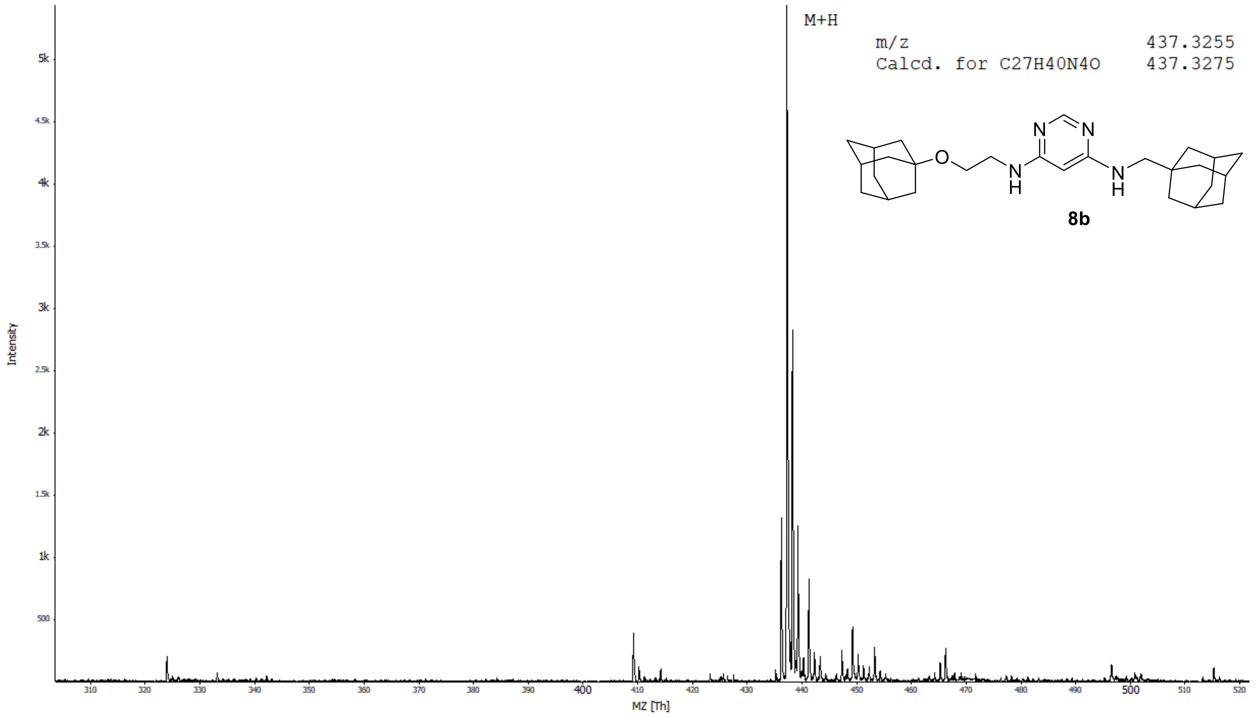

Figure S48. MALDI-TOF spectra of the compound **8b**. Matrix: 1,8,9-trihydroxyanthracene. Calibration standard: PEG-400.

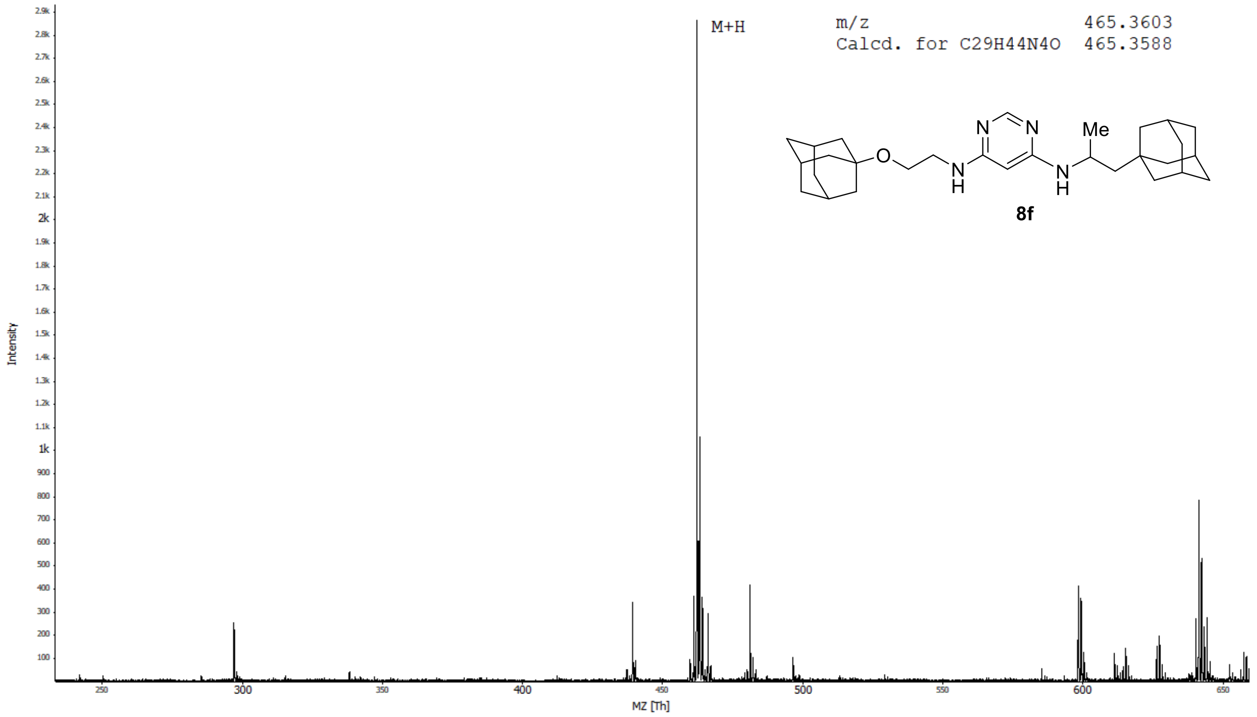

Figure S49. MALDI-TOF spectra of the compound **8f**. Matrix: 1,8,9-trihydroxyanthracene. Calibration standard: PEG-400.

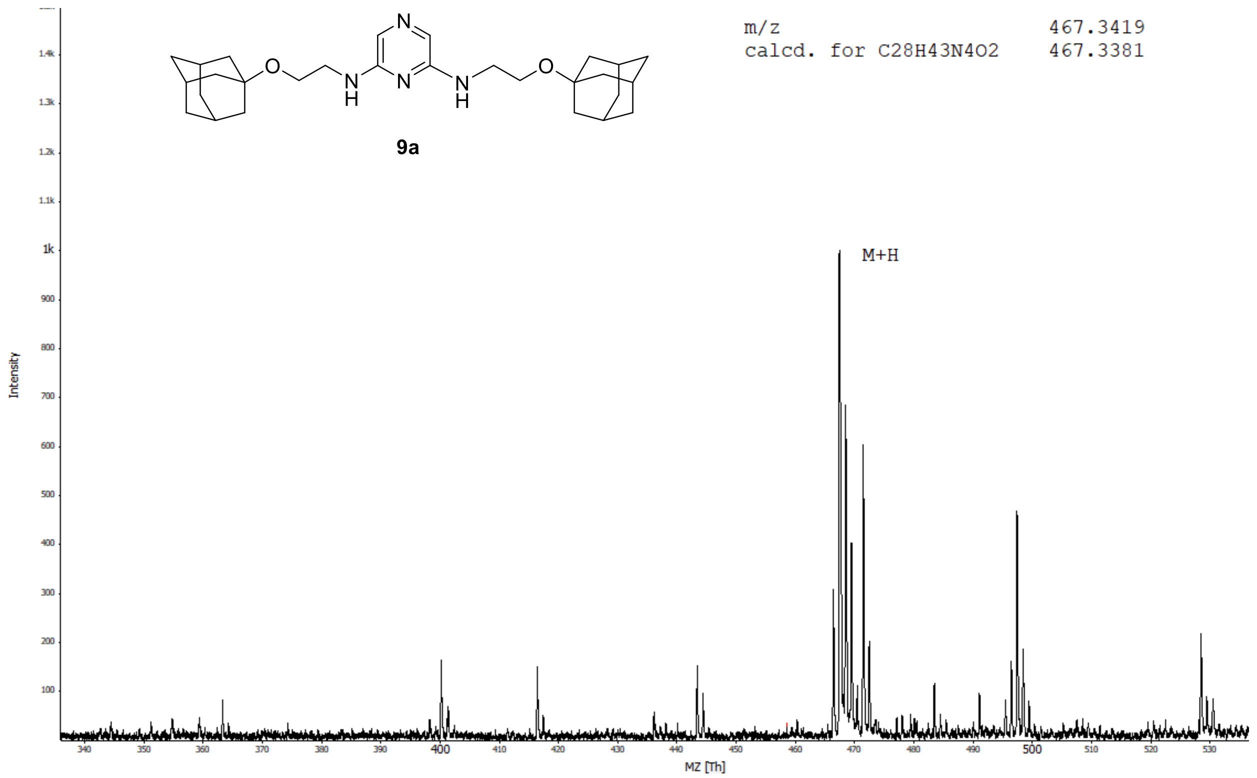

Figure S50. MALDI-TOF spectra of the compound **9a**. Matrix: 1,8,9-trihydroxyanthracene. Calibration standard: PEG-400.

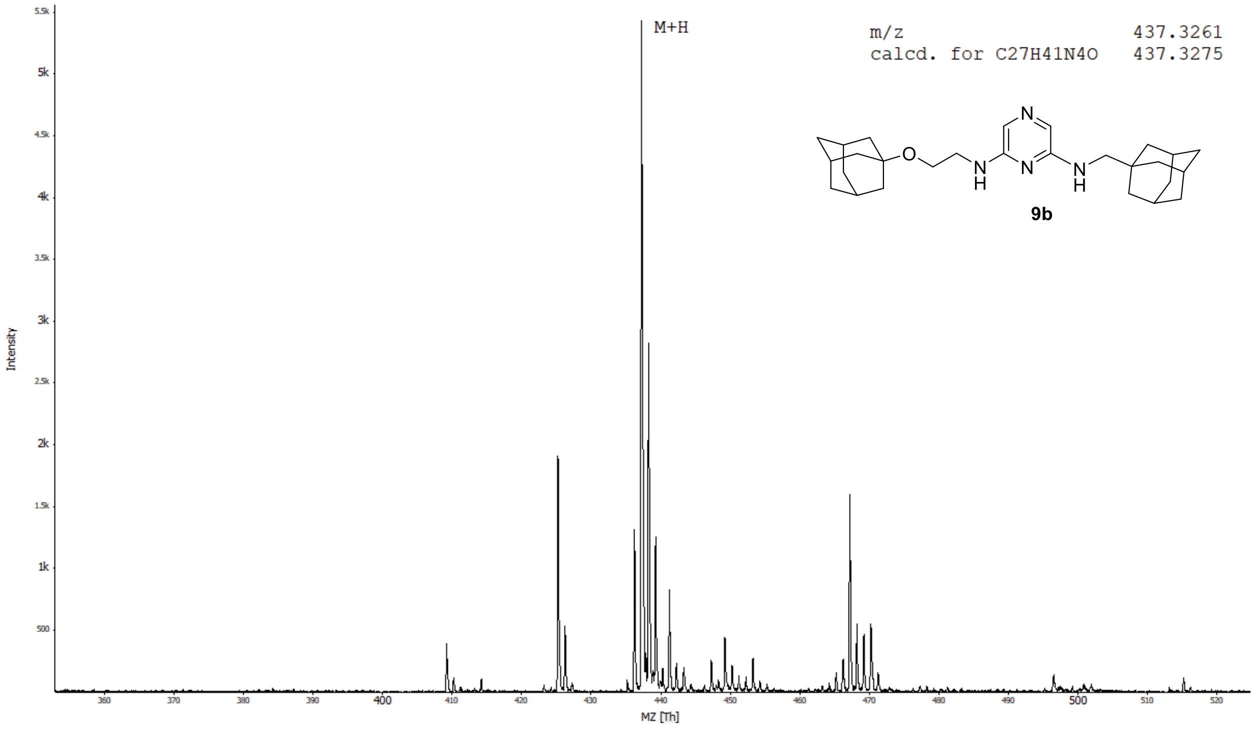

Figure S51. MALDI-TOF spectra of the compound **9b**. Matrix: 1,8,9-trihydroxyanthracene. Calibration standard: PEG-400.

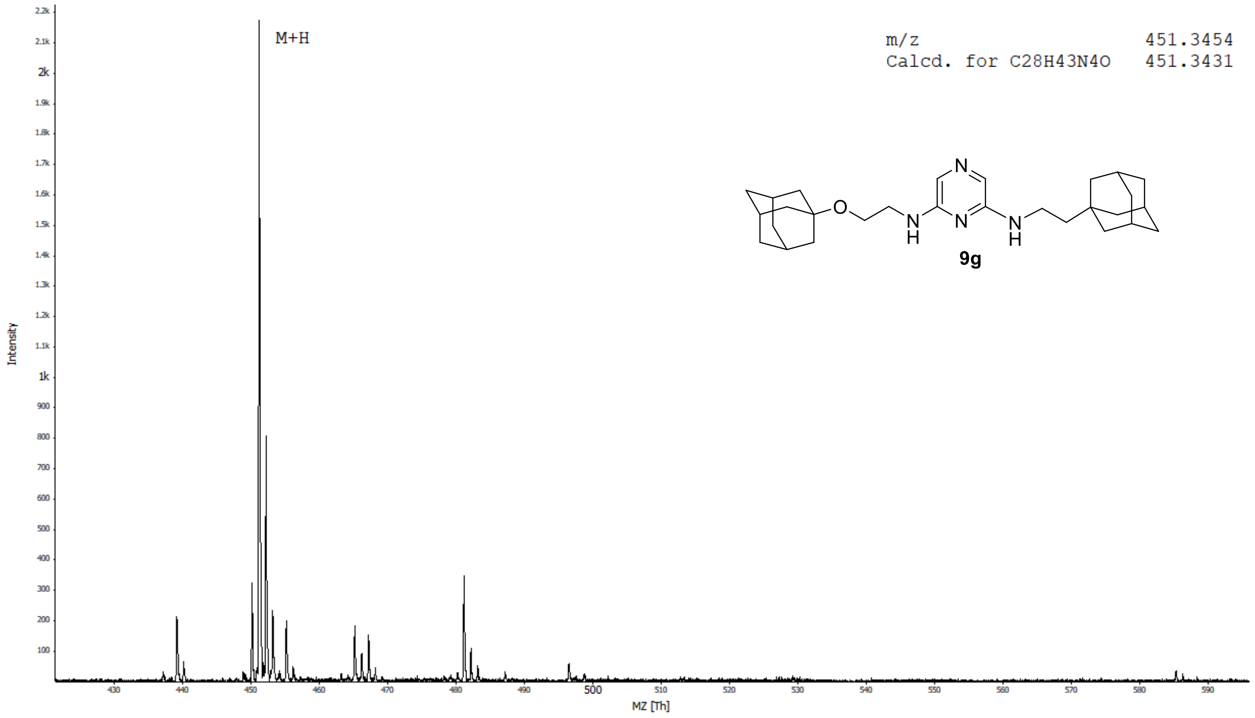

Figure S52. MALDI-TOF spectra of the compound **9g**. Matrix: 1,8,9-trihydroxyanthracene. Calibration standard: PEG-400.

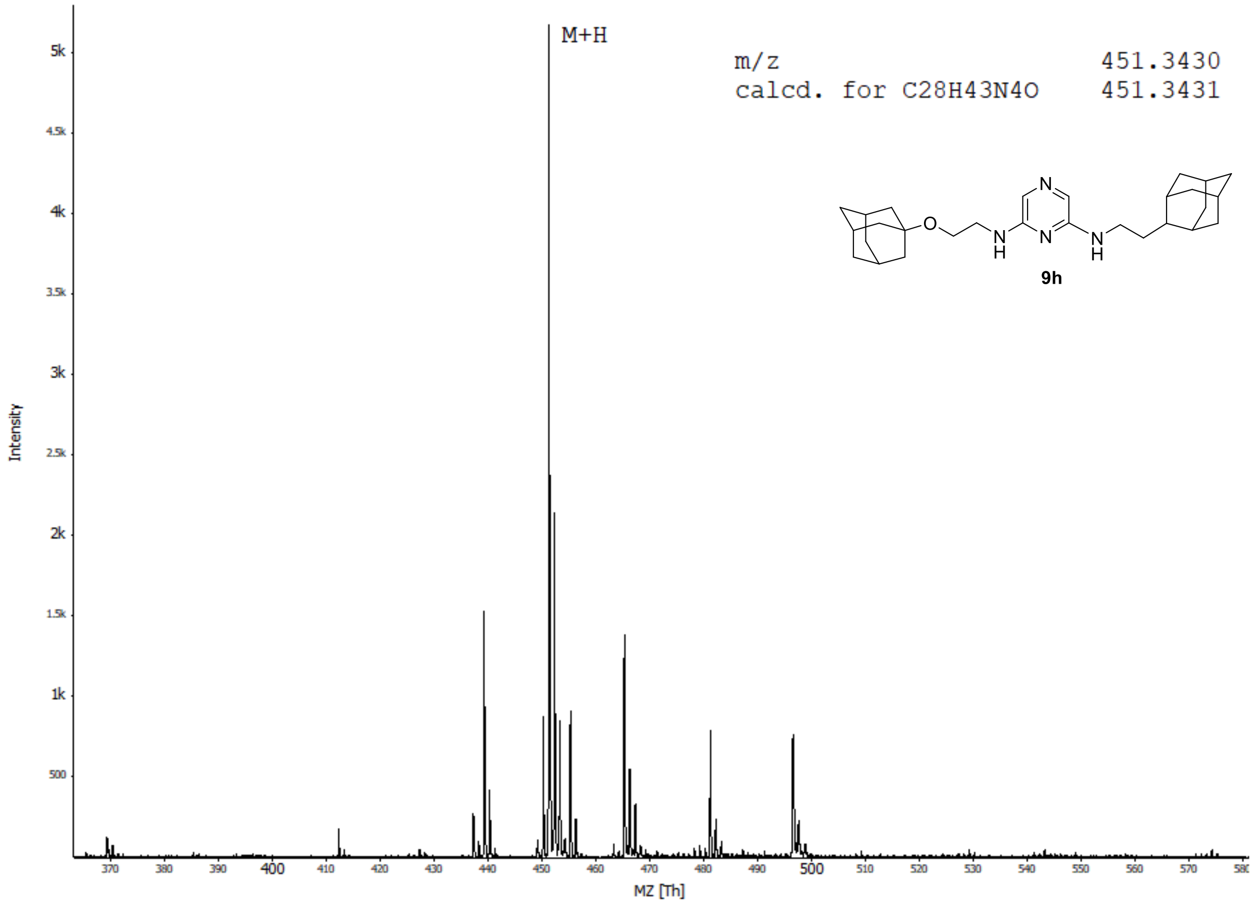

Figure S53. MALDI-TOF spectra of the compound **9h**. Matrix: 1,8,9-trihydroxyanthracene. Calibration standard: PEG-400.

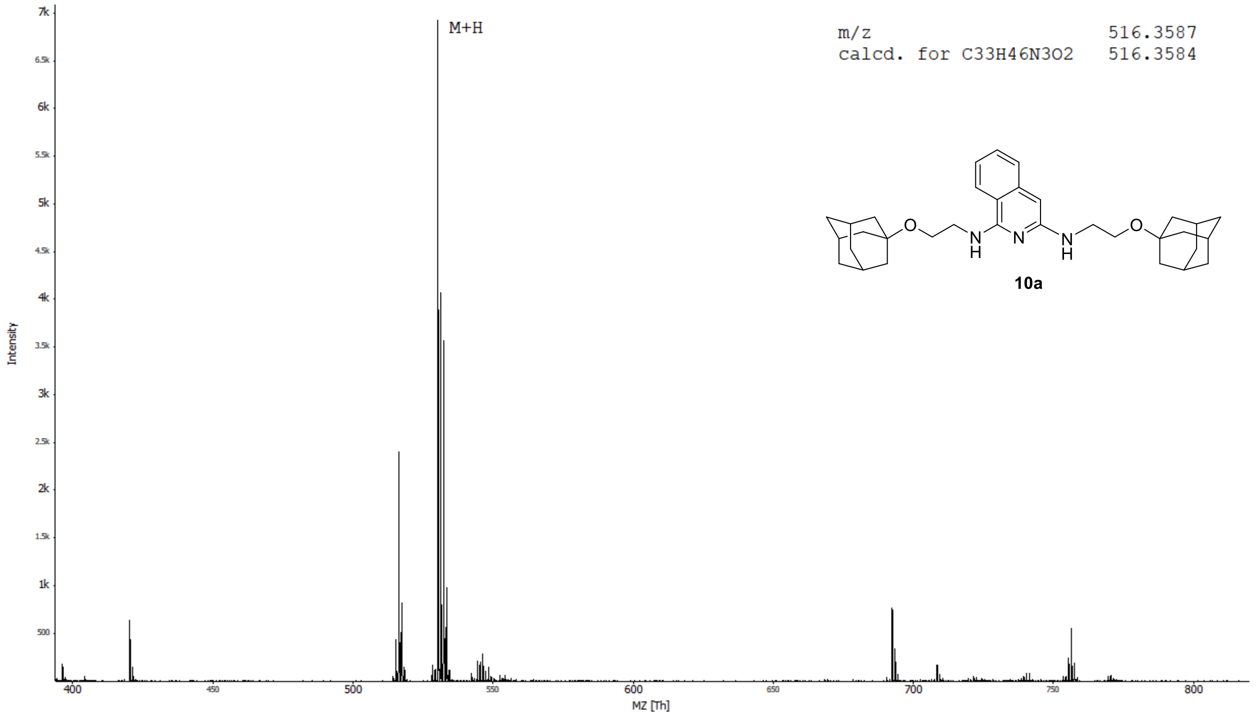

Figure S54. MALDI-TOF spectra of the compound **10a**. Matrix: 1,8,9-trihydroxyanthracene. Calibration standard: PEG-400+ PEG-600.

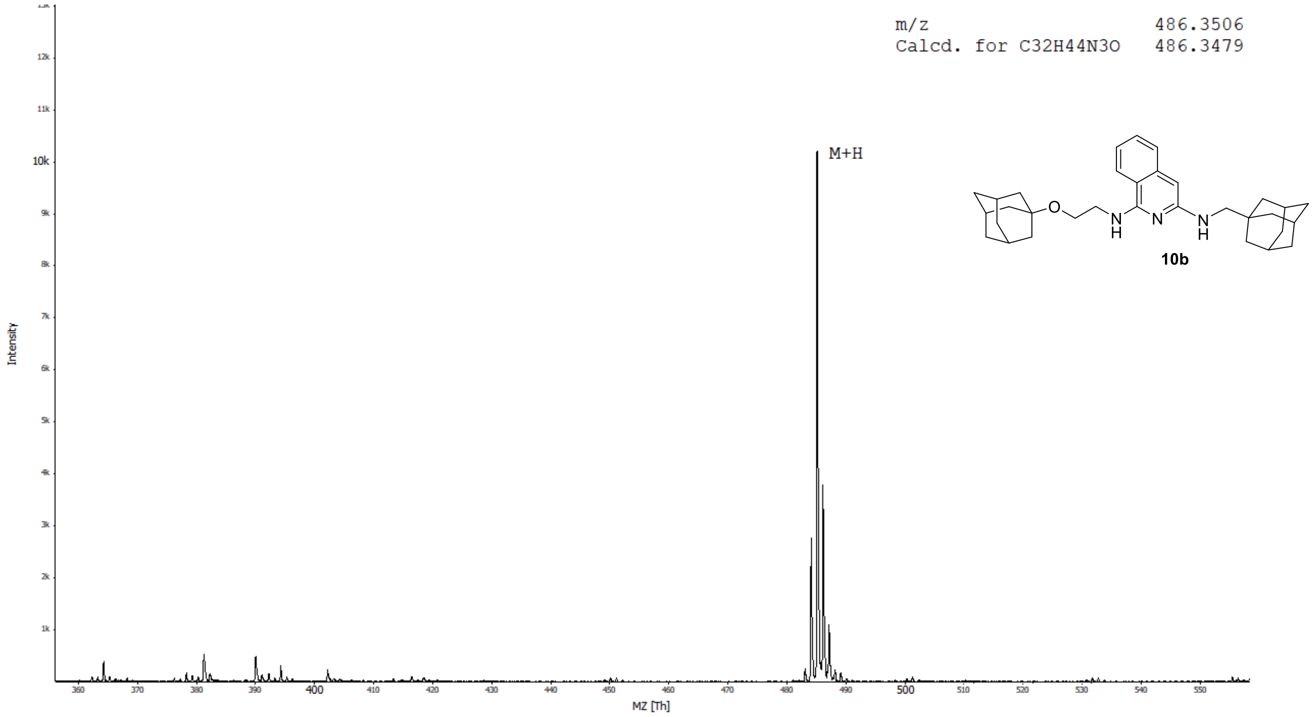

Figure S55. MALDI-TOF spectra of the compound **10b**. Matrix: 1,8,9-trihydroxyanthracene. Calibration standard: PEG-400.

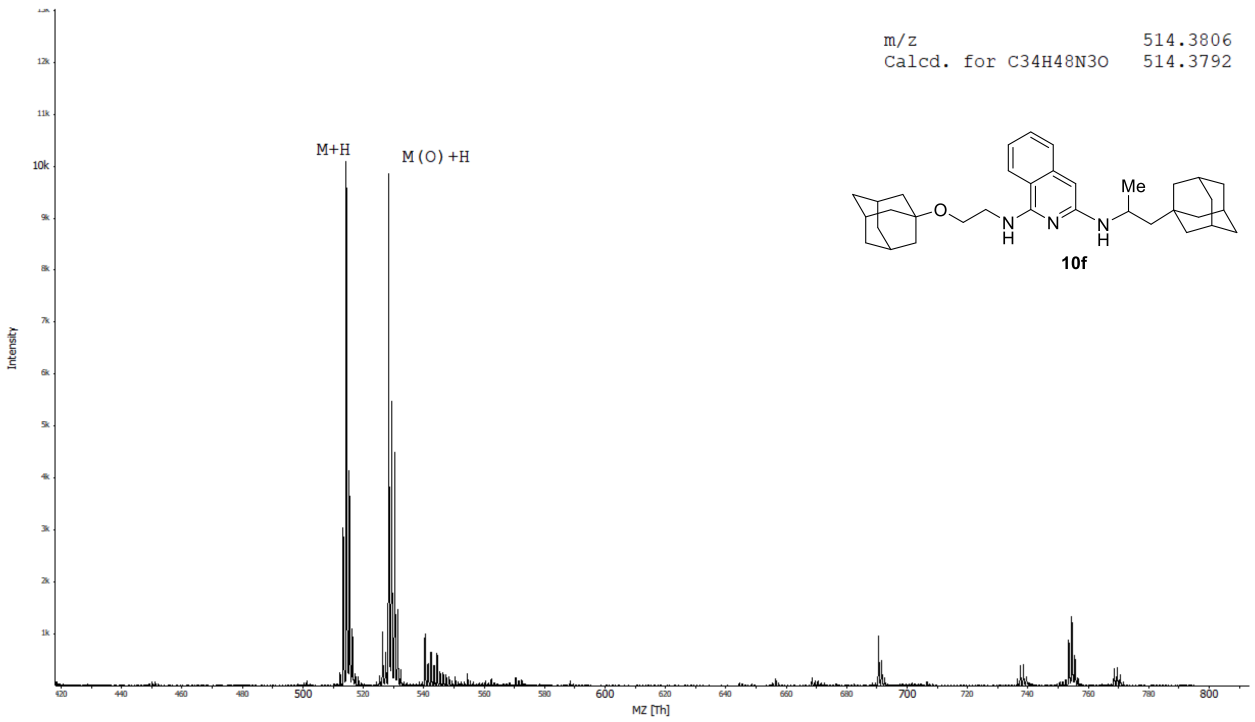

Figure S56. MALDI-TOF spectra of the compound **10f**. Matrix: 1,8,9-trihydroxyanthracene. Calibration standard: PEG-400+ PEG-600.

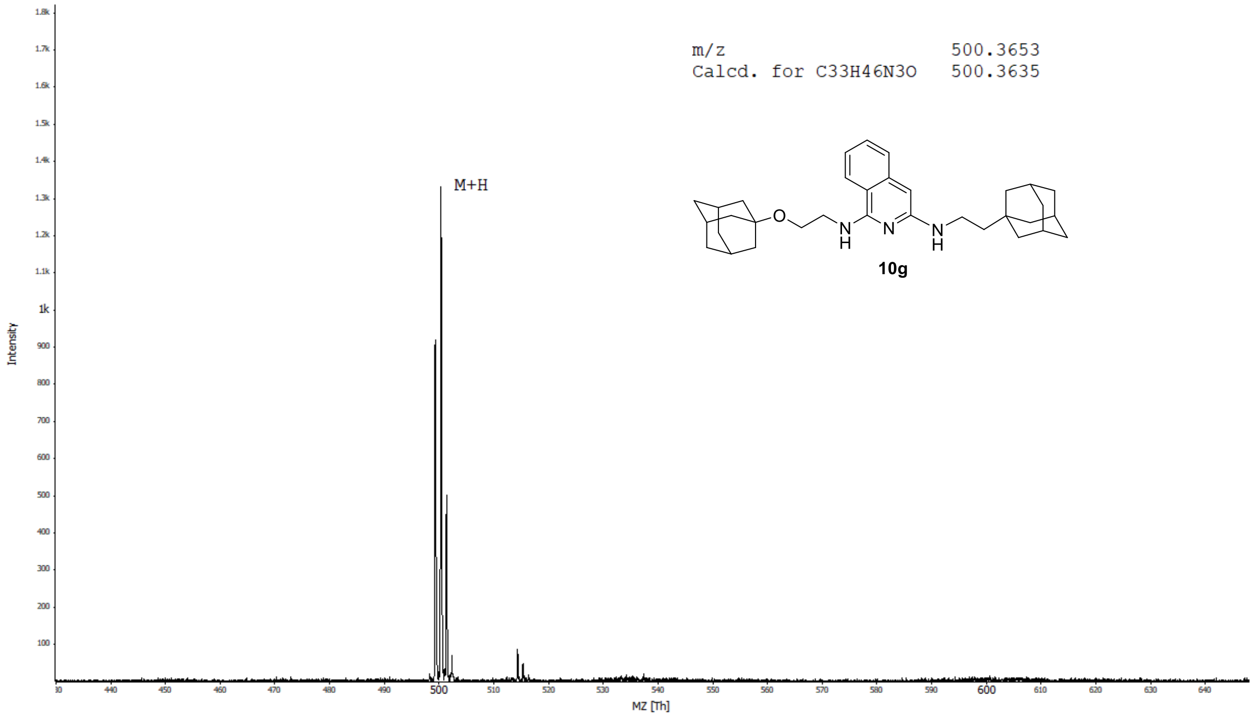

Figure S57. MALDI-TOF spectra of the compound **10g**. Matrix: 1,8,9-trihydroxyanthracene. Calibration standard: PEG-400+ PEG-600.
